# Coenzyme A activates TLR4 and enhances alternative macrophage activation in line with MyD88 agonism

**DOI:** 10.1038/s44319-026-00925-y

**Published:** 2026-09-18

**Authors:** Anthony E Jones, Amy Rios, Neira Ibrahimovic, Andréa B Ball, Carolina Chavez, Nicholas A Bayley, Natalie A Falta, Marisa Mekkittikul, Clarissa Carrillo, Wei Yuan Hsieh, Alessandro Sammarco, Amber B Reyes, Angel A Cortez, Thomas G Graeber, Richard L Watson, Alexander Hoffmann, Steven J Bensinger, Ajit S Divakaruni

**Affiliations:** 1 Departments of Molecular and Medical Pharmacology, University of California, Los Angeles, CA 90095 USA; 2 Microbiology, Immunology, and Molecular Genetics, University of California, Los Angeles, CA 90095 USA; 3 Medicine, Division of Pulmonary and Critical Care Medicine; University of California, Los Angeles, CA 90095 USA

**Keywords:** Immunology, Metabolism, Signal Transduction

## Abstract

Metabolites and metabolic cofactors can shape the innate immune response, though the pathways by which these molecules adjust inflammation remain incompletely understood. Here we show that the metabolic cofactor coenzyme A (CoA) enhances IL-4 driven alternative macrophage activation [M(IL-4)] in vitro and in vivo. Unexpectedly, we find that perturbations in intracellular CoA metabolism do not influence M(IL-4) differentiation. Rather, we discover that exogenous CoA is a weak TLR4 agonist which primes macrophages for increased receptivity to IL-4 signals and resolution of inflammation via MyD88. Mechanistic studies reveal MyD88-linked signals enhance IL-4 responsiveness, in part, by reshaping chromatin accessibility to enhance transcription of IL-4-linked genes. The results identify CoA as a host metabolic co-factor that influences macrophage function through an extrinsic TLR4-dependent mechanism and suggest that damage-associated molecular patterns (DAMPs) can prime macrophages for alternative activation and resolution of inflammation.

## Synopsis

Coenzyme A (CoA) enhances macrophage anti-inflammatory responses by acting as a weak, extrinsic pro-inflammatory agonist that primes macrophages for IL-4 signaling. This elucidates previous work linking CoA to the IL-4 response and shows how metabolic cofactors can shape the innate immune response.

- Exogenous CoA provision enhances the macrophage IL-4 response [M(IL-4)] in vitro and in vivo.
- The effect of exogenous CoA on [M(IL-4)] is independent of intracellular CoA levels and many metabolic hallmarks of alternative activation.
- CoA is a weak TLR4 agonist.
- CoA enhances [M(IL-4)] similarly to other MyD88-linked TLR agonists.

## Abstract (web summary)

Coenzyme A (CoA) enhances macrophage anti-inflammatory responses by acting as a weak, extrinsic pro-inflammatory agonist that primes macrophages for IL-4 signaling. This elucidates previous work linking CoA to the IL-4 response and shows how metabolic co-factors can shape the innate immune response.

## Graphical abstract

**Figure:**
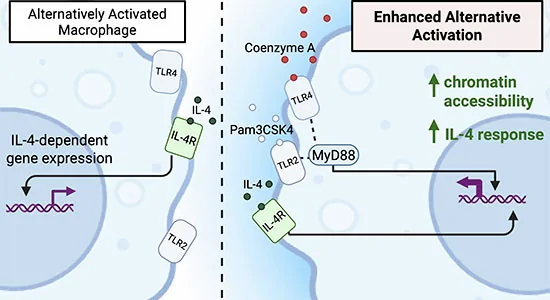

## Introduction

Macrophages are innate immune cells that execute a variety of functions, such as detecting and removing foreign pathogens, instructing adaptive immune cell function, secreting cytokines, and maintaining tissue homeostasis. To fulfill these diverse roles, macrophages link the detection of distinct external cues with the engagement of specific transcriptional programs to support different functional states. This process is commonly termed macrophage activation or “polarization” (Glass and Natoli, 2016; Murray, 2017).

In vitro studies often consider macrophage polarization in two discrete states: classical (Type 1) and alternative (Type 2) activation (Murray et al, 2014). Classical activation is associated with antimicrobial immunity and occurs when macrophages detect pathogen-associated molecular patterns (PAMPs) such as lipopolysaccharide (LPS) or damage-associated molecular patterns (DAMPs). These pro-inflammatory stimuli are detected by pattern recognition receptors such as Toll-like receptors (TLRs), and the signals are relayed and modulated intracellularly via adapter proteins such as myeloid differentiation primary response protein 88 (MyD88) and TIR-domain-containing adapter-inducing interferon-β (TRIF). Alternative activation is associated with wound healing, allergen response, and anti-helminth immunity. It is initiated by the macrophage response to interleukin-4 (IL-4) [± interleukin-13 (IL-13)], and largely driven by the signal transducer and activator of transcription 6 (STAT6) transcription factor.

Along with transcriptional programs that support core macrophage functions such as cytokine secretion and phagocytosis, changes in cellular metabolism are also important and perhaps essential for macrophage effector function (Wculek et al, 2022; Jones and Divakaruni, 2020; Van den Bossche et al, 2017). Upon classical, pro-inflammatory activation, macrophages increase glycolysis, generate metabolic signals thought to amplify the immune response, and shift away from oxidative phosphorylation in response to strong stimuli such as high concentrations of LPS (Mills et al, 2016; Haschemi et al, 2012; Tannahill et al, 2013; Seim et al, 2019). Conversely, alternatively activated macrophages have increased rates of oxidative phosphorylation and fatty acid oxidation (Huang et al, 2016; Vats et al, 2006), though evidence is mixed as to whether these changes are essential for the macrophage IL-4 response [M(IL-4)] (Van den Bossche and van der Windt, 2018; Divakaruni et al, 2018; van Teijlingen Bakker et al, 2022). The mechanistic links between how increased oxidative phosphorylation and/or fatty acid oxidation could specifically support M(IL-4) are also unclear. Proposed mechanisms, though, include changes in histone acetylation from enhanced acetyl-CoA production (Covarrubias et al, 2016) as well as transcriptional changes that respond to the mitochondrial membrane potential (Sanin et al, 2018).

Previous work in our laboratory has associated intracellular levels of coenzyme A (CoA) with the macrophage IL-4 response (Jones et al, 2021; Divakaruni et al, 2018). When trying to identify why excess concentrations of the carnitine palmitoyltransferase-1 (CPT-1) inhibitor etomoxir blocked M(IL-4) but genetic ablation of either *Cpt1* or *Cpt2* did not (Gonzalez-Hurtado et al, 2017; Van den Bossche and van der Windt, 2018), we discovered that excess etomoxir disrupted macrophage CoA homeostasis. In support of this as a putative mechanism, provision of exogenous CoA restored both intracellular CoA levels as well as the expression of IL-4-linked cell surface markers (Divakaruni et al, 2018). However, precisely how CoA instructs macrophage activation was not studied.

Here, we demonstrate that CoA augments alternatively activated macrophage function via weak TLR4 agonism and MyD88-linked signaling, a pathway commonly associated with pro-inflammatory activation. In investigating the mechanism by which exogenous CoA regulates M(IL-4), we surprisingly discovered that CoA provision did not act either by changing intracellular CoA levels or by enhancing known metabolic hallmarks of the IL-4 response. Rather, pharmacologic and genetic approaches showed exogenous CoA is a weak TLR4 agonist and boosts M(IL-4) by activating MyD88-linked signaling. MyD88 agonism was sufficient to enhance in vitro and in vivo alternative activation and increase chromatin accessibility at the promoter regions of IL-4-target genes. The data show that (i) CoA is a TLR4 agonist, (ii) the IL-4 response can be enhanced despite reversing many of the metabolic hallmarks of alternative activation, and (iii) pro-inflammatory MyD88-linked signaling can support M(IL-4) and resolution of inflammation. Furthermore, the results suggest CoA can act as a damage-associated molecular pattern (DAMP) that primes macrophages for the resolution of inflammation by an extrinsic TLR4-dependent mechanism.

## Results

### Exogenous CoA provision enhances alternative macrophage activation in vitro and in vivo

**Figure 1:**
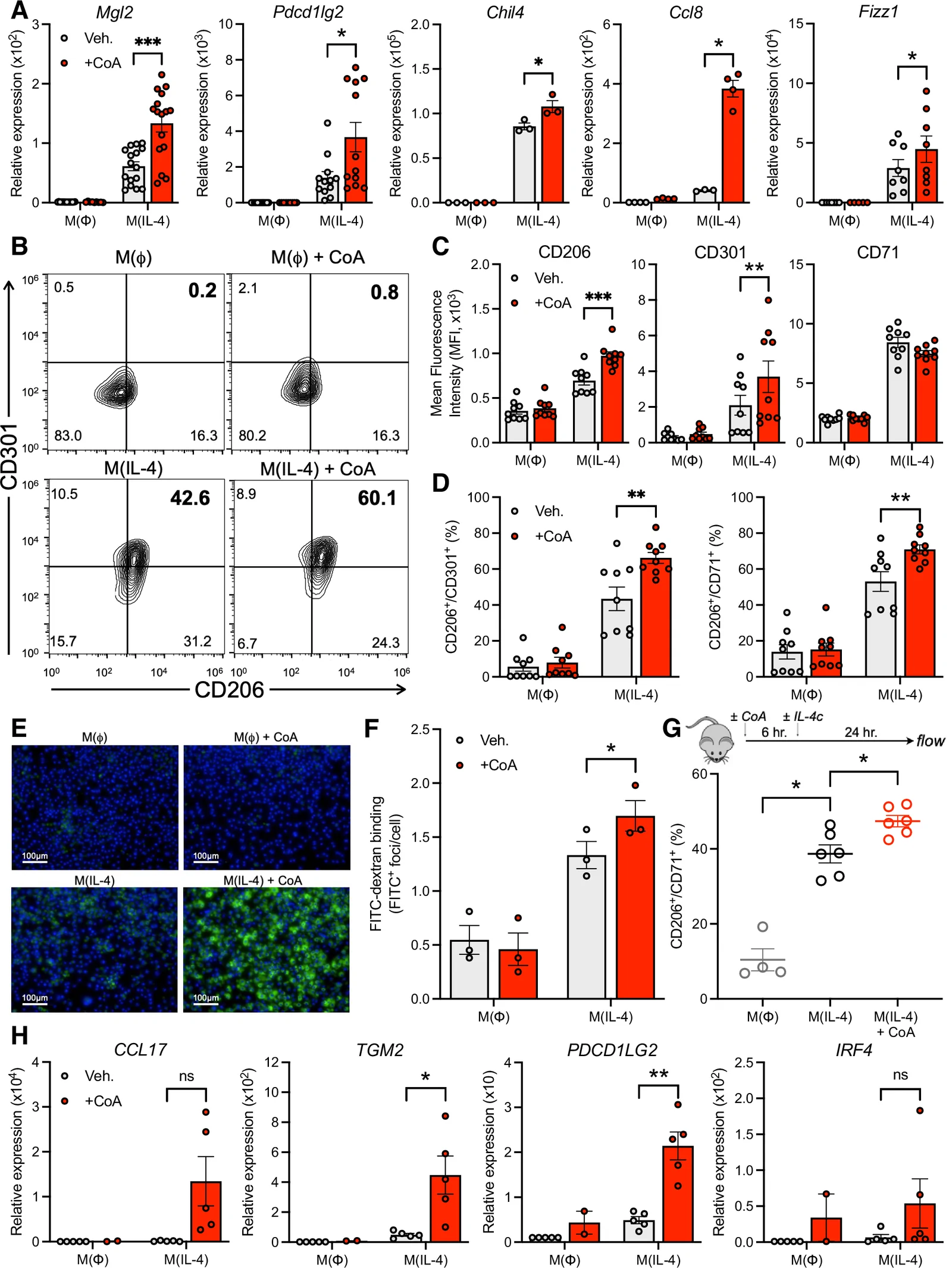
Exogenous CoA provision enhances alternative macrophage activation. (**A**) qPCR analysis of the IL-4-associated genes *Mgl2*, *Pdcd1lg2*, *Chil4*, *Ccl8*, and *Fizz1* in BMDMs treated with CoA (1 mM), IL-4 (20 ng/mL), CoA + IL-4, or vehicle (Veh.) for 48 h. (*n* ≥ 9 independent biological replicates) (***P < 0.001, *P = 0.02 *Pdcd1gl2*, *P = 0.03 *Chil4*, *P = 0.02 *Ccl8*, *P = 0.03 *Fizz1*). (**B**–**D**) Flow cytometric analysis of the IL-4-associated cell surface markers CD206, CD301, and CD71 after treatments as in (**A**). (**B**) Contour plots with the percentage of cells expressing both CD206 and CD301 are indicated in the upper right quadrant. Data shown are from a single representative experiment. (**C**) Aggregate mean fluorescence intensity of CD206, CD301, and CD71 (*n* = 9 independent biological replicates) (***P < 0.001, **P = 0.003). (**D**) Percentages of CD206^+^/CD301^+^ and CD206^+^/CD71^+^ populations (*n* = 9 independent biological replicates) (**P = 0.001 CD206^+^/CD301^+^, **P = 0.003 CD206^+^/CD71^+^). (**E**) Representative images of BMDMs incubated for 1 h with FITC-dextran (1 mg/mL, green) and Hoechst 33342 (10 ng/mL, blue) after stimulation with compounds as in (**A**). (**F**) Aggregate image analysis data for experiments as in (**E**) (*n* = 3 independent biological replicates) (*P = 0.04). (**G**) Quantification of CD206^+^/CD71^+^ peritoneal macrophages from mice that were exposed to vehicle (PBS), IL-4c (5 µg IL-4 and 25 µg anti-IL-4 monoclonal antibody), or the combination of IL-4c + CoA. (*n* ≥ 3 mice were used for each group) (*P = 0.03 for both comparisons). (**H**) qPCR analysis of the IL-4 associated genes *CCL17, TGM2, PDCD1LG2, and IRF4* in HMDMs treated as in (**A**) (*n* = 5 independent biological replicates) (*P = 0.03, **P = 0.003, ns not significant). All data were presented as mean ± SEM with statistical analysis conducted on biological replicates, each of which included multiple technical replicates unless otherwise indicated. Statistical analysis was performed as a one-way ANOVA followed by either Dunnett’s post hoc multiple comparisons test (**A**, **C**) or Šídák’s post hoc multiple comparisons test (**D**,** F**–**G**). Mixed effects analysis was used for data sets without uniformly matched samples. A paired *t*-test was used for analysis in (**H**), as MΦ samples were not included in the statistical analysis due to insufficient sample size. Source data are available online for this figure.

**Figure EV1:**
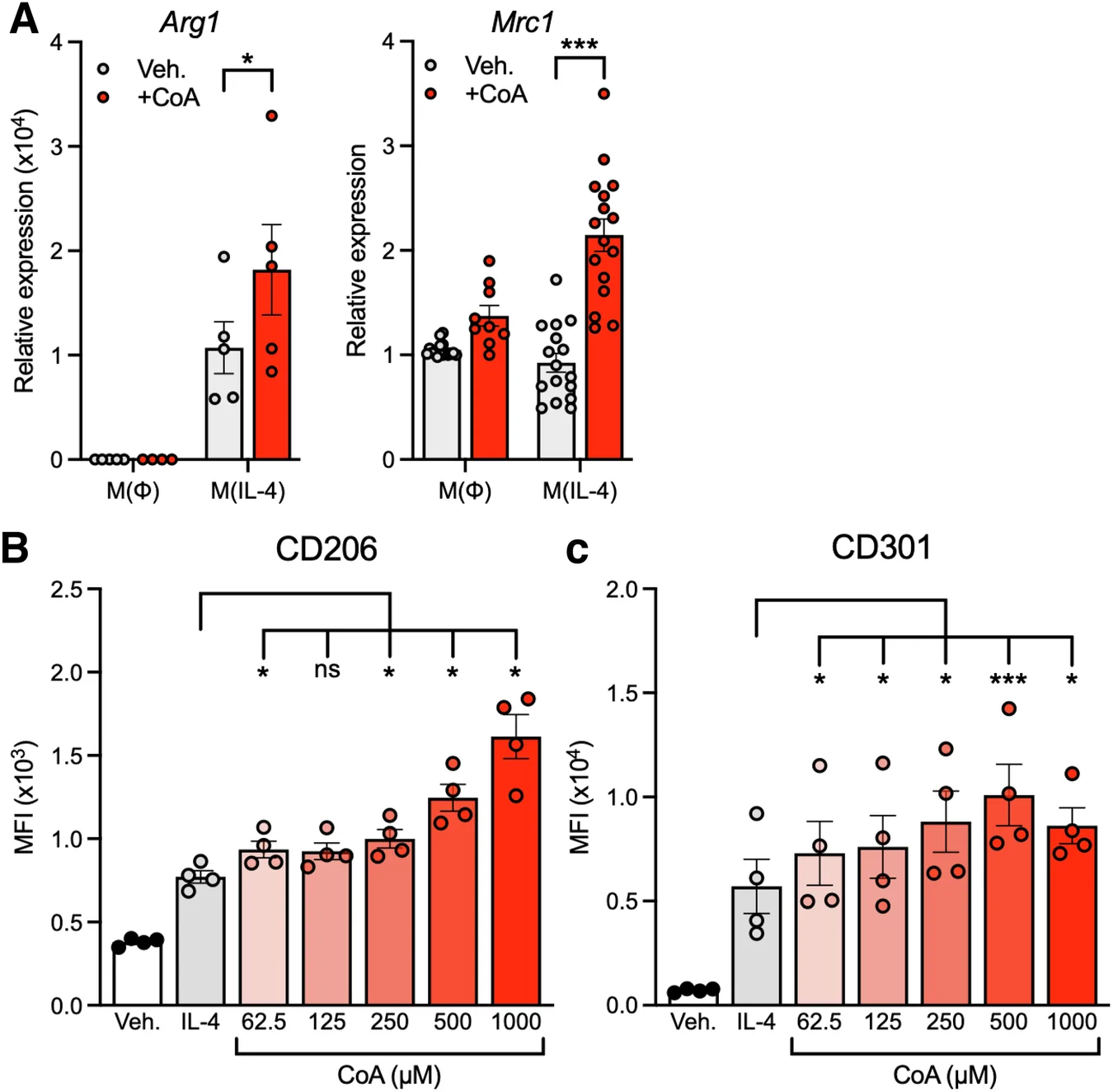
Exogenous CoA provision enhances alternative macrophage activation. (**A**) qPCR analysis of the IL-4-associated genes *Arg1 and Mrc1* in BMDMs treated with vehicle (Veh.), CoA (1 mM), IL-4 (20 ng/mL), or CoA + IL-4 for 48 h. (*n* ≥ 9 independent biological replicates) (*P = 0.02, ***P < 0.001). (**B**,** C**) Mean fluorescence intensity of (**B**) CD206 and (**C**) CD301 in BMDMs following treatment with vehicle, IL-4 (20 ng/mL), or IL-4 + CoA with concentrations as indicated in the figure for 48 h. (*n* = 4 independent biological replicates) (CD206: *P = 0.04 62.5 µM, *P = 0.02 250 µM, *P = 0.01 500 µM, *P = 0.02 1000 µM; CD301: *P = 0.03 62.5 µM, *P = 0.01 125 µM, *P = 0.01 250 µM, ***P < 0.001, *P = 0.03 1000 µM). All data were presented as mean ± SEM with statistical analysis conducted on biological replicates, each of which included multiple technical replicates, unless otherwise indicated. Statistical analysis for (**A**) was performed as a paired, two-tailed *t-*test. Statistical analysis for (**B**,** C**) was performed as a one-way ANOVA followed by Dunnett’s post hoc multiple comparisons test. ns no significance. Source data are available online for this figure.

Prior work had shown that exogenous CoA could rescue the etomoxir-mediated inhibition of M(IL-4), but the mechanisms underlying this effect were not defined. To better understand how exogenous CoA influenced alternative macrophage activation, mouse bone marrow-derived macrophages (BMDMs) were treated with IL-4 alone or in combination with CoA for 48 h. Gene expression studies revealed that CoA enhanced the expression of multiple IL-4-associated genes, including *Mgl2, Pdcd1lg2, Chil4, Ccl8, Fizz1, Arg1, and Mrc1* (Figs. 1A and EV1A) (Webb et al, 2001; Zhang et al, 2020; Liu et al, 2017; Sanin et al, 2018). Flow cytometry analysis revealed a similar relationship when measuring IL-4-linked cell surface marker expression. CoA increased the mean fluorescence intensity of CD206 and CD301, as well as the frequency of CD206^+^/CD301^+^ and CD206^+^/CD71^+^ BMDMs (Fig. 1B–D). This effect was observed with CoA concentrations as low as 62.5 µM (Fig. EV1B,C). Importantly, CoA itself did not stimulate the expression of IL-4-associated genes or cell surface markers (Fig. 1A–D), demonstrating it is not an IL-4 receptor agonist but rather acts cooperatively to enhance macrophage alternative activation.

Mannose receptor activity is essential for the initiation of the T_H_2 response during helminth infection (van Die and Cummings, 2017; van Liempt et al, 2007). Given the observed increase in its gene (*Mrc1*) and protein (CD206) expression, we measured the effect of exogenous CoA on the activity of the mannose receptor. As expected, high-content imaging revealed that CoA enhanced the cellular uptake of FITC-dextran, a fluorescently labeled mannose receptor ligand (Sallusto et al, 1995) (Fig. 1E,F).

To assess whether CoA could enhance alternative activation in vivo, mice were injected intraperitoneally (i.p.) with IL-4 complex (IL-4c) in the presence or absence of CoA (40 mg/kg). After one day, the peritoneum was flushed and the frequency of CD206^+^/CD71^+^ peritoneal macrophages was assessed. Indeed, CoA enhanced the fraction of macrophages co-expressing CD206 and CD71 (Fig. 1G). We also determined whether CoA could enhance the IL-4 response in human monocyte-derived macrophages (HMDMs). Co-treatment of HMDMs with IL-4 and CoA dramatically increased the expression of the IL-4-linked genes *CCL17*, *TGM2,* and *PDCD1LG2* (similarly to mouse macrophages), and IRF4 (Fig. 1H). Altogether, the data show exogenous CoA provision augments M(IL-4) in vitro and in vivo, and in both mouse- and human-derived macrophages.

### CoA does not augment alternative macrophage activation by enhancing metabolic hallmarks of M(IL-4)

We next sought to identify the mechanism by which CoA enhances M(IL-4). Several metabolic hallmarks of alternative activation require CoA as a necessary cofactor, including enhanced mitochondrial respiratory capacity (Van den Bossche et al, 2016; Puleston et al, 2019; Tan et al, 2015), mitochondrial pyruvate oxidation (Huang et al, 2016; Jha et al, 2015), and de novo lipid synthesis (Huang et al, 2014; Bidault et al, 2021). As such, we hypothesized that the addition of exogenous CoA enhanced the IL-4 response by increasing intracellular CoA levels to support flux through these metabolic pathways (Gout, 2018; Pietrocola et al, 2015; Leonardi et al, 2005).

**Figure 2:**
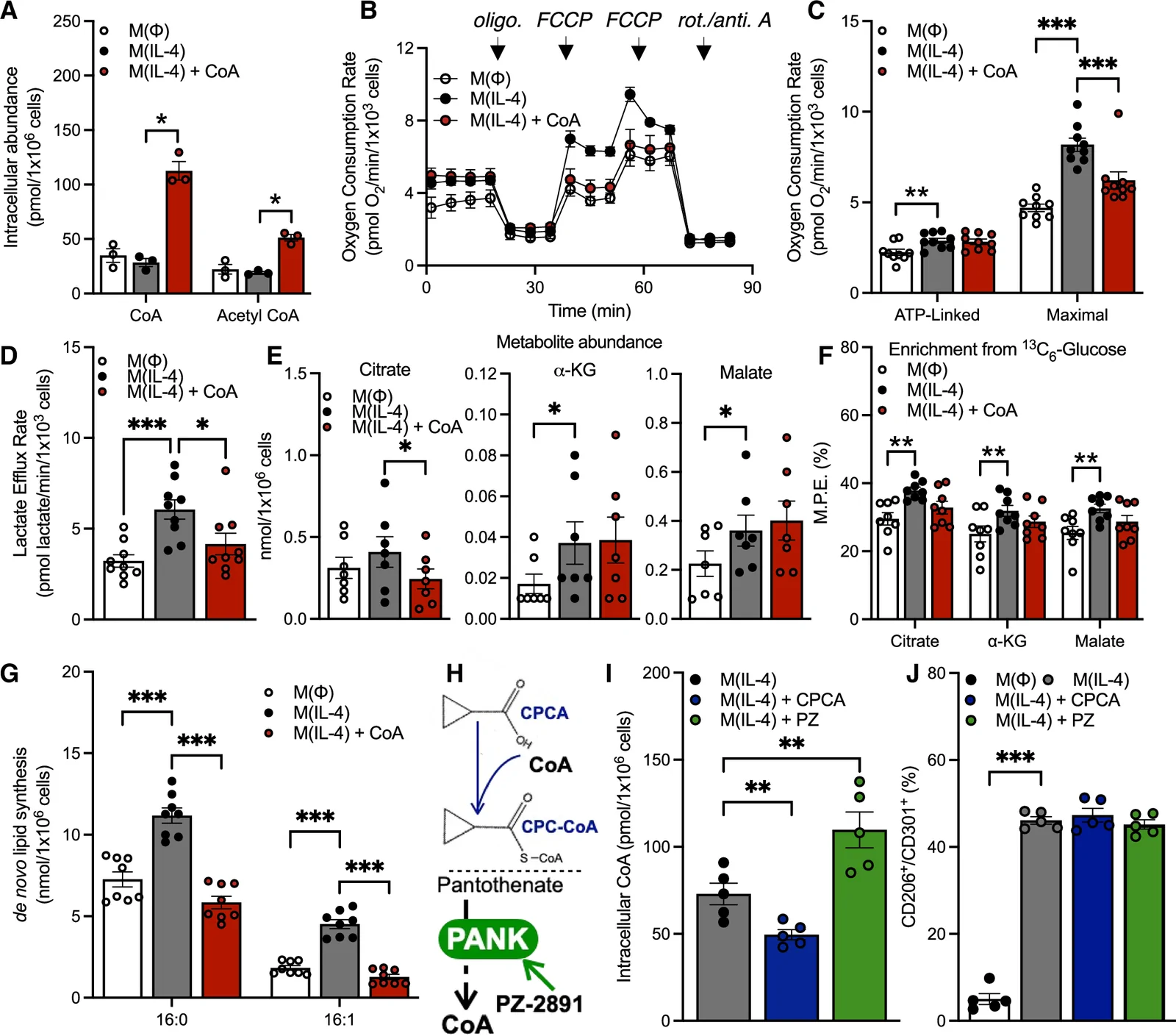
CoA does not enhance alternative macrophage activation by boosting known metabolic hallmarks of the IL-4 response. (**A**) Intracellular levels of CoA and acetyl-CoA in BMDMs measured by LC-MS/MS. Cells were treated with IL-4 (20 ng/mL), IL-4 + CoA (1 mM), or vehicle [M(ϕ)] for 48 h as in Fig. 1 (*n* = 3 independent biological replicates) (*P = 0.01 CoA, *P = 0.01 Acetyl-CoA). (**B**) Representative respirometry trace of BMDMs treated as in (**A**). (*n* = 5 technical replicates from a single biological replicate). When not visible, error bars are obscured by the symbol. Oligo. oligomycin, rot./anti.A rotenone/antimycin. (**C**) Aggregate ATP-linked and FCCP-stimulated respiration in intact BMDMs for treatments as in (**A**). Cells were offered 8 mM glucose, 2 mM pyruvate, and 2 mM glutamine in the experimental medium (*n* = 8 independent biological replicates) (**P = 0.001, ***P < 0.001). (**D**) Lactate efflux rate from respirometry experiments as in (**B**, **C**) calculated using Seahorse XF data and correcting for respiratory CO_2_ (*n* = 8 independent biological replicates) (***P < 0.001, *P = 0.02). (**E**) Metabolite abundances of citrate, α-ketoglutarate (α-KG), and malate in BMDMs treated as in (**A**) (*n* = 7 independent biological replicates) (*P = 0.02 citrate, *P = 0.03 α-KG, *P = 0.02 malate). (**F**) Mole percent enrichment (M.P.E.) from uniformly labeled ^13^C_6_-glucose into the TCA cycle intermediates as in (**E**) (*n* = 8 independent biological replicates) (**P = 0.004 citrate, **P = 0.003 α-KG, **P = 0.003 malate). (**G**) Quantification of newly synthesized palmitic acid (16:0) and palmitoleic acid (16:1) from BMDMs stimulated as in (**A**). (data shown as *n* = 8 technical replicates from *n* = 2 independent biological replicates) (***P < 0.001). (**H**) Schematic depicting the mechanism of action of cyclopropane carboxylic acid (CPCA) and PZ-2891 (PZ). (**I**) Intracellular CoA levels of BMDMs stimulated with IL-4, IL-4 + CPCA (1 mM), or IL-4 + PZ (10 µM) for 48 h (*n* = 5 independent biological replicates) (**P = 0.005 CPCA, **P = 0.002 PZ). (**J**) Flow cytometric quantification of the CD206^+^/CD301^+^ population for BMDMs treated as in (**I**) (*n* = 5 independent biological replicates) (***P < 0.001). All data were presented as mean ± SEM with statistical analysis conducted on biological replicates, each of which included multiple technical replicates, unless otherwise indicated. Statistical analysis for (**A**, **C**–**G**, **I**, **J**) was performed as an ordinary one-way ANOVA followed by Dunnett’s post hoc multiple comparisons test. Source data are available online for this figure.

We first confirmed that exogenously added CoA could expand the intracellular CoA pool. Supplementing culture medium with CoA increased the steady-state abundances of both intracellular CoA and acetyl-CoA (Fig. 2A). Next, we investigated whether CoA provision enhanced IL-4-driven increases in respiration and glycolysis (Huang et al, 2016, 2014; Van den Bossche et al, 2016). Similar to other reports, we observed increases in ATP-linked respiration, maximal respiratory capacity, and glycolysis with IL-4. However, the addition of CoA did not further augment these metabolic changes and even limited the glycolytic rate and maximal respiratory capacity of alternatively activated macrophages (Fig. 2B–D).

**Figure EV2:**
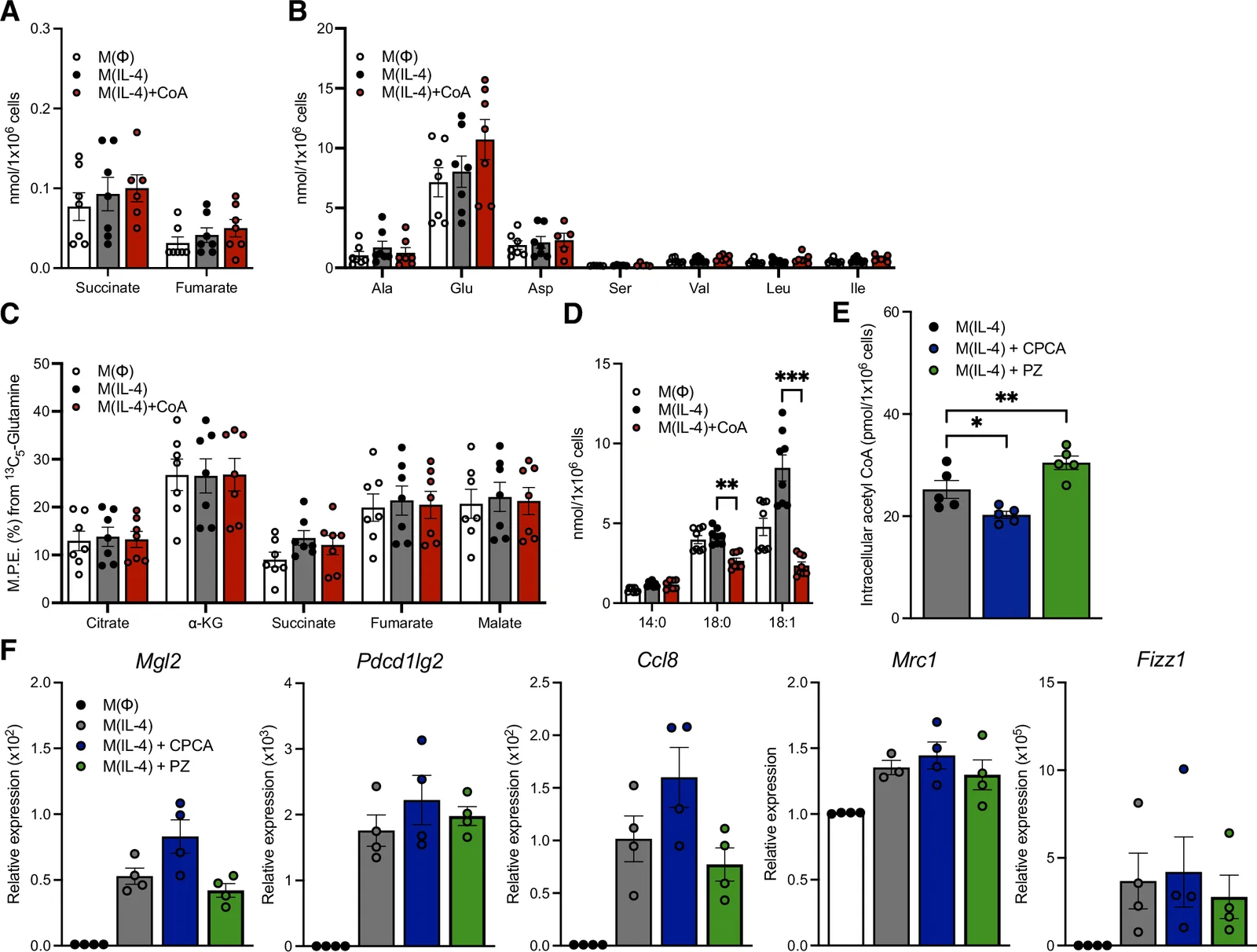
CoA does not enhance alternative activation by increasing metabolic changes associated with the IL-4 response. (**A**,** B**) Abundances of (**A**) TCA cycle intermediates and (**B**) amino acids in BMDMs treated with vehicle (Veh.), IL-4 (20 ng/mL), or IL-4 + 1 mM CoA for 48 h. (*n* = 7 independent biological replicates). (**C**) Enrichment from uniformly labeled ^13^C_5_-glutamine into the TCA cycle intermediates citrate, α-ketoglutarate (α-KG), succinate, fumarate, and malate in BMDMs treated as in (**A**) (*n* = 7 independent biological replicates). (**D**) Quantification of newly synthesized myristic acid (14:0), stearic acid (18:0), and oleic acid (18:1) from BMDMs treated as in (**A**) (data shown as *n* = 8 technical replicates from *n* = 2 independent biological replicates) (**P = 0.010, ***P < 0.001). (**E**) Intracellular acetyl-CoA levels of BMDMs stimulated with vehicle [M(ϕ)], IL-4 (20 ng/mL), IL-4 + CPCA (1 mM), or IL-4 + PZ-2891 (10 µM) for 48 h (*n* = 5 independent biological replicates) (*P = 0.02, **P = 0.006). (**F**) qPCR analysis of the IL-4-associated genes *Mgl2, Pdcd1lg2, Ccl8, Mrc1, and Fizz1* in BMDMs treated as in (**E**) (*n* = 4 independent biological replicates). All data were presented as mean ± SEM with statistical analysis conducted on biological replicates, each of which included multiple technical replicates, unless otherwise indicated. Statistical analysis for (**D**) was performed as a two-way ANOVA followed by Tukey’s post hoc multiple comparisons test. Statistical analysis for (**E**) was performed as a one-way ANOVA followed by Dunnett’s post hoc multiple comparisons test. Source data are available online for this figure.

After determining that CoA does not enhance alternative macrophage activation via an expansion of bioenergetic capacity, we then examined whether CoA increased other IL-4-linked metabolic alterations, such as increased TCA cycle metabolite abundance (Jha et al, 2015), pyruvate oxidation (Huang et al, 2016; Tan et al, 2015), glutamine oxidation (Jha et al, 2015), and de novo lipogenesis (Bidault et al, 2021). Indeed, we reproduced previous reports that show IL-4 increases steady-state levels of select TCA cycle metabolites (Fig. 2E), enrichment from glucose into the TCA cycle (Fig. 2F), and de novo lipid synthesis (Fig. 2G). However, as before, addition of CoA did not further increase these metabolic changes (Figs. 2E–G and EV2A–C), and even blocked the IL-4-stimulated increase in fatty acid synthesis (Figs. 2G and EV2D). As such, the results show exogenous CoA augments alternative activation by a mechanism discrete from metabolic reprogramming.

### Alterations in intracellular CoA levels are not sufficient to alter M(IL-4)

We then questioned whether changes in intracellular CoA levels, in fact, shape the IL-4 response. To answer this, we utilized two compounds with opposing impacts on intracellular CoA levels. To decrease steady-state CoA levels, we treated IL-4-stimulated BMDMs with cyclopropane carboxylic acid (CPCA), which decreases the abundance of “free” CoA as its cognate thioester CPC-CoA is formed (Jones et al, 2021; Steinhelper and Olson, 1985) (Fig. 2H). To increase steady-state CoA levels, we treated IL-4-polarized BMDMs with PZ-2891, a pantothenate kinase activator which enhances CoA biosynthesis (Sharma et al, 2018) (Fig. 2H). As expected, CPCA decreased intracellular levels of CoA and acetyl-CoA, while PZ-2891 increased their abundances (Figs. 2I and EV2E). Surprisingly, despite altering steady-state intracellular CoA levels, neither compound impacted gene expression or cell surface markers associated with alternative activation (Figs. 2J and EV2F). The results show that although exogenous CoA provision augments the macrophage IL-4 response, the mechanism cannot be attributed to changing intracellular levels of CoA and acetyl-CoA.

### Exogenous CoA induces a pro-inflammatory macrophage response in vitro and in vivo

**Figure 3:**
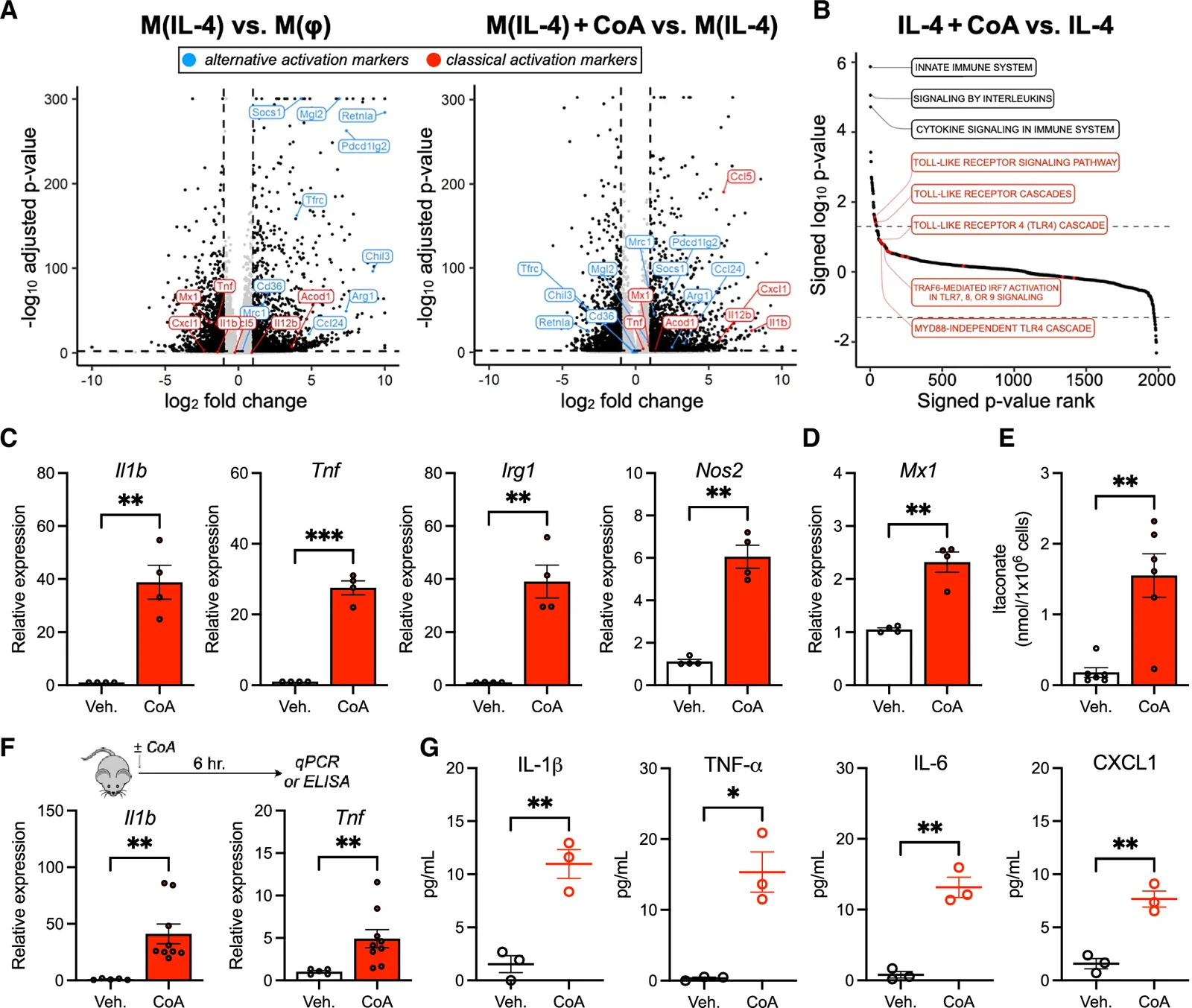
Exogenous CoA induces a pro-inflammatory response in BMDMs. (**A**) Volcano plot from bulk RNA sequencing data from BMDMs treated with IL-4 (20 ng/mL), IL-4 + CoA (1 mM), or vehicle control [M(ϕ)] for 48 h, comparing differential gene expression between IL-4 vs. vehicle controls (left) and IL-4 + CoA vs. IL-4 (right). Genes associated with classical activation are depicted in red, genes associated with alternative activation are shown in blue (*n* = 4 independent biological replicates). (**B**) Gene set enrichment analysis of genes differentially regulated in BMDMs treated with IL-4+CoA vs. IL-4 alone. (**C**) qPCR analysis of *Il1b*, *Tnf*, *Irg1*, and *Nos2* in BMDMs stimulated with CoA (1 mM) or vehicle control (Veh.) for 4 h (*n* = 4 independent biological replicates) (**P = 0.010 *Il1b*, ***P < 0.001, **P = 0.009 *Irg1*, **P = 0.002 *Nos2*). (**D**) qPCR analysis of the interferon-stimulated gene *Mx1* in BMDMs stimulated with 1 mM CoA or vehicle control for 24 h (*n* = 4 independent biological replicates) (**P = 0.006). (**E**) Itaconate abundance after treatment with 1 mM CoA or vehicle control for 48 h (*n* = 6 independent biological replicates) (**P = 0.006). (**F**) qPCR analysis of *Il1b* and *Tnf* in the peritoneal exudate cells of mice treated with vehicle control or (40 mg/kg) CoA 6 h prior to collection (*n* ≥ 5 mice for each group) (**P = 0.002 *Il1b*, **P = 0.007 *Tnf*). (**G**) Quantification of cytokines in the peritoneal lavage fluid (PLF) of mice treated as in (**F**) using Multiplexed ELISA (*n* = 3 mice were used for each group) (**P = 0.008 IL-1β, *P = 0.03, **P = 0.008 IL-6, **P = 0.004 CXCL1). All data were presented as mean ± SEM with statistical analysis conducted on biological replicates, each of which included multiple technical replicates, unless otherwise indicated. Statistical analysis for (**C**–**G**) was performed as an unpaired, two-tailed *t*-test. Source data are available online for this figure.

Neither metabolic alterations nor intracellular CoA levels could explain why CoA provision enhanced M(IL-4). Therefore, we sought a more complete understanding of how CoA impacts the transcriptome of alternatively activated macrophages by conducting bulk RNA sequencing (RNA-seq) on naïve macrophages alongside those stimulated with either IL-4 or IL-4 with CoA. As expected, cells stimulated only with IL-4 increased the expression of genes associated with alternative activation and decreased the expression of genes associated with classical activation relative to vehicle controls (Fig. 3A, left). In line with qPCR studies (Fig. 1A), CoA provision further increased the expression of IL-4-linked genes associated with alternative activation (Fig. 3A, right). Unexpectedly, however, CoA addition also increased the expression of genes associated with classical activation that were not associated with the IL-4 response (Fig. 3A, right). Subsequent Gene Set Enrichment Analysis (GSEA) showed that genes associated with TLR signaling pathways were upregulated upon co-treatment with CoA and IL-4 (Fig. 3B).

**Figure EV3:**
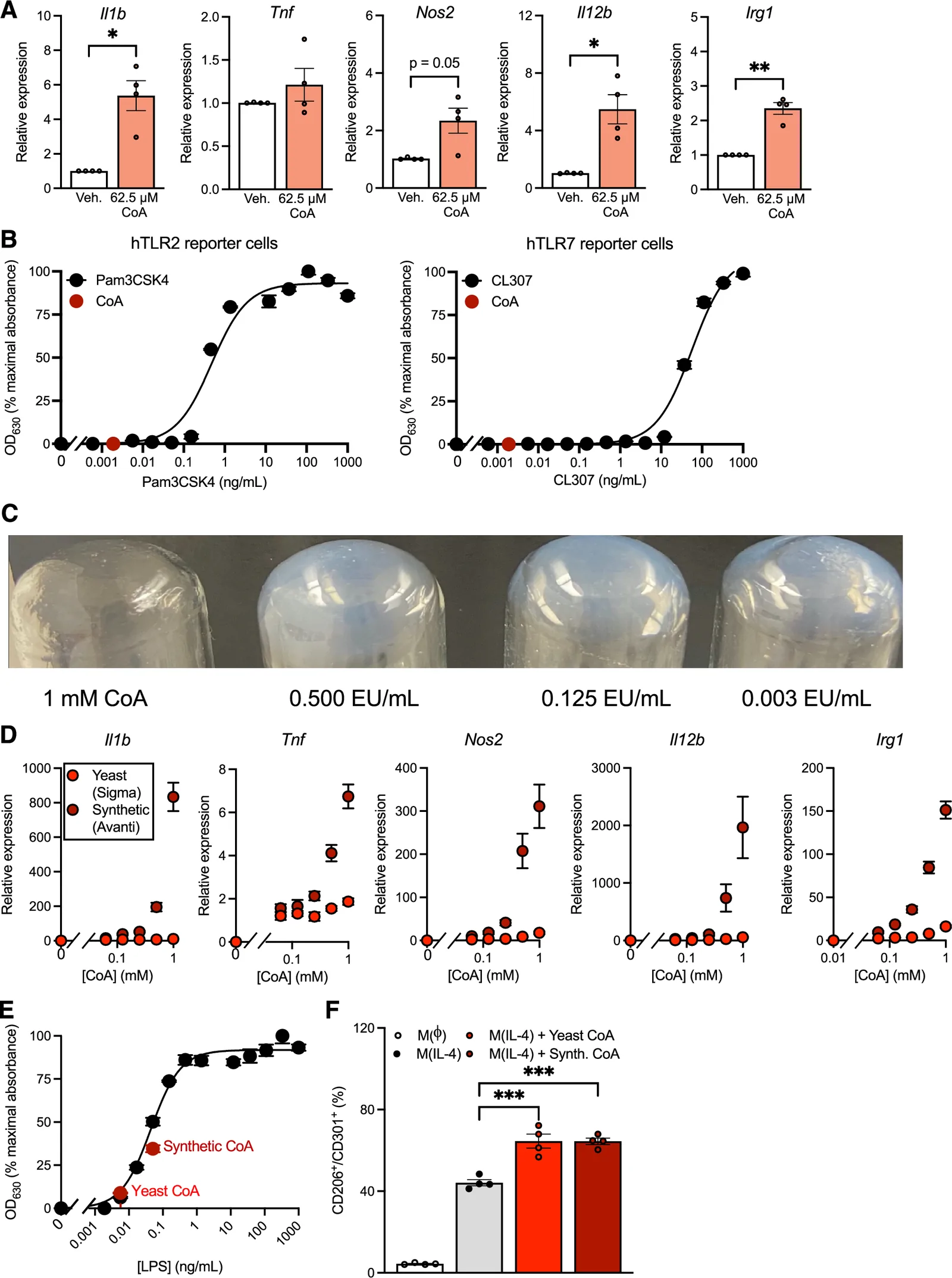
Coenzyme A increases pro-inflammatory gene expression. (**A**) qPCR analysis of the pro-inflammatory genes *Il1b, Tnf, Nos2, Il12b*, and *Irg1* in BMDMs treated with vehicle (Veh.) or 62.5 µM CoA for 4 h (*n* = 4 independent biological replicates) (*P = 0.02 *Il1b*, *P = 0.02 *Il12b*, **P = 0.004). (**B**) Concentration-response curves of hTLR2 and hTLR7 reporter cells exposed to increasing concentrations of Pam3CSK4 and CL307, respectively (black dots). The red dot indicates the response elicited from 1 mM CoA (*n* = 4 independent biological replicates). (**C**) Limulus test, with opaque precipitate signifying the presence of endotoxin, for 1 mM yeast-derived CoA alongside endotoxin standards. The translucent tube shows that CoA is free of endotoxin (EU, endotoxin units). (**D**) Gene expression analysis of the pro-inflammatory genes *Il1b, Tnf, Nos2, Il12b*, and *Irg1* in BMDMs treated with yeast-derived CoA (Sigma-Aldrich) or synthetic CoA (Avanti Polar Lipids) with concentrations as indicated in the figure for 4 h (*n* = 4 independent biological replicates). (**E**) Concentration-response curves of hTLR4 reporter cells exposed to increasing concentrations of LPS (black dots). The red dots indicate the responses elicited from 1 mM synthetic CoA and 1 mM yeast-derived CoA (*n* = 3 independent biological replicates). (**F**) Percentage of CD206^+^/CD301^+^ population in BMDMs treated with vehicle [M(ϕ)], IL-4 (20 ng/mL), IL-4 + 1 mM yeast-derived CoA, or IL-4 + 1 mM synthetic CoA for 48 h (*n* = 4 independent biological replicates) (***P < 0.001). All data were presented as mean ± SEM with statistical analysis conducted on biological replicates, each of which included multiple technical replicates, unless otherwise indicated. Statistical analysis for (**A**) was performed as an unpaired, two-tailed *t-*test. Statistical analysis for (**F**) was performed as an ordinary one-way ANOVA followed by Dunnett’s post hoc multiple comparisons test. Source data are available online for this figure.

This unbiased approach suggested that CoA may elicit a pro-inflammatory response in BMDMs along with its ability to enhance M(IL-4). To confirm this, we assessed whether CoA itself could induce expression of pro-inflammatory genes in the absence of IL-4. Indeed, exogenous CoA induced expression of *Il1b*, *Tnf*, *Irg1*, and *Nos2* (Fig. 3C), genes linked to the TLR adapter protein MyD88, as well as the interferon-stimulated gene (ISG) *Mx1* (Fig. 3D). As *Irg1* encodes the enzyme generating the antimicrobial metabolite itaconate, we also observed a ~10-fold increase in itaconate synthesis upon CoA provision (Fig. 3E). The induction of pro-inflammatory genes was also observed at 62.5 µM CoA, matching the lowest tested concentration of CoA that augments M(IL-4) (Figs. EV1B,C and EV3A). Lastly, i.p. administration of CoA in mice increased expression of *Il1b* and *Tnf* in peritoneal leukocytes and increased the abundance of IL-1β, TNF-α, IL-6, and CXCL1 in the peritoneal lavage fluid (Fig. 3F,G). Taken together, the results demonstrate that CoA, in addition to enhancing the IL-4 response, elicits a pro-inflammatory response in vitro and in vivo.

### CoA is a weak TLR4 agonist

**Figure 4:**
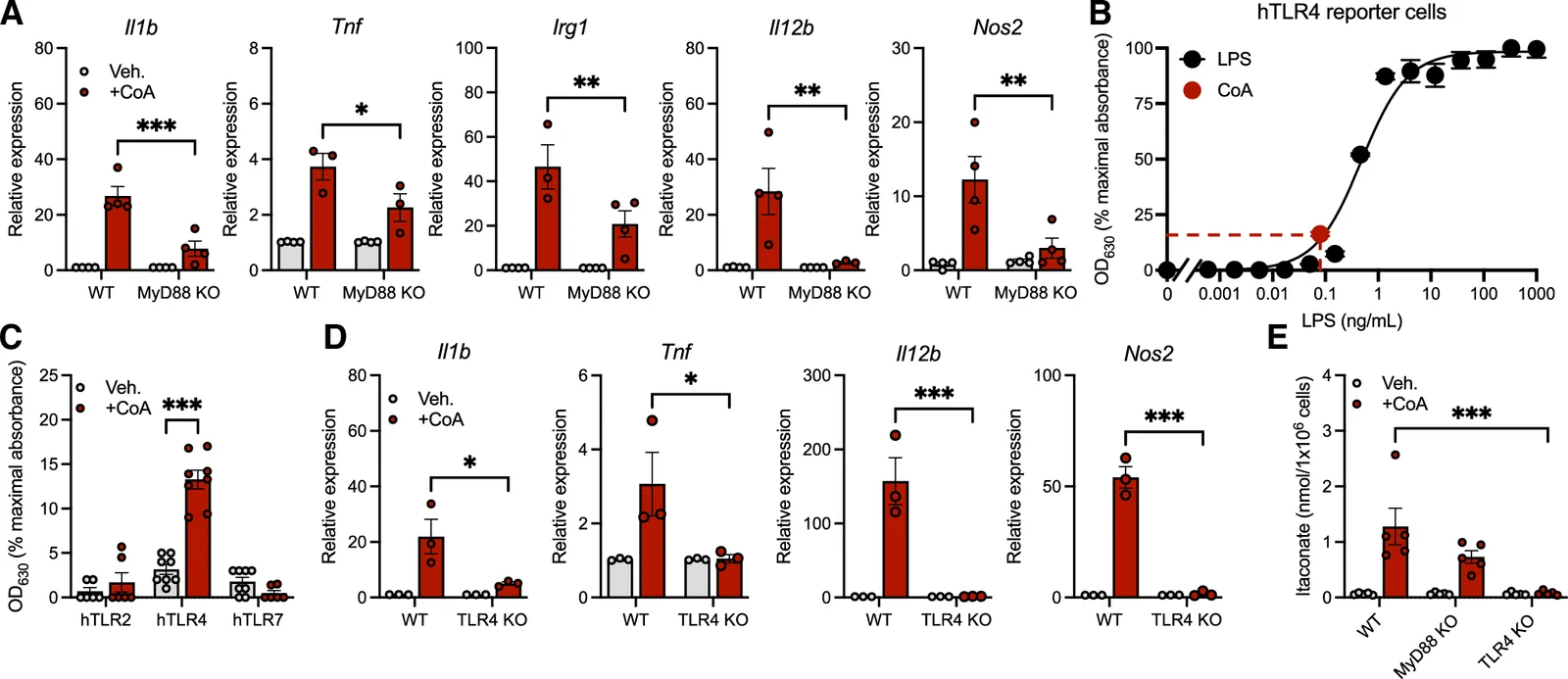
CoA is a TLR4 agonist. (**A**) qPCR analysis of *Il1b*, *Tnf*, *Irg1*, *Il2b*, and *Nos2* in WT and *Myd88*^−/−^ BMDMs stimulated with 1 mM CoA or vehicle control (Veh.) for 4 h (*n* ≥ 3 independent biological replicates) (***P < 0.001, *P = 0.01, **P = 0.010 *Irg1*, **P = 0.005 *Il12b*, **P = 0.005 *Nos2*). (**B**) Concentration-response curve of linked alkaline phosphatase activity in hTLR4 reporter cells with varying concentrations of LPS (black dots). The red dot and dashed lines represent the OD_630_ observed in response to 1 mM CoA treatment (*n* = 4 independent biological replicates). (**C**) Aggregated response of 1 mM CoA compared to vehicle control in hTLR2, hTLR4, and hTLR7 relative to maximum TLR activation (*n* ≥ 3 independent biological replicates) (***P < 0.001). (**D**) qPCR analysis of *Il1b*, *Tnf*, *Il2b*, and *Nos2* in WT and *Tlr4*^−/−^ BMDMs following treatment with 1 mM CoA or vehicle control for 4 h (*n* = 3 independent biological replicates) (*P = 0.01 *Il1b*, *P = 0.02 *Tnf*, ***P < 0.001). (**E**) Abundance of itaconate in WT, *Myd88*^−/−^, and *Tlr4*^−/−^ BMDMs in response to 1 mM CoA treatment for 48 h or vehicle control (*n* ≥ 5 independent biological replicates) (***P < 0.001). All data were presented as mean ± SEM with statistical analysis conducted on biological replicates, each of which included multiple technical replicates, unless otherwise indicated. Response levels in (**C**) that were below the threshold of detection are given as zero. Statistical analysis for (**A**,** C**–**E**) was performed as an ordinary two-way ANOVA followed by Šídák’s post hoc multiple comparisons test. Source data are available online for this figure.

We then hypothesized that CoA could act as an agonist for a specific TLR. Since CoA stimulated the expression of multiple pro-inflammatory genes linked to MyD88 (Fig. 3C)—a signaling adapter protein necessary for full activation of several murine toll-like receptors (Snyder et al, 2013)—we examined whether the pro-inflammatory response from CoA could persist in BMDMs lacking MyD88. BMDMs harvested from *Myd88*^−/−^ mice showed significantly reduced the expression of pro-inflammatory genes induced by CoA, but marginal expression of *Il1b*, *Tnf*, and *Irg1* persisted (Fig. 4A). The result suggested both MyD88-dependent and -independent signaling cascades underlie the pro-inflammatory response elicited by CoA.

We therefore hypothesized that CoA was a TLR4 agonist. Toll-like receptor 4 (TLR4) elicits its inflammatory response by activating both MyD88-dependent and—independent signaling arms (Fitzgerald and Kagan, 2020). The MyD88-dependent pathway of TLR4 causes increased expression of genes encoding cytokines such as *Il1b* (Kawai et al, 1999), while the TRIF pathway is independent of MyD88 and increases expression of ISGs and production of type 1 interferons (Kawai et al, 2001; Toshchakov et al, 2002). Indeed, previous results showed CoA supplementation stimulated expression of the ISG *Mx1* (Fig. 3D).

To determine if CoA is a TLR4 agonist, we utilized a reporter cell line which secretes alkaline phosphatase in response to TLR agonism (Sosa et al, 2021). Addition of CoA activated the human TLR4 (hTLR4) reporter cells (Fig. 4B,C), but no effect was observed in cells expressing hTLR2 or hTLR7, other MyD88-linked TLRs (Figs. 4C and EV3B). Interpolation of a standard curve with LPS, the canonical TLR4 ligand, showed that 1 mM CoA had a comparable effect to 0.1 ng/mL LPS (Fig. 4B) and is a relatively weak TLR4 agonist. BMDMs harvested from *Tlr4*^*−/−*^ mice further confirmed that CoA acts via TLR4, as CoA did not increase expression of pro-inflammatory genes (Fig. 4D) or production of itaconate (Fig. 4E) in TLR4-deficient macrophages.

It was next essential to confirm that the pro-inflammatory response was due to CoA itself rather than an impurity in the ≥85% pure, yeast-derived CoA used in this study. First, we confirmed that the TLR4 activation was not due to endotoxin contamination since the yeast-derived CoA was negative in a Limulus test (Fig. EV3C). In further support of a direct effect of CoA, cells treated with ≥99% pure, synthetically-derived CoA elicited a more potent pro-inflammatory response relative to yeast-derived CoA (Fig. EV3D,E). Although it is unclear why the synthetic CoA triggered a disproportionately strong inflammatory response relative to the CoA of biological origin, the data show that a yeast-derived contaminant cannot explain the pro-inflammatory effect of CoA. Moreover, both the synthetic and yeast-derived CoA elicited the same increase in IL-4-dependent cell surface markers despite the sensitivity difference in TLR4 agonism (Fig. EV3F). In total, the data demonstrate that the CoA molecule itself directly induces an inflammatory response by acting as a weak TLR4 agonist.

**Figure EV4:**
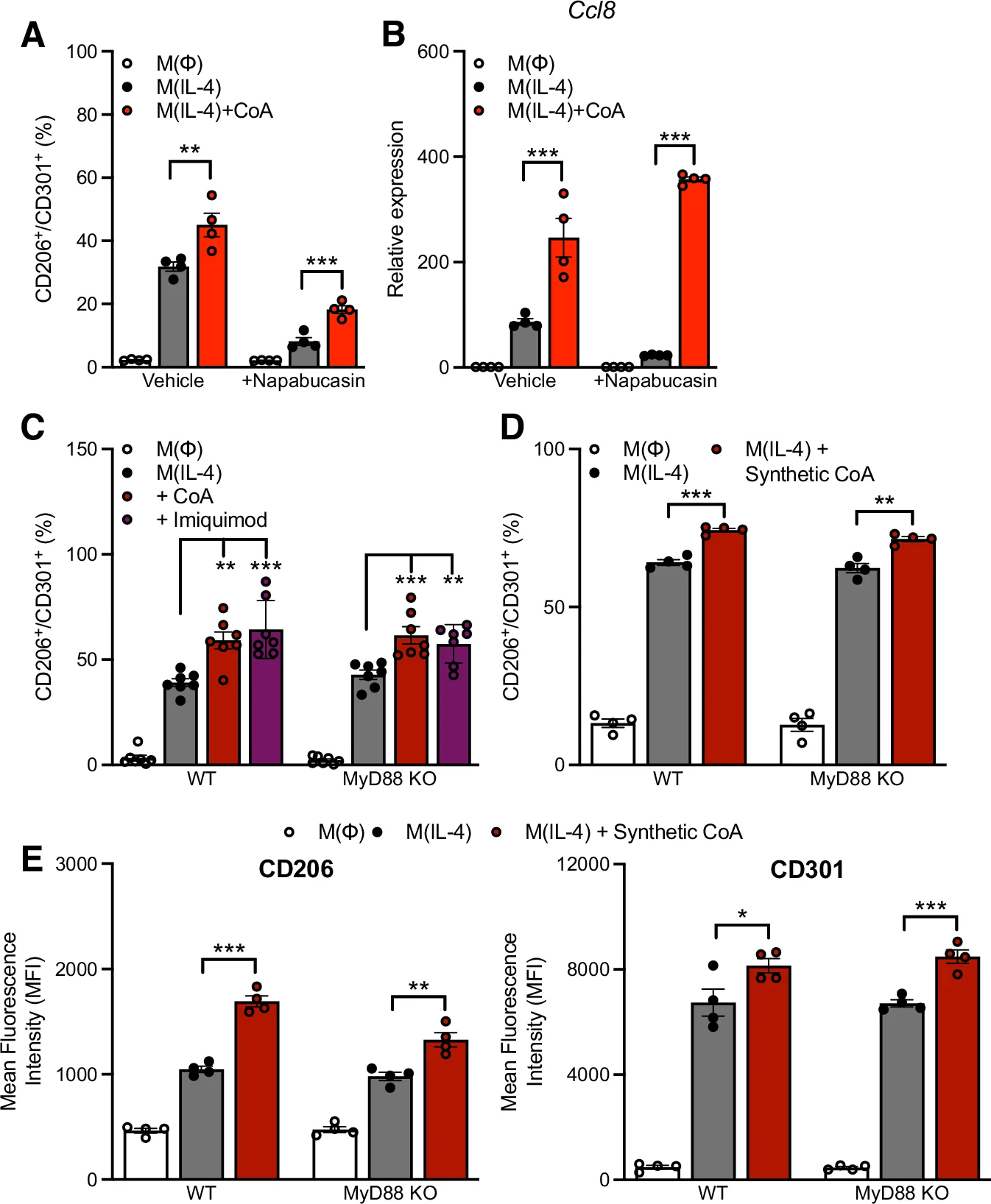
Molecular mechanisms underlying augmentation of M(IL-4) by CoA. (**A**) Percentage of CD206^+^/CD301^+^ population of BMDMs treated with vehicle [M(ϕ)], IL-4 (20 ng/mL), or IL-4 + 1 mM CoA with and without the STAT3 inhibitor Napabucasin (1μM) for 48 h (*n* = 4 independent biological replicates) (**P = 0.005, ***P < 0.001). (**B**) qPCR analysis of *Ccl8* in BMDMs treated as in (**A**) (*n* = 4 independent biological replicates) (***P < 0.001). (**C**) Percentage of CD206^+^/CD301^+^ population of BMDMs harvested from WT and *Myd88*^−/−^ animals upon treatment with vehicle [M(ϕ)], IL-4 (20 ng/mL), IL-4 + yeast-derived CoA (1 mM), or IL-4 + imiquimod (10μM) for 48 h (*n* = 7 independent biological replicates) (**P = 0.001 WT, ***P < 0.001, **P = 0.004 KO). (**D**,** E**) Flow cytometric analysis of BMDMs harvested from WT and *Myd88*^−/−^ animals upon treatment with vehicle [M(ϕ)], IL-4 (20 ng/mL), or IL-4 + synthetic CoA (1 mM) for 48 h. (**D**) Percentage of CD206^+^/CD301^+^ population (***P < 0.001, **P = 0.004) and (**E**) aggregate mean fluorescence intensity of CD206 and CD301 are presented (*n* = 4 independent biological replicates) (CD206: ***P < 0.001, **P = 0.001; CD301: *P = 0.03, ***P < 0.001). All data were presented as mean ± SEM with statistical analysis conducted on biological replicates, each of which included multiple technical replicates, unless otherwise indicated. Statistical analysis for (**A**–**E**) was performed as an ordinary one-way ANOVA followed by Dunnett’s post hoc multiple comparisons test. Source data are available online for this figure.

Given that some pro-inflammatory stimuli can cause STAT3-mediated increases in IL-4 receptor (IL-4Rα) expression (Lang et al, 2002; Mauer et al, 2014), we tested whether CoA enhanced the IL-4 response in a STAT3-dependent manner. Indeed, the well-characterized STAT3 inhibitor napabucasin blunted the overall IL-4 response, reaffirming that STAT3 increases IL-4 responsiveness (Fig. EV4A,B). Nonetheless, CoA provision still augmented the IL-4-induced expression of *Ccl8* and the percentage of CD206^+^/CD301^+^ cells. The results show CoA augments M(IL-4) by a STAT3-independent mechanism, and that the effect cannot be entirely explained by expression-level changes in the IL-4 receptor.

### MyD88-linked TLR agonists enhance the IL-4 response

Next, we asked which signaling cascade downstream of TLR4 was mediating the enhanced macrophage alternative activation. Although previous studies have shown that exposure to LPS and interferon gamma (IFN-γ) inhibits the acquisition of M(IL-4) (Van den Bossche et al, 2016; Khallou-Laschet et al, 2010), much of this work has used high concentrations of LPS that correspond to effects that are orders of magnitude greater than those of CoA (calibrated to 0.1 ng/mL; Fig. 4B) and activate both MyD88 and TRIF.

**Figure 5:**
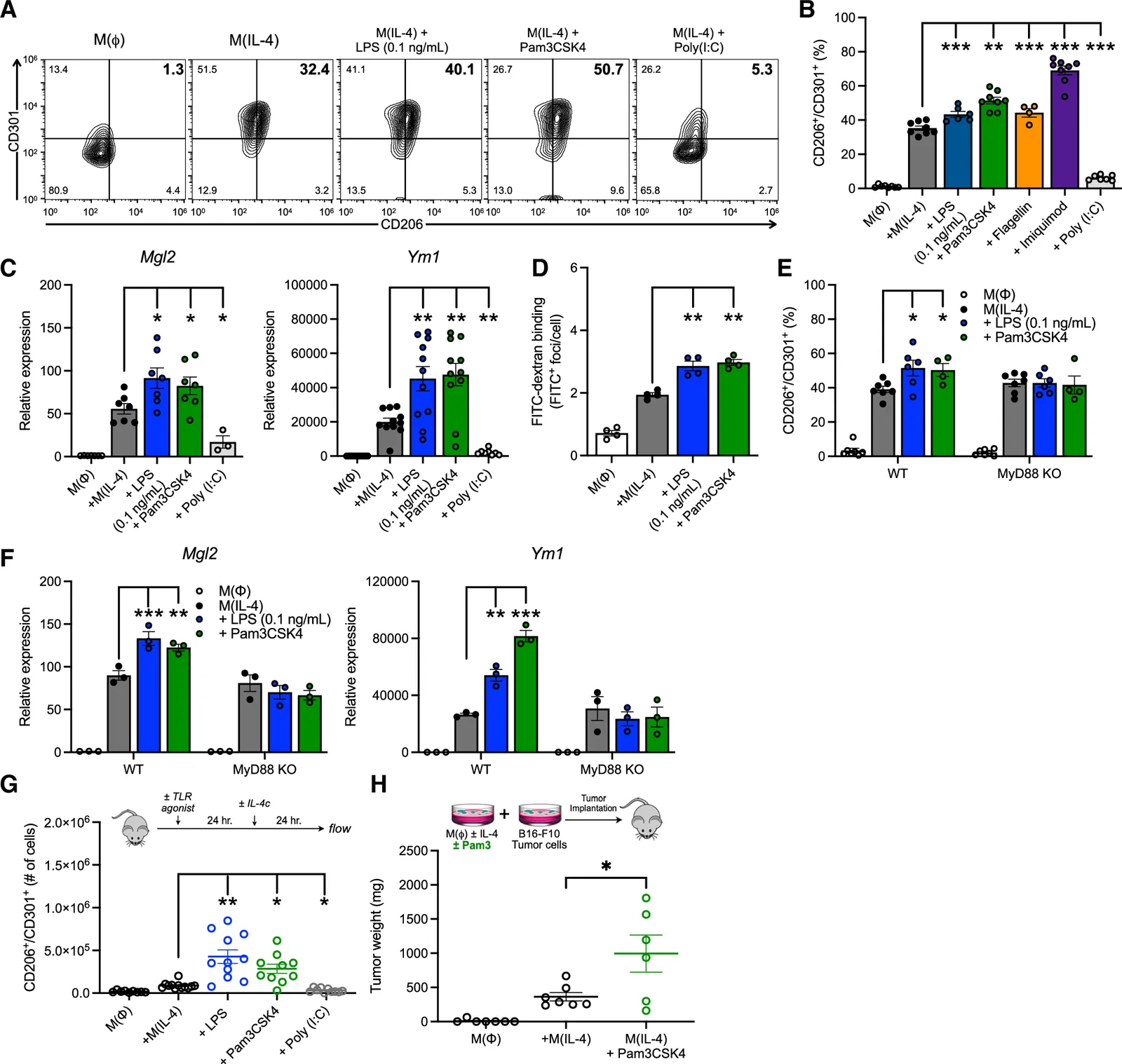
MyD88-linked TLR ligands enhance the IL-4 response. (**A**, **B**) Flow cytometric analysis of CD206 and CD301 in BMDMs stimulated for 48 h (**A**) Contour plots showing the percentage of cells expressing both CD206 and CD301 in the upper right quadrant. BMDMs were stimulated with vehicle control [M(ϕ)], IL-4 (20 ng/mL), or IL-4 in combination with one of the following: LPS (0.1 ng/mL), Pam3CSK4 (5 ng/mL), or Poly (I:C) (1 µg/mL). Data were from a single representative experiment. (**B**) Percentage of CD206^+^/CD301^+^ populations for treatments as in (**A**), as well as IL-4 in combination with imiquimod (10 µM) or flagellin (100 ng/mL) (*n* = 8 independent biological replicates) (**P = 0.002, ***P < 0.001). (**C**) qPCR analysis of *Mgl2* and *Ym1* in BMDMs stimulated with vehicle control, IL-4, or IL-4 in combination with LPS, Pam3CSK4, or Poly (I:C) for 48 h. Concentrations and abbreviations as in (**A**) (*n* ≥ 3 independent biological replicates) (*Mgl2:* *P = 0.01 LPS, *P = 0.02 Pam3CSK4, *P = 0.02 Poly (I:C); *Ym1*: **P = 0.005 LPS, **P = 0.001 Pam3CSK4, **P = 0.001 Poly (I:C)). (**D**) Aggregate FITC^+^ foci per cell for BMDMs stained with FITC-Dextran and treated with vehicle control, IL-4, or IL-4 with either LPS or Pam3CSK4 as in (**A**) (*n* = 4 independent biological replicates) (**P = 0.009 LPS, **P = 0.004 Pam3CSK4). (**E**) Percentage of CD206^+^/CD301^+^ populations in WT and *Myd88*^−/^^−^BMDMs stimulated with vehicle control, IL-4, or IL-4 with either LPS or Pam3CSK4 as in (**A**) (*n* = 8 independent biological replicates) (*P = 0.04 LPS, *P = 0.02 Pam3CSK4). (**F**) qPCR analysis of *Mgl2* and *Ym1* for BMDMs stimulated as in (**E**) (*n* = 4 independent biological replicates) (*Mgl2*: ***P < 0.001, **P = 0.010; *Ym1*: **P = 0.005, ***P < 0.001). (**G**) Quantification of CD206^+^/CD301^+^ peritoneal macrophages from mice that were injected with vehicle control, IL-4c (5 µg IL-4 and 25 µg anti-IL-4 monoclonal antibody), IL-4c + LPS (125 µg), IL-4c +  Pam3CSK4 (50 µg), or IL-4c + Poly (I:C) (200 µg). (*n* ≥ 9 mice were used for each group) (**P = 0.008 LPS, *P = 0.02 Pam3CSK4, *P = 0.03 Poly (I:C)). (**H**) Weights of subcutaneous B16-F10 melanoma tumors derived from the co-implantation of B16-F10 tumor cells and BMDMs that were stimulated with either vehicle control, IL-4, or IL-4 in combination with Pam3CSK4 (*n* = 6–7 mice per group) (*P = 0.01). All data were presented as mean ± SEM with statistical analysis conducted on biological replicates, each of which included multiple technical replicates, unless otherwise indicated. Statistical analysis for (**B**–**E**,** G**, **H**) was performed as an ordinary one-way ANOVA followed by Dunnett’s post hoc multiple comparisons test. Statistical analysis for (**F**) was performed as an ordinary two-way ANOVA followed by Šídák’s post hoc multiple comparisons test. Mixed effects analysis was used for data sets without uniformly matched samples. Source data are available online for this figure.

We therefore activated macrophages with IL-4 and other TLR ligands that activate either MyD88- or TRIF-dependent signaling. Co-treatment with the MyD88-linked TLR2 agonist Pam3CSK4 (Pam3) increased the population of CD206^+^/CD301^+^ BMDMs, whereas this population was decreased upon co-treatment with the TRIF-linked TLR3 agonist Polyinosinic:polycytidylic acid (Poly (I:C)) (Fig. 5A,B). We also stimulated cells with IL-4 in combination with 0.1 ng/mL LPS and observed increased expression of IL-4-dependent cell surface markers (Fig. 5A,B). The TLR5 agonist flagellin and the TLR7 agonist imiquimod, both of which are upstream of MyD88, also increased expression of these IL-4-linked cell surface markers (Fig. 5B).

Indeed, LPS (0.1 ng/mL) and Pam3 also increased expression of IL-4-associated genes (Fig. 5C) and mannose receptor activity (Fig. 5D). As with cell surface marker expression, Poly (I:C) co-treatment lowered the expression of IL-4-stimulated genes (Fig. 5C). To definitively show that MyD88-linked signaling could affect macrophage alternative activation, we examined whether a low concentration of LPS or Pam3 could enhance the IL-4 response in the absence of MyD88. As expected, the effect of LPS and Pam3 on IL-4-associated cell surface markers (Fig. 5E) and genes (Fig. 5F) was lost in BMDMs isolated from *Myd88*^−/−^ mice. Unexpectedly, both CoA (synthetic and yeast-derived) and imiquimod still enhanced the IL-4 response even in the absence of MyD88 (Fig. EV4C–E). However, each of these compounds contains adenine-like moieties, which demonstrably interact with additional plasma membrane receptors that affect the IL-4 response independently of MyD88-linked signaling Csóka et al, 2012.

Lastly, we determined whether MyD88 agonists could improve M(IL-4) in vivo using two independent approaches. First, i.p. injections of either 125 µg LPS or 25 µg Pam3 prior to IL-4 complex increased the number of CD206^+^/CD301^+^ cells harvested from the peritoneal cavity, whereas no difference was observed with Poly (I:C) (Fig. 5G). As further proof-of-concept, we leveraged a tumor model where alternative macrophage activation supports the growth of implanted B16 melanoma tumors (van Teijlingen Bakker et al, 2022; Chen et al, 2017). In line with our previous results, co-treatment of IL-4-stimulated BMDMs with Pam3 resulted in significantly larger tumors when mixed with B16 melanoma cells relative to IL-4 alone (Fig. 5H). In total, these results indicate that activation of the MyD88 pathway enhances alternative macrophage activation in vitro and in vivo.

### MyD88-linked signaling alters the chromatin accessibility of alternatively activated macrophages

**Figure 6:**
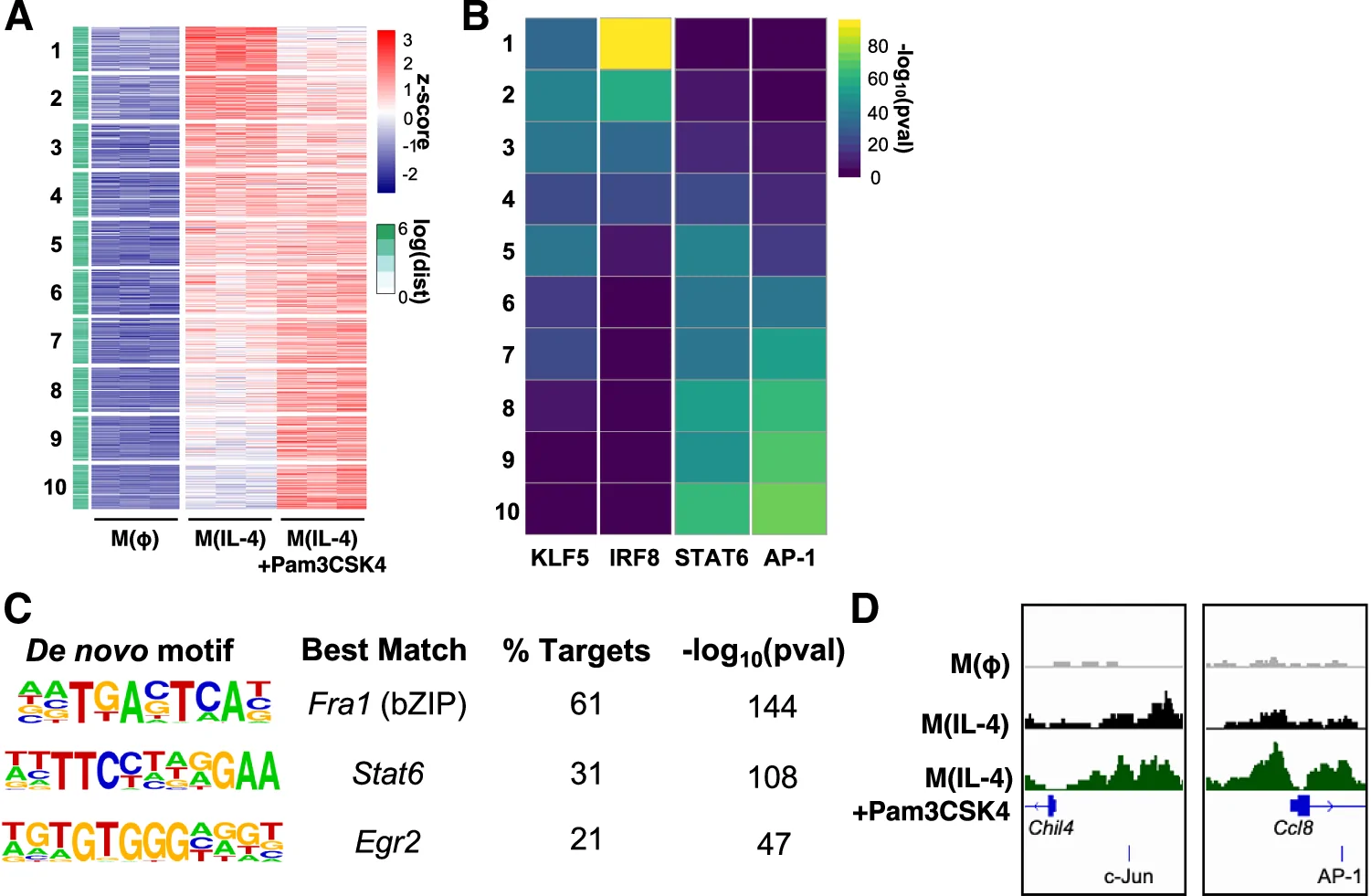
MyD88-linked signaling increases chromatin accessibility in IL-4-stimulated macrophages. (**A**) Heatmap of z-scored ATAC-seq signal for the 10,878 IL-4-inducible regions. The heatmap is arranged by increasing values for IL-4 with Pam3CSK4 co-treatment group and is divided into 10 equal bins. Side bar indicates distance to closest transcription start site (TSS) (*n* = 3 independent biological replicates). (**B**) Heatmap showing *p*-values of the most highly enriched motifs for each of the 10 bins that were generated in (**A**). (**C**) Top three hits from de novo transcription factor motif analysis on the significantly induced regions by Pam3CSK4 co-treatment (log_2_ fold change >0.5, false discovery rate <0.05). (**D**) Representative tracks of Chil4 and Ccl8 promoter regions with nearby c-Jun/AP-1 motifs.

**Figure EV5:**
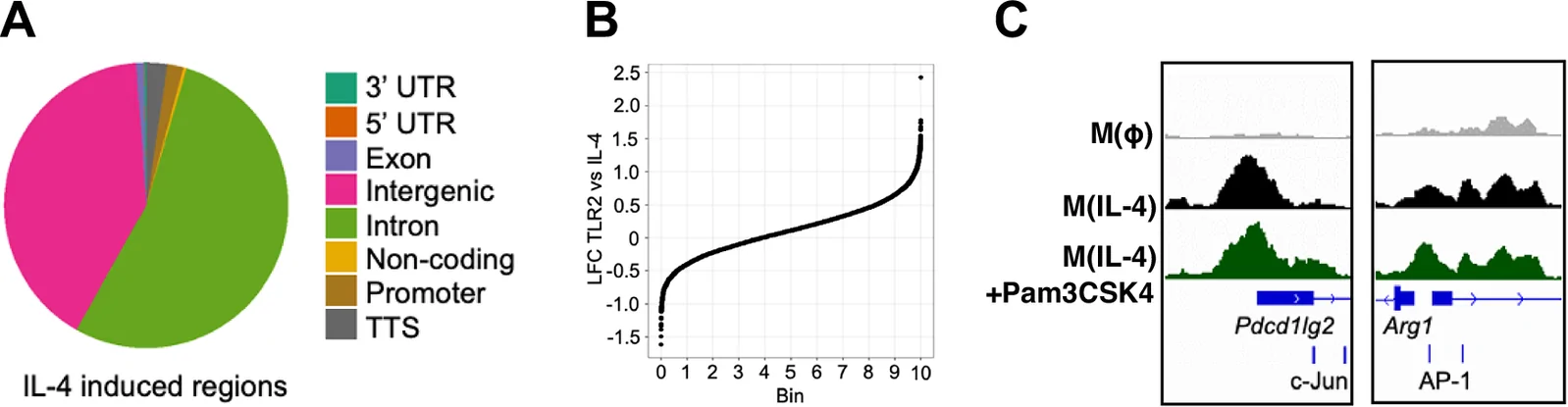
Pam3CSK4 increases the chromatin accessibility of IL-4-induced regions. (**A**) Genomic distribution of the 10,878 IL-4-induced accessibility regions. (**B**) Distribution of Pam3CSK4 (TLR2) and IL-4 co-treatment on the IL-4 inducible regions, divided into ten equal bins. (**C**) Representative tracks of the alternative activation genes *Pdcd1lg2* and *Arg1* promoter regions with nearby c-Jun/AP-1 motifs from BMDMs treated with vehicle [M(ϕ)], IL-4 (20 ng/mL), or IL-4 + 5 ng/mL Pam3CSK4 for 48 h.

Recent studies have highlighted the critical role of epigenetic remodeling when macrophages are exposed to a mix of pro- and anti-inflammatory ligands (Czimmerer et al, 2022; Dichtl et al, 2022). We therefore hypothesized that one mechanism by which MyD88 signaling could augment M(IL-4) was by increasing chromatin accessibility in the promoter regions of IL-4 target genes. To test this, we conducted assay for transposase-accessible chromatin with high-throughput sequencing (ATAC-seq) on IL-4-stimulated BMDMs with or without co-stimulation with Pam3. We first generated a list of the 10,878 genomic regions which had increased accessibility following IL-4 stimulation [log_2_ fold change (LFC) >1, false discovery rate (FDR) <0.01] relative to vehicle controls (Fig. 6A). These regions were localized mainly in intergenic and intronic regions (Fig. EV5A).

We next assessed whether these IL-4-induced regions had increased accessibility upon co-treatment with Pam3 by creating 10 equal bins in increasing order of LFC values (Figs. 6A and EV5B). The analysis revealed over 30% of the IL-4-induced regions were more accessible with Pam3 co-treatment (Bins 8–10; Fig. 6A). To identify potential transcription factors that may mediate the ability of MyD88 to increase alternative activation, we then conducted HOMER transcription factor motif analysis (Heinz et al, 2010). The analysis indicated that these IL-4-induced regions with increased accessibility following Pam3 co-treatment (Bins 8–10) were enriched for STAT6 and Jun/AP-1 binding motifs (Fig. 6B). Moreover, when we expanded our analysis to consider all regions significantly increased by Pam3 (LFC >0.5 and FDR <0.05, *n* = 1766), we noted that these regions were enriched with motifs for the AP-1 subunit *Fra1*, *Stat6*, and *Egr2*. (Fig. 6C). Consistent with our hypothesis, the promoter regions of *Chil4* and *Ccl8*, genes we previously associated with the IL-4 response (Fig. 1A), were more accessible upon exposure to Pam3 (Fig. 6D). Additionally, both had significantly more accessible Jun/AP-1 binding motifs following Pam3 co-treatment. Other genes associated with alternative activation, such as *Pdcd1lg2* and *Arg1*, also had consistent, though not statistically significant, increases in accessibility of Jun/AP-1 binding motifs in their promoter regions following Pam3 co-treatment (Fig. EV5C). In total, ATAC-seq analysis shows that MyD88 activation regulates chromatin accessibility in alternatively activated macrophages. Further, the data identify the Jun/AP-1 family of transcription factors as candidates that may mediate the synergy between the MyD88 pathway and the IL-4 response.

## Discussion

Our results demonstrate that the ubiquitous metabolic cofactor CoA enhances M(IL-4) both in vitro and in vivo. Genetic and pharmacologic proof-of-concept studies show, surprisingly, that CoA is a TLR4 agonist and augments alternative activation via MyD88-linked signaling. This discovery has implications for the metabolic instruction of alternative macrophage activation, the plasticity of macrophage polarization, and the breadth of intracellular metabolites and cofactors that can act as DAMPs.

Unexpectedly, the addition of exogenous CoA did not augment alternative activation by increasing flux through metabolic pathways linked with M(IL-4). In fact, hallmarks of the IL-4 response such as increased respiratory capacity (Puleston et al, 2019; Sanin et al, 2018; Van den Bossche et al, 2016) and de novo lipid synthesis (Bidault et al, 2021) were significantly reduced by CoA provision. Other work using exogenously added prostaglandins shows markers linked to alternative activation can further increase beyond what is induced by IL-4 while simultaneously decreasing mitochondrial oxidative metabolism (Sanin et al, 2018). These results suggest there is flexibility in the metabolic phenotypes that can support alternative macrophage activation, and provide an additional data point to the mixed results regarding whether healthy oxidative phosphorylation is obligatory for M(IL-4) (Divakaruni et al, 2018; Puleston et al, 2019).

Additionally, gain- and loss-of-function experiments with chemical modulators of intracellular CoA levels revealed that altering CoA levels does not adjust alternative macrophage activation. The rationale for examining this hypothesis arose from studying the effects of the CPT-1 inhibitor etomoxir. High, off-target concentrations of the drug block the macrophage IL-4 response and deplete intracellular CoA, and both phenotypes were rescued upon addition of exogenous CoA (Divakaruni et al, 2018). The findings presented here, however, show the link between intracellular CoA levels and alternative macrophage activation is likely associative, and suggest the effects of high concentrations of etomoxir are rescued by MyD88-linked signaling rather than restoration of steady-state CoA levels. Although several independent lines of evidence show that etomoxir blocks the IL-4 response via a mechanism independent of fatty acid oxidation (Divakaruni et al, 2018; Nomura et al, 2016; Ceccarelli et al, 2011), it remains unclear why high concentrations of this lipophilic, reactive epoxide block alternative activation.

Various cellular metabolites, including lipids, ATP, and uric acid, can function as DAMPs to elicit an in vitro pro-inflammatory response (Howell et al, 2011; Wang et al, 2020; Kim et al, 2015). Others, such as the complex lipid prostaglandin E2 and adenosine, can enhance alternative activation (Csóka et al, 2012; Sanin et al, 2018). Here we show that CoA is a putative DAMP that primes macrophages for alternative activation and supports resolution via extrinsic, weak agonism of pro-inflammatory TLR4 and MyD88 signaling.

This aligns with recent reports that show CoA increases the expression of pro-inflammatory genes such as *Il1b*, *Tnf*, and *Nos2* in mouse and human macrophages, an effect lost in mice with simultaneous genetic ablation of TLR2, TLR4, and the TLR chaperone protein Unc93b1 (Timblin et al, 2025). The data presented here that CoA itself is a TLR4 agonist likely explains the effect, rather than CoA having an indirect effect on the pro-inflammatory response via altered mitochondrial metabolism. As CoA consists of an adenosine diphosphate group linked to a phosphopantetheine moiety (Leonardi et al, 2005), it is chemically distinct from many well-characterized TLR4 agonists (Romerio and Peri, 2020). Interestingly, nucleoside analogs such as imiquimod (a nucleoside analog) and CL307 (an adenine analog) are potent TLR7/8 agonists. However, CoA did not activate hTLR7 reporter cells, and additional work is required to understand the structural specificity that enables CoA to activate TLR4.

TLR4 is well characterized for its ability to respond to ligands that are derived from microbes, and its role in sensing endogenous ligands is increasingly appreciated (Rifkin et al, 2005). Release of intracellular proteins, including tenascin-C and high-mobility group box 1 (HMGB1) can induce a TLR4-dependent inflammatory response (Okamura et al, 2001; Midwood et al, 2009; Sosa et al, 2021), but the capacity for intracellular metabolites and metabolic cofactors to activate TLR4 is less established (Lancaster et al, 2018). Here, we show with genetic and pharmacologic proof-of-concept studies that CoA is an endogenous metabolic cofactor that can extrinsically activate TLR4 at physiologically relevant concentrations. The recent development of pantothenate kinase (PANK) double knockout mice provides an exciting model system to genetically manipulate CoA levels in vivo (Subramanian et al, 2020), though myeloid-specific loss of PANK1 and PANK2 is unlikely to show a phenotype, given our results that manipulating intracellular CoA levels in BMDMs does not alter the IL-4 response. Additionally, as CoA plays an essential role in postnatal development (Garcia et al, 2012), it may be difficult to attribute any immunologic phenotypes in genetically modified mice to direct effects of CoA as a metabolic DAMP as opposed to indirect effects from a chronically altered immune system.

Intracellular CoA concentrations can reach over 100 µM in the cytosol and up to 5 mM in the mitochondrial matrix (Williamson and Corkey, 1979; Leonardi et al, 2005; Gout, 2018). As such, cell injury or death could release sufficient CoA to trigger a TLR4-mediated inflammatory response in nearby innate immune cells, especially in poorly vascularized areas where DAMPs can accumulate (Tu and Li, 2023). Our findings may also be particularly relevant to the liver, a tissue where CoA levels are high, and forms of sterile liver injury, such as acetaminophen-induced toxicity, are known to be mediated by DAMPs (Yang and Tonnesseen, 2019). Other intracellular metabolites function as DAMPs following their cell death-induced release (Vénéreau et al, 2015; Cruz et al, 2007), and given that CoA contains an ADP moiety, the findings are broadly consistent with reports that adenosine can enhance the macrophage IL-4 response via the A2 adenosine receptor (Csóka et al, 2012). In fact, the pathway described in our study could inform how other nucleotide or nucleoside analogs (e.g., adenine- or adenosine-containing molecules present at much higher concentrations than CoA) exert their effects on innate immunity. Additionally, our in vitro data with HMDMs raises the exciting possibility that CoA may have a more potent effect on the IL-4 response in humans relative to mice.

Although macrophage polarization is often bifurcated into classical or alternative activation, the physiological and pathological induction of an innate immune response involves heterogeneity in activation signals and functional states (Ruffell et al, 2012; Gordon and Martinez, 2010; Murray et al, 2014). For example, healing processes such as muscle and skin repair are characterized by both an initial influx of pro-inflammatory macrophages to stem infection, as well as a subsequent increase in the presence of alternatively activated macrophages to promote resolution and tissue repair (Hesketh et al, 2017; Arnold et al, 2007). This plasticity can be important for proper function. For example, inhibition of the initial pro-inflammatory response dampens the future expression of alternative activation markers and decreases wound healing (Lucas et al, 2010).

Cooperativity between classical and alternative macrophage activation has been established for more than 20 years, with studies demonstrating that IL-4 can enhance the expression of LPS-induced pro-inflammatory genes (Major et al, 2002). Recent studies have corroborated these findings by demonstrating that IL-4 epigenetically primes macrophages to enhance the transcriptional response to pro-inflammatory stimuli (Czimmerer et al, 2022). Indeed, our work suggests that pro-inflammatory MyD88-linked signaling enhances the IL-4 response, at least in part, by altering chromatin accessibility.

Recent work provides strong support that MyD88-linked signaling via TLR4 agonism is a plausible mechanism by which CoA can augment M(IL-4) (Dang et al, 2022; Rao et al, 2012; Liu et al, 2020; O’Brien and Spiller, 2022). For example, the fungal effector protein Clp1, a TLR4 agonist, fails to enhance alternative activation in MyD88^−/−^ BMDMs. Moreover, other work has associated alternative activation with IL-33 (Faas et al, 2021), which is upstream of MyD88. Others have also shown that interferon-β decreases the expression of cell surface markers and genes associated with alternative activation (Kusnadi et al, 2019; Lee et al, 2022; Dichtl et al, 2022), further supporting our findings that TLR4 agonism by CoA enhances M(IL-4) via MyD88 rather than TRIF. The ATAC-seq analysis presented here furthers this work by identifying Jun/AP-1 signaling as a candidate pathway downstream of MyD88 that may mediate the enhanced IL-4 response. These results, along with our preliminary in vivo work showing MyD88 agonists alter sterile inflammation and melanoma growth, lay the groundwork for future studies examining the mechanistic underpinnings and physiological impact of how metabolites, metabolic cofactors, and drug candidates targeting MyD88 signaling can shape the IL-4 response.

## Methods

**Table:** Reagents and tools table

Reagent/resource	Reference or source	Identifier or catalog number
**Experimental models**		
C57BL/6J (*M.musculus*)	The Jackson Laboratory	Strain# 000664
B6.129P2(SJL)-Myd88<tm1.1Defr>/J (*M.musculus*)	The Jackson Laboratory	Strain# 009088
B6(Cg)-*Tlr4*^*tm1.2Karp*^/J	The Jackson Laboratory	Strain# 029015
Human PBMCs	UCLA CFAR Virology Core	N/A
B16-F10 cells	ATCC	Cat# CRL-6475
HEK-Blue hTLR2 cells	InvivoGen	Cat# hkb-htlr2
HEK-Blue hTLR4 cells	InvivoGen	Cat# hkb-htlr4
HEK-Blue hTLR7 cells	InvivoGen	Cat# hkb-htlr7v2
**Oligonucleotides and other sequence-based reagents**		
*Mgl2* primer: Fwd: AGGCACCCTAAGAGCCATTT Rev: CCTCTTCTCCAGTGTGCTC	This paper	N/A
*Pdcd1lg2* primer: Fwd: GAGCCAGTTTGCAGAAGGTAG Rev: ATCCGACTCAGAGGGTCAATG	This paper	N/A
*Chil4* primer: Fwd: GTGACCCTACTGTTAGTGCTGG Rev: GGTACTTCCTGGGTGGCATCAA	This paper	N/A
*Ccl8* primer: Fwd: TTCCAGCTTTGGCTGTCTCT Rev: GGGTGCTGAAAAGCTACGAG	This paper	N/A
*Fizz1* primer: Fwd: TCGTGGAGAATAAGGTCAAGG Rev: GGAGGCCCATCTGTTCATA	This paper	N/A
*Arg1* primer: Fwd: GCAGAGGTCCAGAAGAATGG Rev: AGCATCCACCCAAATGACAC	This paper	N/A
*Mrc1* primer: Fwd: CAAGGAAGGTTGGCATTTGT Rev: CCTTTCAGTCCTTTGCAAGC	This paper	N/A
*Ym1* primer: Fwd: TAAGACTGGAATTGGTGCCC Rev: CAGACCTCAGTGGCTCCTTC	This paper	N/A
*Il1b* primer: Fwd: GCCCATCCTCTGTGACTCAT Rev: AGGCCACAGGTATTTTGTCG	This paper	N/A
*Tnfa* primer: Fwd: TGCCTATGTCTCAGCCTCTTC Rev: GAGGCCATTTGGGAACTTCT	This paper	N/A
*Nos2* primer: Fwd: CACCTTGGAGTTCACCCAGT Rev: ACCACTCGTACTTGGGATGC	This paper	N/A
*Il12b* primer: Fwd: ATCGTTTTGCTGGTGTCTCC Rev: GGAGTCCAGTCCACCTCTACA	This paper	N/A
*Irg1* primer: Fwd: GCAACATGATGCTCAAGTCTG Rev: TGCTCCTCCGAATGATACCA	This paper	N/A
*Mx1* primer: Fwd: GACCATAGGGGTCTTGACCAA Rev: AGACTTGCTCTTTCTGAAAAGCC	This paper	N/A
*Rplp0* primer: Fwd: CTGTGCCAGCTCAGAACACTG Rev: TGATCAGCCCGAAGGAGAAG	This paper	N/A
*PDCD1GL2* human primer: Fwd: AAAGACCTGTCACCACAACAAAG Rev: AAAGTGCTGGGTCATCCAAAG	This paper	N/A
*TGM2* human primer: Fwd: GAGGAGCTGGTCTTAGAGAGG Rev: CGGTCACGACACTGAAGGTG	This paper	N/A
*CCL17* human primer: Fwd: CCATCGTTTTTGTAACTGTGCAG Rev: TGCATTCTTCACTCTCTTGTTGTTG	This paper	N/A
*IRF4* human primer: Fwd: CTACACCATGACAACGCCTTACC Rev: GGCTGATCCGGGACGTAGT	This paper	N/A
*RPL13A* human primer: Fwd: AAAAAGCGGATGGTGGTTC Rev: CGGTAGTGGATCTTGGCTTT	This paper	N/A
**Chemicals, enzymes and other reagents**		
Napabucasin	Ambeed Inc.	AMBH2D6FF9A0
^**13**^C_5_**-glutamine**	Cambridge Isotope Laboratories	Cat.# CLM-1822
Oligomycin	Sigma-Aldrich	Cat# 75351
Rotenone	Sigma-Aldrich	Cat# R8875
Antimycin A	Sigma-Aldrich	Cat# A8674
FCCP	Sigma-Aldrich	Cat# C2920
Glucose	Sigma-Aldrich	Cat# G8769
Sodium Pyruvate	Sigma-Aldrich	Cat# P5280
L-Glutamine	Sigma-Aldrich	Cat# G3126
HEPES buffer	Sigma-Aldrich	Cat# 83264
DMEM Powder	Sigma-Aldrich	Cat# D5030
Sodium Chloride	Sigma-Aldrich	Cat# S7653
Phenol Red	Sigma-Aldrich	Cat# P0290
HCl	Sigma-Aldrich	Cat# 2104
H_2_SO_4_	Sigma-Aldrich	Cat# 1.60315
Water suitable for cell culture	Sigma-Aldrich	Cat# W3500
RBC lysis buffer	Sigma-Aldrich	Cat# R7757
DMEM high-glucose	Gibco	Cat# 11965
HyClone Characterized FBS	GE	Cat# SH30071.03
Penicillin-streptomycin	Fisher Scientific	Cat# 15-140-122
GlutaMAX	Fisher Scientific	Cat# 35-050-061
Sodium pyruvate (100 mM)	Fisher Scientific	Cat# 11-360-070
M-CSF	Takeshita et al, 2000	N/A
Recombinant human M-CSF	PeproTech	Cat# 300-25
Recombinant mouse IL-4	PeproTech	Cat# 214-14-20UG
Coenzyme A sodium salt hydrate	Sigma	Cat# C4780-10MG
Coenzyme A (free acid)	Avanti Polar Lipids	Cat# 870700P-25mg
Ultrapure LPS-Salmonella Minnesota	InvivoGen	Cat# TLRL-SMLPS
Pam3CSK4	InvivoGen	Cat# TLRL-PMS
Poly (I:C) HMW	InvivoGen	Cat# TLRL-PIC
Imiquimod	InvivoGen	Cat# TLRL-IMQ
FLA-ST Ultrapure (Flagellin)	InvivoGen	Cat# TLRL-EPSTFLA
CL307	InvivoGen	Cat# TLRL-C307
IL-4 complex	BioXCell	Cat# BE0045
Cyclopropane carboxylic acid	Sigma	Cat# C116602
PZ-2891	MedChemExpress	Cat# HY-124634
Dulbecco’s phosphate-buffered saline (DPBS)	Fisher Scientific	Cat# 14-190-250
16% Paraformaldehyde	Fisher Scientific	Cat# 50-980-487
Hoechst 33342	Fisher Scientific	Cat# H3570
Methanol	Sigma-Aldrich	Cat# 34860-4L-R
Chloroform	Sigma-Aldrich	Cat# 366927
Norvaline	Sigma-Aldrich	Cat# N7502
Methoxyamine hydrochloride	Sigma-Aldrich	Cat# 226904
Pyridine	Sigma-Aldrich	Cat# 270407
*N*-tert-Butyldimethylsilyl-*N*-methyl-trifluoroacetamide w/1% tert-Butyldimethylchlorosilane	Sigma-Aldrich	Cat# 00942
^13^C_6_-glucose	Cambridge Isotope Laboratories	Cat# CLM-1396-5
Accutase	Innovative Cell Technologies	Cat# AT-104-500 ml
EDTA	Sigma-Aldrich	Cat# 03690
TruStain FCX	BioLegend	Cat# 101320
Mouse CD206	BioLegend	Cat# 141710
Mouse CD301	Bio-Rad	Cat# MCA2392A647T
Mouse CD71	BioLegend	Cat# 113812
DAPI	Invitrogen	Cat# D1306
FITC-Dextran	Sigma-Aldrich	Cat# FD40
5-sulfosalicylic acid	Sigma-Aldrich	Cat# 247006
Crotonoyl CoA	Sigma-Aldrich	Cat# C6146
HEK-blue detection medium	InvivoGen	Cat# hb-det2
Tn5 transposase	Illumina	Cat# 20034197
NEBNext High-Fidelity PCR Mix	New England Biolabs	Cat# M0541S
**Software**		
Agilent Seahorse XF Wave	Agilent	https://www.agilent.com/en/product/cell-analysis/real-time-cell-metabolic-analysis/xf-software/seahorse-wave-pro-software-2007523
Agilent GCMS MassHunter	Agilent	N/A
Attune NxT Software v3.1.2	Thermo Fisher	N/A
FlowJo X	FlowJo	N/A
Harmony	PerkinElmer	N/A
Illumina Bcl2fastq v2.19.1	Illumina	N/A
GraphPad Prism	GraphPad	https://www.graphpad.com/
Excel	Microsoft	https://www.microsoft.com/en-us/microsoft-365/excel
**Other**		
Seahorse XFe96 Analyzer	Agilent	https://www.agilent.com/en/product/cell-analysis/real-time-cell-metabolic-analysis/xf-analyzers/seahorse-xfe96-analyzer-740879
Seahorse XFe96 FluxPak	Agilent	102416-100
Operetta CLS High-Content Analysis System	PerkinElmer	N/A
Multimode plate reader	Tecan	N/A
CentriVap vacuum concentrator	LabConco	N/A
Attune NxT Flow Cytometer	ThermoFisher	N/A
RNeasy Mini Kit	Qiagen	Cat# 74106
High-capacity cDNA reverse transcription kit	Applied Biosystems	Cat# 4368814
PowerUP SYBR Green qPCR Master Mix	Applied Biosystems	Cat# A25743
QuantStudio 5	Applied Biosystems	N/A
LEGENDplex Mouse M1 Macrophage Panel (8-plex) with V-bottom plate	BioLegend	Cat# 740848
DNA Clean & Concentrator Kit	Zymo	DNA Clean & Concentrator Kit
Kapa Library Quant Kit	Roche	Cat#07960140001
4200 TapeStation System	Agilent	Cat# G2991BA
HiSeq 4000 platform	Illumina	N/A
KAPA stranded mRNA-Seq kit	Roche	Cat#07962193001
NovaSeq 6000	Illumina	N/A

### Animals

All animal housing and experimental procedures were approved by the UCLA Animal Research Committee (ARC) under protocol ARC 2020-027 and conducted in accordance with the NIH Guide for the Care and Use of Laboratory Animals. Mice were group housed (four per cage) and maintained under a standard 12-h light-dark cycle (lights on 7:00-19:00) in a humidity-controlled colony room. Animals were given ad libitum access to water and fed a standard chow diet. Animals were given environmental enrichments, and UCLA veterinary staff provided weekly assessments of general animal health.

### Reagents

Unless otherwise specified, yeast-derived CoA purchased from Sigma-Aldrich (C4780; ≥85% purity) was used. Synthetic CoA was purchased from Avanti Polar Lipids (870700P; >99% purity). IL-4 was purchased from PeproTech (214-14), and IL-4-reactive monoclonal antibodies for in vivo studies (IL-4 MAb) were purchased from BioXCell (BE0045). All TLR agonists were purchased from Invivogen: Pam3CSK4 (TLR2 ligand; tlrl-pms), Poly(I:C) (TLR3 ligand; tlrl-pic), LPS (TLR4 ligand; tlrl-smlps), Flagellin (TLR5 ligand; tlrl-stfla), and imiquimod (TLR7 agonist; tlrl-imqs-1).

### Isolation and differentiation of mouse BMDMs

BMDMs from wild-type (000664), *Myd88*^−/−^ (009088), and *Tlr4*^−/−^ (029015) mice were isolated as previously described from male mice aged between 6 and 12 weeks (Hsieh et al, 2021). Bone marrow cells were first isolated by flushing the femurs and tibiae with phosphate-buffered saline (PBS). Cells were then pelleted by centrifugation at 365 × *g* for 7 min at room temperature. Following the removal of red blood cells with RBC Lysis Buffer (Sigma, R7757) and centrifugation, bone marrow cells were differentiated for 6 days at 37 °C in a humidified 5% CO_2_ incubator in “BMDM differentiation medium”. BMDM differentiation medium consisted of DMEM (Gibco #11965) supplemented with 10% (v/v) fetal bovine serum (FBS; Hyclone), 2 mM L-glutamine, 500 µM sodium pyruvate, 100 units/mL penicillin, and 100 mg/mL streptomycin. Additionally, the medium was further supplemented 10% (v/v) conditioned medium from CMG-14-12 cells as a source of macrophage colony-stimulating factor (M-CSF) (Takeshita et al, 2000).

### Isolation of human PBMC-derived macrophages

Single-donor human peripheral blood mononuclear cells (PBMCs) were obtained for monocyte isolation from the UCLA CFAR Virology Core in line with pre-established methods (Ball et al, 2025; Zhou et al, 2020). Monocytes were harvested by standard Ficoll isolation procedures and enriched with plastic adherence. For macrophage differentiation, 1 × 10^7^ monocytes [suspended in DMEM supplemented with 10% (v/v) FBS with 2 mM L-glutamine, 100 units/mL penicillin, 100 µg/mL streptomycin, 500 µM sodium pyruvate, and 50 ng/mL of recombinant human M-CSF (Peprotech, 300-25)] were cultured on Petri Dishes (Fisher #FB0875712) for 6 days prior to assays. The medium was changed 4 days after the initial differentiation.

### In vitro BMDM activation and stimulation of Toll-like receptors

After 6 days of differentiation, BMDMs were scraped with a cell lifter, counted, and replated at the listed cell densities into the relevant assay format (e.g., six-well tissue culture dish, Seahorse XF96-well plate, etc.) in differentiation medium. After 2 days, cells were stimulated with compounds as indicated below and in the figure legends. Unless otherwise specified, all measurements of the macrophage IL-4 response were conducted 48 h after treatment. When assessing the effect of CoA on the pro-inflammatory response, BMDMs were treated for 4 h (gene expression) or 48 h (itaconate abundance). Unless otherwise indicated in the figures and legends, effector compounds were used at the following concentrations alongside matched vehicle controls: IL-4 (20 ng/mL), CoA (1 mM), Pam3CSK4 (5 ng/mL), Poly (I:C) (1 µg/mL), LPS (0.1 ng/mL), flagellin (100 ng/mL), and imiquimod (10 µM).

### In vivo activation of peritoneal macrophages

To induce alternative activation in vivo, mice were treated with an IL-4 complex (IL-4c) consisting of a 2:1 molar ratio of IL-4 and anti-IL-4 mAb or PBS as a control. Each IL-4c-treated mouse received 5 μg IL-4 and 25 μg of anti-IL-4 mAb. To test the effect of CoA on alternative activation in vivo (Fig. 1G), mice were pretreated with an i.p. injection of either PBS or 40 mg/kg CoA for 6 h prior to IL-4c administration according to the scheme in the figure. After 24 h, peritoneal macrophages were collected by rinsing the peritoneal cavity with 5 mL PBS, and alternative activation markers were assessed using flow cytometry.

To determine if CoA could induce a pro-inflammatory response in vivo (Fig. 3F,G), mice were treated with either PBS or 40 mg/kg CoA for 6 h. After treatment, peritoneal macrophages were collected by rinsing the peritoneal cavity with 5 mL of PBS. Cells were then pelleted, with supernatant used to measure cytokine levels with the LEGENDplex multiplex ELISA kit (BioLegend, 740848), while gene expression was measured in the peritoneal exudate cells.

To determine if TLR agonists could enhance the IL-4 response of peritoneal macrophages in vivo (Fig. 5G), Mice were treated with either PBS or the indicated TLR agonist at the following doses: Pam3CSK4 (50 μg), Poly:IC (200 μg), or LPS (125 μg). After 24 h, IL-4c was administered as before, and the number of cells double-positive for CD206 and CD301 was assessed by flow cytometry. Researchers were not kept blind to the treatment groups.

### B16 melanoma growth

Prior to in vivo co-injection, in vitro BMDMs were either stimulated with vehicle control, IL-4, or IL-4 in combination with Pam3CSK4 for 48 h. On the day of implantation, a 1:1 mixture of 1 × 10^5^ B16-F10 cells and 1 × 10^5^ BMDMs were suspended in PBS and injected into the rear right flanks of 12-week-old male C57BL/6 mice tumors (van Teijlingen Bakker et al, 2022). Mice were sacrificed 20 days post injection and subcutaneous tumors were excised, blotted dry, and weighed. Researchers were not kept blind to the treatment groups.

### Gene expression analysis

Gene transcript levels were measured using qPCR. On day 6 of differentiation, BMDMs were seeded at 3.0 × 10^5^ cells/well in 12-well plates. After activating cells with the concentrations and durations of stimuli as described earlier, RNA was extracted using the RNeasy Mini Kit (Qiagen, 74106) and cDNA was synthesized using the high-capacity cDNA reverse transcription kit (Applied Biosystems, 4368814) according to the manufacturers’ protocol. qPCR was performed with PowerUp SYBR green master mix (Applied Biosystems, A25743) on a QuantStudio 5 RT-PCR (Applied Biosystems). Relative gene expression was calculated using the ΔΔC_t_ method, with *Rplp0* (mouse) or *RPL13A* (human) used as controls for normalization.

### Flow cytometric analysis

Cell surface marker expression was measured using flow cytometry. On day 6 of differentiation, BMDMs were seeded at 3.0 × 10^5^ cells/well in 12-well plates. After activating cells with the concentrations and durations of stimuli as described earlier, BMDMs were detached by scraping in 450 µL of Accutase. Cells were then washed with FACS buffer (PBS + 2% (v/v) FBS with 1 mM EDTA) and incubated with a 1:500 dilution of TruStain FCX (BioLegend, 101320) for 5 min. Cells were next stained for 30 min on ice with a 1:300 dilution of antibodies raised against mouse CD206 (BioLegend,141710), CD301 (Bio-Rad, MCA2392A647T), or CD71 (BioLegend, 113812). Cells were then washed and resuspended in FACS buffer containing 1 μg/mL DAPI (Invitrogen, D1306) for 5 min for viability analysis. Following a final wash, cells were resuspended in FACS buffer, and data were captured on an Attune NxT flow cytometer. Flow cytometry gating was based on the exclusion of DAPI⁺ cells to identify viable populations, with isotype controls included for each antibody to define positive gates. Data were analyzed using FlowJo X software.

### Endocytosis assay

Following differentiation, BMDMs were seeded in black-walled 96-well plates at 3 × 10^4^ cells/well for high-content imaging to quantify uptake of FITC-dextran. 48 h after compound treatment, medium was replaced with high-glucose DMEM containing 1 mg/mL FITC-dextran (Sigma, FD40) and 10 ng/mL Hoechst 33342. Following a 1 h incubation at 37 °C, cells were washed twice with PBS and fixed with 4% (v/v) paraformaldehyde (PFA) in PBS. Images were captured with a PerkinElmer Operetta, and FITC-positive foci per cell was calculated using Harmony software.

### Quantification of short-chain acyl CoAs

Following differentiation, BMDMs were seeded in 10 cm^2^ dishes at 5 × 10^6^ cells/dish. Following 48 h stimulation, cells were rinsed twice with ice-cold PBS, scraped into 1.5 mL microcentrifuge tubes and pelleted via centrifugation at 4 °C. 200 μL of an ice-cold extraction solution [2.5% (w/v) 5-sulfosalicylic acid along with 1 μM crotonoyl CoA as an internal standard] was added to each cell pellet and subsequently vortexed. Samples were centrifuged at 18,000 x *g* for 15 min at 4 °C. Supernatants containing short-chain acyl CoAs were then removed and transferred to glass LC-MS vials for analysis as described elsewhere (Jones et al, 2021).

### Seahorse XF analysis

After 6 days of differentiation, BMDMs were plated at 3.0 × 10^4^ cells/well in XF96 plates. Following 48 h of treatment with compounds, respirometry assays were conducted with an Agilent Seahorse XFe96 Analyzer. Oligomycin (2 μM), two injections of FCCP (750 nM each), and rotenone (200 nM) with antimycin A (1 μM) were added acutely to the wells, and respiratory parameters were calculated according to best practices (Divakaruni and Jastroch, 2022; Divakaruni et al, 2014). Measurements were conducted in unbuffered DMEM (Sigma #5030) supplemented with 5 mM HEPES, 8 mM glucose, 2 mM glutamine, and 2 mM pyruvate. Lactate efflux was measured by correcting rates of extracellular acidification for microplate sensor coverage and confounding respiratory acidification (Desousa et al, 2023).

### Metabolomics and stable isotope tracing of polar metabolites

After 6 days of differentiation, BMDMs were plated at 1 × 10^6^ cells/well in six-well dishes. Cells were then stimulated in medium where either glucose or glutamine was replaced with uniformly labeled ^13^C_6_-glucose (CLM-1396) or uniformly labeled ^13^C_5_-glutamine (CLM-1822). After 48 h, cells were harvested and extracted for GC/MS using established methods, with all steps conducted on ice (Cordes and Metallo, 2019). Briefly, cell plates were washed twice with 0.9% (w/v) NaCl and samples were extracted with 500 μL methanol, 200 μL water containing 1 μg of norvaline (internal standard), and 500 μL chloroform. Samples were vortexed for 1 min and spun at 10,000 × *g* for 5 min at 4 °C, and the aqueous layers containing the polar metabolites were transferred to GC/MS sample vials and dried overnight using a refrigerated CentriVap. Once dry, the samples were resuspended in 20 μL of 2% (w/v) methoxyamine in pyridine and incubated at 37 °C for 45 min. This was followed by the addition of 20 μL of MTBSTFA + 1% TBDMS (*N*-tert-butyldimethylsilyl-*N*-methyl-trifluoroacetamide with 1% tert-butyldimethylchlorosilane). Following a second 45-min incubation at 37 °C, samples were run as previously described (Cordes and Metallo, 2019). Analysis was conducted using Agilent MassHunter software, and stable isotope tracing data were corrected for natural abundance of heavy isotopes with FluxFix software using a reference set of unlabeled metabolite standards (Trefely et al, 2016).

### De novo lipogenesis

Briefly, after 6 days of differentiation, BMDMs were plated at 1 × 10^5^ cells/well in 24-well dishes. Cells were stimulated in medium in which unlabeled glucose was replaced with 10 mM uniformly labeled ^13^C_6_-glucose. Extraction of fatty acids, quantification of de novo synthesis, and normalization to cell number was conducted using an Agilent 5975C mass spectrometer coupled to a 7890 gas chromatograph as previously described (Argus et al, 2018; Hsieh et al, 2021).

### Cell counts and normalization

When normalizing plate-based assays to cell number for absolute quantification (e.g., Seahorse XF respirometry or metabolite analysis), BMDMs were fixed immediately upon assay completion with 2% (w/v) formaldehyde for 20 min at room temperature. Plates were subsequently refrigerated for 1–14 days until assessment. Nuclei were stained with Hoechst (Thermo Fisher #33342) at 10 ng/mL overnight at room temperature, and cell counts were obtained by automated high-content imaging using the PerkinElmer Operetta in accordance with established methods (Ball et al, 2025; Divakaruni et al, 2018).

### HEK-Blue hTLR reporter assays

HEK-Blue reporter cells expressing either human TLR2 (hkb-htlr2), hTLR4 (hkb-htlr4), or hTLR7 (hkb-htlr7v2) were purchased from InvivoGen, maintained according to the manufacturer’s instructions, used at low passage (*p* < 6), and confirmed to be free of mycoplasma. To establish concentration-response curves, 2.5 × 10^3^ reporter cells were resuspended in HEK-Blue detection medium (Invivogen, hb-det2) in 96-well plates and stimulated with the appropriate agonist (Pam3CSK4: 0.6 pg/mL to 1 µg/mL; LPS: 0.6 pg/mL to 1 µg/mL; CL307: 0.6 pg/mL to 1 µg/mL). Following a 24 h incubation, secreted alkaline phosphatase reporter activity was determined by assessing OD_630_ with a plate reader. The OD_630_ of cells treated with 1 mM CoA was compared relative to positive controls. Calibration curves to fit empirical data were generated using GraphPad Prism software.

### ATAC-seq library prep

ATAC-seq libraries were prepared by the Applied Genomics, Computation and Translational Core Facility at Cedars-Sinai using previously described protocols (Cheng et al, 2023; Buenrostro et al, 2015). Briefly, 5 × 10^4^ BMDMs per sample were lysed to collect nuclei and treated with Tn5 transposase (Illumina) for 30 min at 37 °C with gentle agitation. The DNA was isolated with the DNA Clean & Concentrator Kit (Zymo) and PCR amplified and barcoded with NEBNext High-Fidelity PCR Mix (New England Biolabs) and unique dual indexes (Illumina). The ATAC-seq library amplification was confirmed by real-time PCR, and additional barcoding PCR cycles were added as necessary while avoiding overamplification. Amplified ATAC-seq libraries were purified with the DNA Clean & Concentrator Kit (Zymo). The purified libraries were quantified with the Kapa Library Quant Kit (KAPA Biosystems) and quality assessed on a 4200 TapeStation System (Agilent). The libraries were pooled based on molar concentrations and sequenced on a HiSeq 4000 platform (single end, 75 bp).

### ATAC-seq analysis

The peaks for all the ATAC-seq samples were used to generate a single reference peak file, and the number of reads that fell into each peak was counted using deeptools multiBamSummary (Ramírez et al, 2016). EdgeR (Robinson et al, 2009) was used to determine the IL-4 significantly induced regions by applying a cutoff FDR <0.01 and LFC >1 of triplicate data upon IL-4 stimulation on WT BMDMs and to determine the Pam3CSK4 co-treatment significant regions by applying a cutoff FDR <0.05 and LFC >0.5. Analysis of transcription factor motif enrichment was performed using the findMotifsGenome function in the HOMER suite (Heinz et al, 2010), using all detected peaks as background. Reads were normalized by RPKM. Data were visualized with ggplot2 or the pheatmap packages in R.

### RNA-Seq library prep and quantification

RNA sequencing libraries were produced by the Technology Center for Genomics & Bioinformatics at UCLA. Isolation of RNA was performed using the Qiagen RNeasy Mini kit and RNA libraries were prepared with the KAPA stranded mRNA-Seq kit. High-throughput sequencing was performed on Illumina NovaSeq 6000 (paired end, 2 × 150bp) targeting 100 million reads per sample. Demultiplexing was performed with Illumina Bcl2fastq v2.19.1. Gene expression quantification from the resulting fastqs was performed using Salmon v1.2.1 in mapping-based mode. Reads were selectively aligned to the GENCODE vM25 mouse reference transcriptome with corrections for sequence-specific and GC content biases.

### RNA-seq analysis

Raw gene count data were analyzed using the R package DESeq2 v1.22.2 (Love et al, 2014) for library size normalization and differential expression analysis. For differential expression results, genes with adjusted *p* values below 0.01 and log2 fold changes above 1 or below −1 were deemed significant. For visualization in volcano plots, log2 fold changes above 10 or below −10 were set to 10 and −10, respectively. Gene set enrichment analysis (Subramanian et al, 2005) was performed using the R package FGSEA v1.15.0 (Korotkevich et al, 2019) based on gene lists ranked by the Wald statistic from differential expression results. Genesets corresponding to the mouse transcriptome from KEGG, REACTOME, and BIOCARTA within the Molecular Signatures Database (Liberzon et al, 2015) were accessed using the R package msigdbr v7.1.1. Genesets with adjusted *p* values below 0.05 were deemed significant.

### Statistical analysis

All statistical parameters, including the number of biological replicates (*n*), can be found in the figure legends. Statistical analyses were performed using GraphPad Prism 11 software. Data were presented as the mean ± standard error of the mean unless otherwise specified. Individual pairwise comparisons were performed using a two-tailed Student’s *t*-test. For analysis involving more than two groups, data were analyzed by ANOVA followed by Tukey’s, Sídák’s, or Dunnett’s post hoc multiple comparisons tests (compared against vehicle controls unless otherwise specified). Data were assumed to follow a normal distribution (no tests were performed). Values denoted as follows were considered statistically significant: *p < 0.05; **p < 0.01; ***p < 0.001.

### Graphics

The synopsis image was created with BioRender.com.

## Supplementary information

Peer Review File

Source data Fig. 1

Source data Fig. 2

Source data Fig. 3

Source data Fig. 4

Source data Fig. 5

Figure EV1 Source Data

Figure EV2 Source Data

Figure EV3 Source Data

Figure EV4 Source Data

Expanded View Figures

## Data Availability

The data sets produced in this study are available in the following databases: RNA-seq data: Gene Expression Omnibus GSE324239 (https://www.ncbi.nlm.nih.gov/geo/query/acc.cgi?acc=GSE324239). ATAC-seq data: Gene Expression Omnibus GSE324240 (https://www.ncbi.nlm.nih.gov/geo/query/acc.cgi?acc=GSE324240). The source data of this paper are collected in the following database record: biostudies:S-SCDT-10_1038-S44319-026-00925-y.
