# Supplementary material for: Coenzyme A activates TLR4 and enhances alternative macrophage activation in line with MyD88 agonism
Source: EMBO Rep. 2026 Sep 18;27(20):5927–52. doi: 10.1038/s44319-026-00925-y (PMC13694274; doi:10.1038/s44319-026-00925-y)
Supplement: Supplementary file 1 [file 44319_2026_925_MOESM11_ESM.pdf]

Expanded View Figures

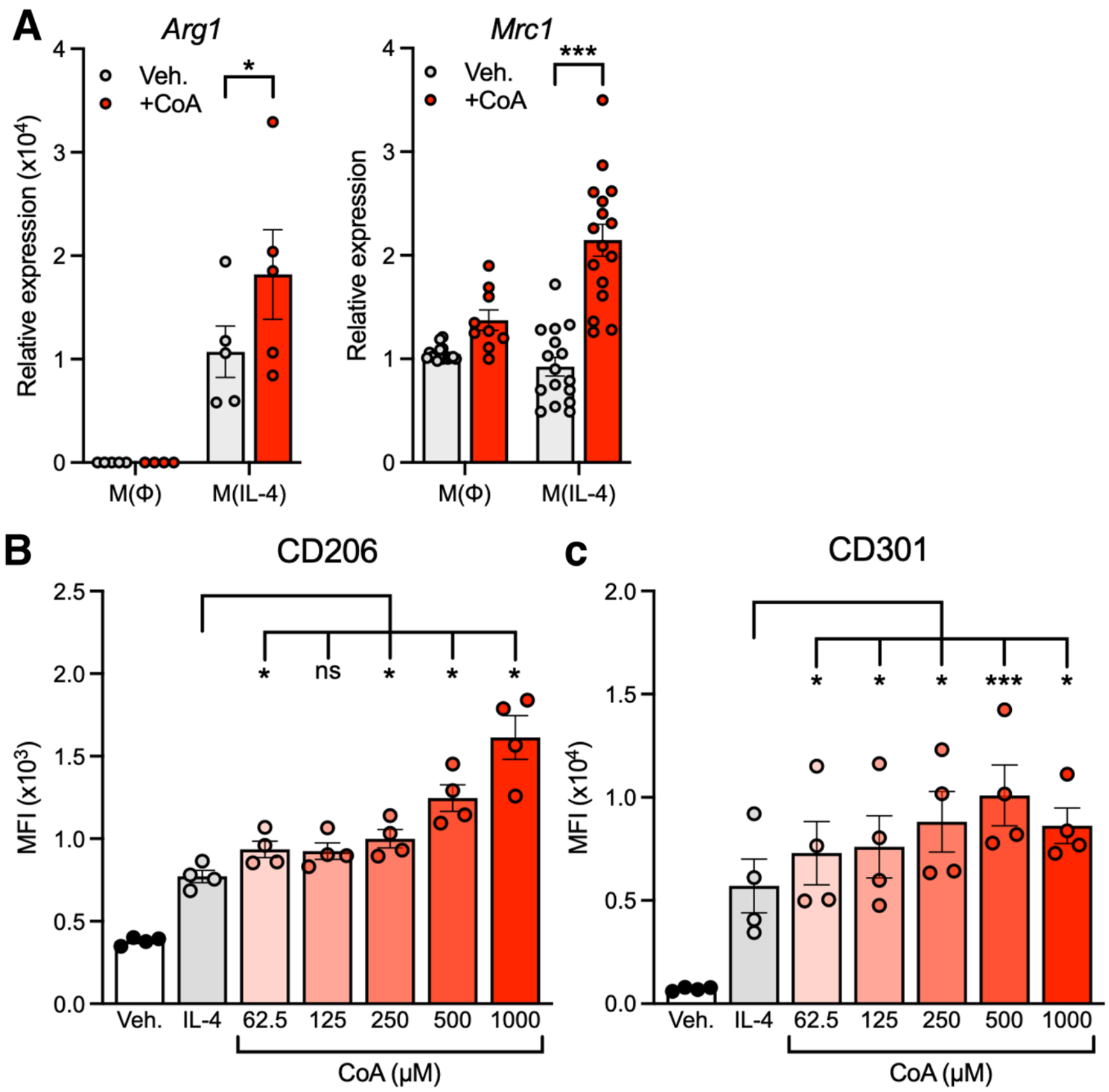

Figure EV1. Exogenous CoA provision enhances alternative macrophage activation.

(**A**) qPCR analysis of the IL-4-associated genes *Arg1 and Mrc1* in BMDMs treated with vehicle (Veh.), CoA (1 mM), IL-4 (20 ng/mL), or CoA + IL-4 for 48 h. ($n \geq 9$ independent biological replicates) (*$P = 0.02$, ***$P < 0.001$). (**B**, **C**) Mean fluorescence intensity of (**B**) CD206 and (**C**) CD301 in BMDMs following treatment with vehicle, IL-4 (20 ng/mL), or IL-4 + CoA with concentrations as indicated in the figure for 48 h. ($n = 4$ independent biological replicates) (CD206: *$P = 0.04$ 62.5 μM, *$P = 0.02$ 250 μM, *$P = 0.01$ 500 μM, *$P = 0.02$ 1000 μM; CD301: *$P = 0.03$ 62.5 μM, *$P = 0.01$ 125 μM, *$P = 0.01$ 250 μM, ***$P < 0.001$, *$P = 0.03$ 1000 μM). All data were presented as mean ± SEM with statistical analysis conducted on biological replicates, each of which included multiple technical replicates, unless otherwise indicated. Statistical analysis for (**A**) was performed as a paired, two-tailed *t*-test. Statistical analysis for (**B**, **C**) was performed as a one-way ANOVA followed by Dunnett's post hoc multiple comparisons test. ns no significance. Source data are available online for this figure.

© The Author(s)

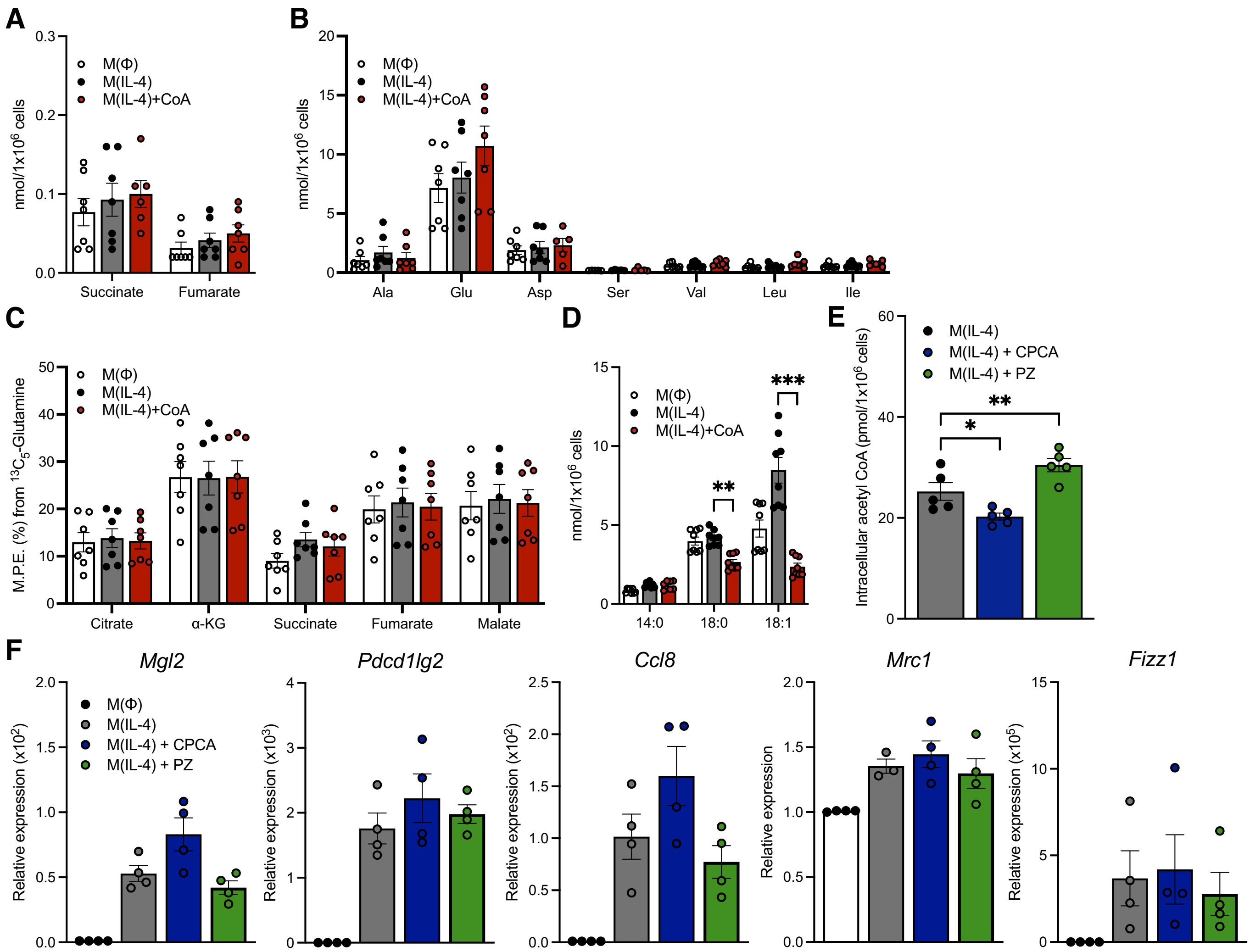

Figure EV2. CoA does not enhance alternative activation by increasing metabolic changes associated with the IL-4 response.

(**A**, **B**) Abundances of (**A**) TCA cycle intermediates and (**B**) amino acids in BMDMs treated with vehicle (Veh.), IL-4 (20 ng/mL), or IL-4 + 1 mM CoA for 48 h. ($n = 7$ independent biological replicates). (**C**) Enrichment from uniformly labeled $^{13}C_5$-glutamine into the TCA cycle intermediates citrate, α-ketoglutarate (α-KG), succinate, fumarate, and malate in BMDMs treated as in (**A**) ($n = 7$ independent biological replicates). (**D**) Quantification of newly synthesized myristic acid (14:0), stearic acid (18:0), and oleic acid (18:1) from BMDMs treated as in (**A**) (data shown as $n = 8$ technical replicates from $n = 2$ independent biological replicates) (**$P = 0.010$, ***$P < 0.001$). (**E**) Intracellular acetyl-CoA levels of BMDMs stimulated with vehicle [M(ϕ)], IL-4 (20 ng/mL), IL-4 + CPCA (1 mM), or IL-4 + PZ-2891 (10 μM) for 48 h ($n = 5$ independent biological replicates) (*$P = 0.02$, **$P = 0.006$). (**F**) qPCR analysis of the IL-4-associated genes *Mgl2, Pdcd1lg2, Ccl8, Mrc1, and Fizz1* in BMDMs treated as in (**E**) ($n = 4$ independent biological replicates). All data were presented as mean ± SEM with statistical analysis conducted on biological replicates, each of which included multiple technical replicates, unless otherwise indicated. Statistical analysis for (**D**) was performed as a two-way ANOVA followed by Tukey's post hoc multiple comparisons test. Statistical analysis for (**E**) was performed as a one-way ANOVA followed by Dunnett's post hoc multiple comparisons test. Source data are available online for this figure.

 © The Author(s)

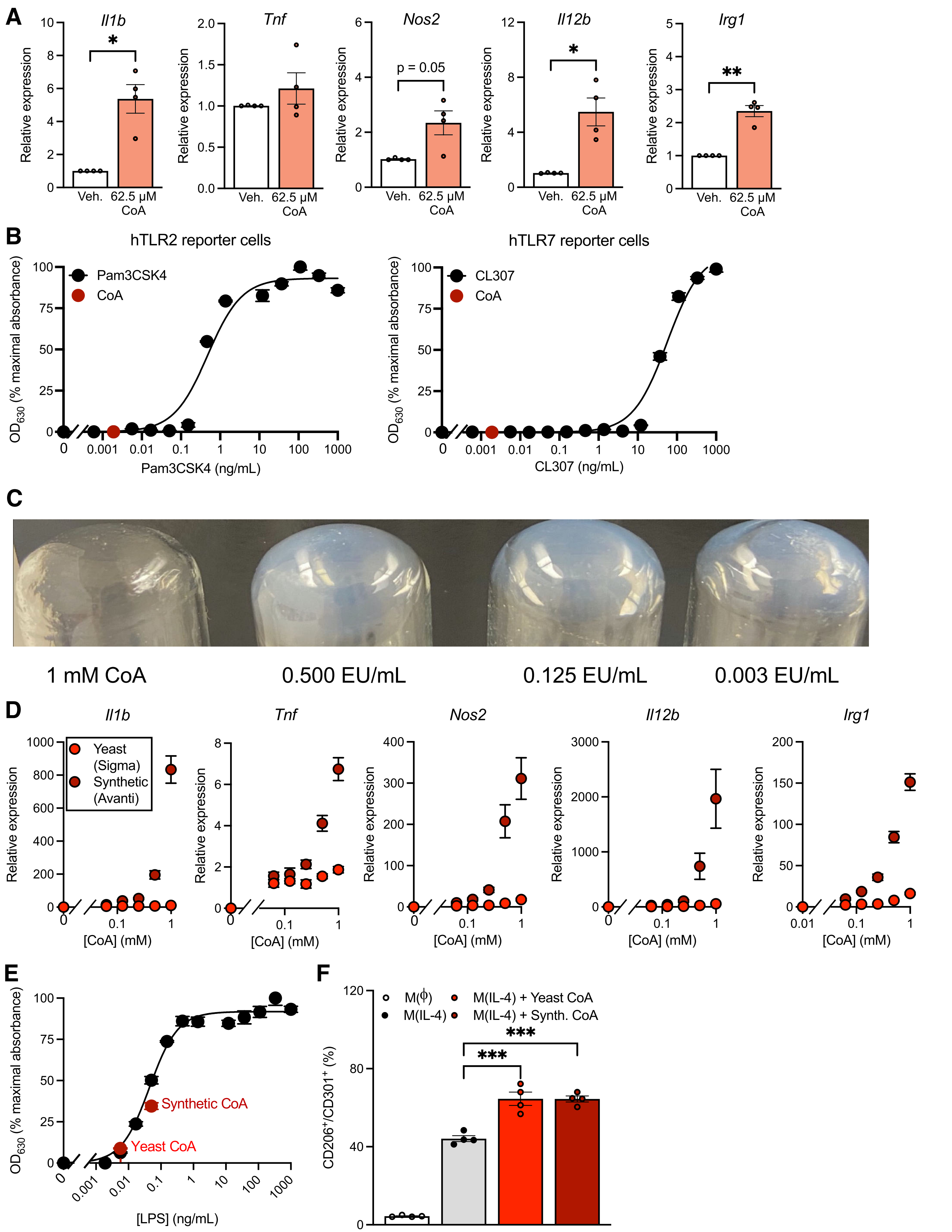
A
Il1b
Tnf
Nos2
Il12b
Irg1
Relative expression
Veh.
62.5 μM CoA
p = 0.05
B
hTLR2 reporter cells
hTLR7 reporter cells
Pam3CSK4
CoA
CL307
OD630 (% maximal absorbance)
Pam3CSK4 (ng/mL)
CL307 (ng/mL)
C
1 mM CoA
0.500 EU/mL
0.125 EU/mL
0.003 EU/mL
D
Yeast (Sigma)
Synthetic (Avanti)
[CoA] (mM)
E
Synthetic CoA
Yeast CoA
[LPS] (ng/mL)
F
M(ϕ)
M(IL-4)
M(IL-4) + Yeast CoA
M(IL-4) + Synth. CoA
CD206+/CD301+ (%)

Figure EV3. Coenzyme A increases pro-inflammatory gene expression.

(**A**) qPCR analysis of the pro-inflammatory genes *Il1b, Tnf, Nos2, Il12b*, and *Irg1* in BMDMs treated with vehicle (Veh.) or 62.5 μM CoA for 4 h ($n = 4$ independent biological replicates) (*$P = 0.02$ *Il1b*, *$P = 0.02$ *Il12b*, **$P = 0.004$). (**B**) Concentration-response curves of hTLR2 and hTLR7 reporter cells exposed to increasing concentrations of Pam3CSK4 and CL307, respectively (black dots). The red dot indicates the response elicited from 1 mM CoA ($n = 4$ independent biological replicates). (**C**) Limulus test, with opaque precipitate signifying the presence of endotoxin, for 1 mM yeast-derived CoA alongside endotoxin standards. The translucent tube shows that CoA is free of endotoxin (EU, endotoxin units). (**D**) Gene expression analysis of the pro-inflammatory genes *Il1b, Tnf, Nos2, Il12b*, and *Irg1* in BMDMs treated with yeast-derived CoA (Sigma-Aldrich) or synthetic CoA (Avanti Polar Lipids) with concentrations as indicated in the figure for 4 h ($n = 4$ independent biological replicates). (**E**) Concentration-response curves of hTLR4 reporter cells exposed to increasing concentrations of LPS (black dots). The red dots indicate the responses elicited from 1 mM synthetic CoA and 1 mM yeast-derived CoA ($n = 3$ independent biological replicates). (**F**) Percentage of $CD206^{+}/CD301^{+}$ population in BMDMs treated with vehicle [M(ϕ)], IL-4 (20 ng/mL), IL-4 + 1 mM yeast-derived CoA, or IL-4 + 1 mM synthetic CoA for 48 h ($n = 4$ independent biological replicates) (***$P < 0.001$). All data were presented as mean ± SEM with statistical analysis conducted on biological replicates, each of which included multiple technical replicates, unless otherwise indicated. Statistical analysis for (**A**) was performed as an unpaired, two-tailed *t*-test. Statistical analysis for (**F**) was performed as an ordinary one-way ANOVA followed by Dunnett's post hoc multiple comparisons test. Source data are available online for this figure.

© The Author(s)

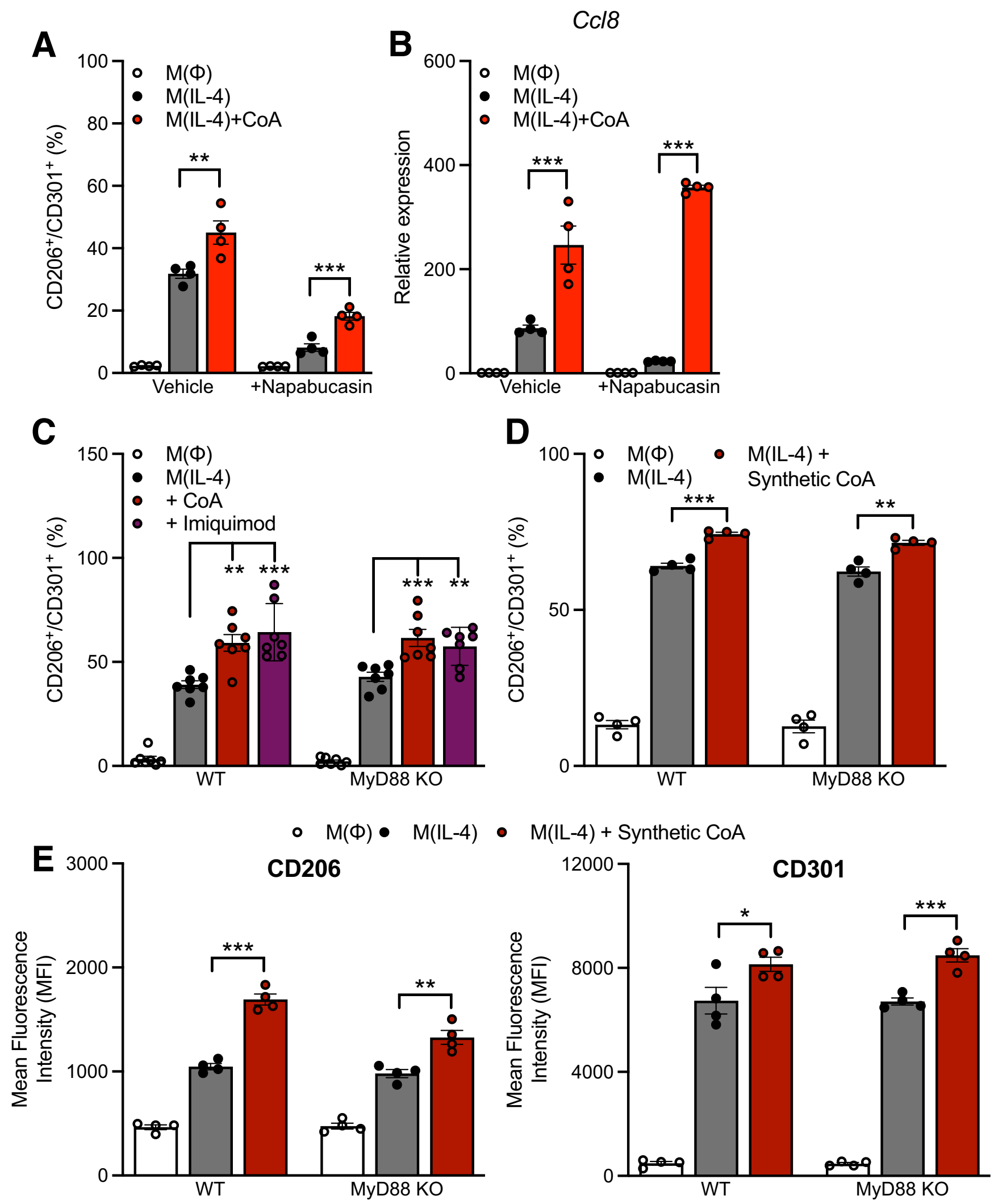

Figure EV4. Molecular mechanisms underlying augmentation of M(IL-4) by CoA.

(**A**) Percentage of CD206+/CD301+ population of BMDMs treated with vehicle [M(ϕ)], IL-4 (20 ng/mL), or IL-4 + 1 mM CoA with and without the STAT3 inhibitor Napabucasin (1μM) for 48 h ($n = 4$ independent biological replicates) (**$P = 0.005$, ***$P < 0.001$). (**B**) qPCR analysis of *Ccl8* in BMDMs treated as in (**A**) ($n = 4$ independent biological replicates) (***$P < 0.001$). (**C**) Percentage of CD206+/CD301+ population of BMDMs harvested from WT and *Myd88*$^{-/-}$ animals upon treatment with vehicle [M(ϕ)], IL-4 (20 ng/mL), IL-4 + yeast-derived CoA (1 mM), or IL-4 + imiquimod (10μM) for 48 h ($n = 7$ independent biological replicates) (**$P = 0.001$ WT, ***$P < 0.001$, **$P = 0.004$ KO). (**D, E**) Flow cytometric analysis of BMDMs harvested from WT and *Myd88*$^{-/-}$ animals upon treatment with vehicle [M(ϕ)], IL-4 (20 ng/mL), or IL-4 + synthetic CoA (1 mM) for 48 h. (**D**) Percentage of CD206+/CD301+ population (***$P < 0.001$, **$P = 0.004$) and (**E**) aggregate mean fluorescence intensity of CD206 and CD301 are presented ($n = 4$ independent biological replicates) (CD206: ***$P < 0.001$, **$P = 0.001$; CD301: *$P = 0.03$, ***$P < 0.001$). All data were presented as mean ± SEM with statistical analysis conducted on biological replicates, each of which included multiple technical replicates, unless otherwise indicated. Statistical analysis for (**A–E**) was performed as an ordinary one-way ANOVA followed by Dunnett's post hoc multiple comparisons test. Source data are available online for this figure.

© The Author(s)

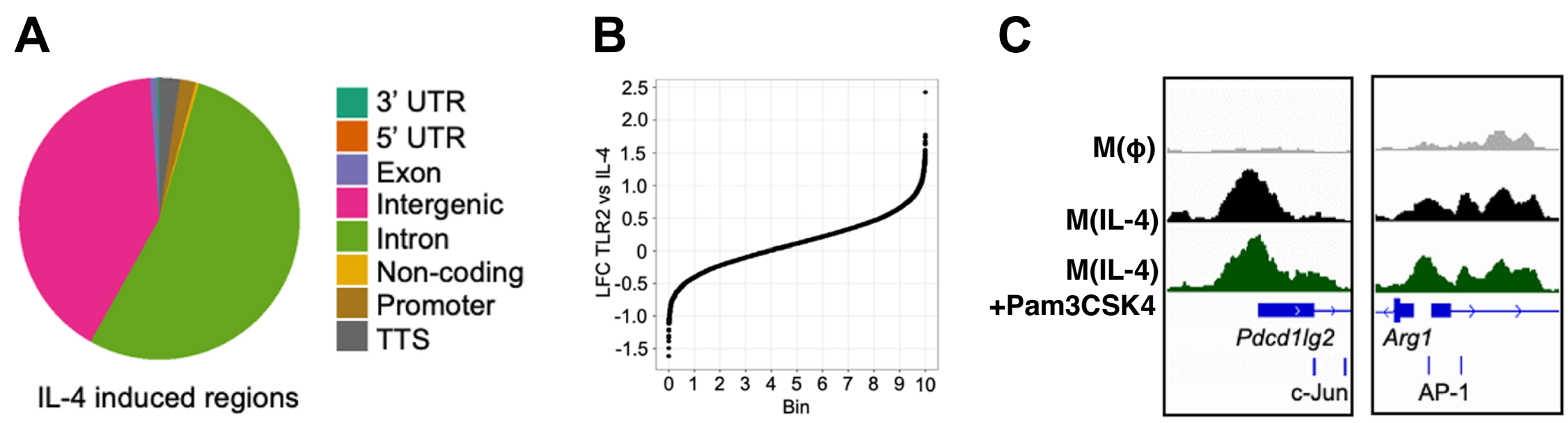

Figure EV5. Pam3CSK4 increases the chromatin accessibility of IL-4-induced regions.

(**A**) Genomic distribution of the 10,878 IL-4-induced accessibility regions. (**B**) Distribution of Pam3CSK4 (TLR2) and IL-4 co-treatment on the IL-4 inducible regions, divided into ten equal bins. (**C**) Representative tracks of the alternative activation genes *Pdcd1lg2* and *Arg1* promoter regions with nearby c-Jun/AP-1 motifs from BMDMs treated with vehicle [M(ϕ)], IL-4 (20 ng/mL), or IL-4 + 5 ng/mL Pam3CSK4 for 48 h.

 © The Author(s)
