# Supplementary material for: Coenzyme A activates TLR4 and enhances alternative macrophage activation in line with MyD88 agonism
Source: EMBO Rep. 2026 Sep 18;27(20):5927–52. doi: 10.1038/s44319-026-00925-y (PMC13694274; doi:10.1038/s44319-026-00925-y)
Supplement: Supplementary file 2 [file 44319_2026_925_MOESM1_ESM.pdf]

Coenzyme A activates TLR4 and enhances alternative macrophage activation in line with MyD88 agonism

Anthony Jones, Amy Rios, Neira Ibrahimovic, Andréa Ball, Carolina Chavez, Nicholas Bayley, Natalie Falta, Marisa Mekkittikul, Clarissa Carrillo, Wei Hsieh, Alessandro Sammarco, Amber Reyes, Angel Cortez, Thomas Graeber, Richard Watson, Alexander Hoffmann, Steven J. Bensinger, and Ajit Divakaruni

Corresponding author(s): Ajit Divakaruni (ADivakaruni@mednet.ucla.edu)

Review Timeline:

Transfer Date:	6th Oct 25
Editorial Decision:	4th Dec 25
Revision Received:	1st Jun 26
Editorial Decision:	14th Jul 26
Revision Received:	18th Aug 26
Accepted:	25th Aug 26

Editor: Kurt Weir / Esther Schnapp

Transaction Report: This manuscript was transferred to EMBO Reports following peer review at Review Commons.

(Note: With the exception of the correction of typographical or spelling errors that could be a source of ambiguity, letters and reports are not edited. Depending on transfer agreements, referee reports obtained elsewhere may or may not be included in this compilation. Referee reports are anonymous unless the Referee chooses to sign their reports.)

Review #1

1. Evidence, reproducibility and clarity:

Evidence, reproducibility and clarity (Required)

Jones et al. have submitted a manuscript detailing the role of Coenzyme A in the regulation of macrophage polarization. Overall, the manuscript is well designed, and the conclusions are well supported by the data. I find no major or minor deficiencies that need to be corrected.

2. Significance:

Significance (Required)

For decades the immunology community has boldly stated that mitochondrial metabolism not only provides the bioenergetics for cell expansion but also dictates cell fate. This has been especially true for fatty acid beta oxidation. Macrophage, T-cell and B-cell polarization have all been shown to require FAO for their polarization, but all based on one inhibitor. NONE of these observations hold up with more rigorous experimentation. The Divakaruni group has previously suggested that intracellular CoA homeostasis was the driver of macrophage differentiation as they could reverse the inhibitory effects by providing heroic levels of CoA extracellularly. Here, they have clarified the role of CoA. Intracellular CoA does not affect macrophage polarization/differentiation. This was done with cleaver manipulation of the CoA pools. Rather, extracellular CoA can act as a weak TLR4 ligand. This work nicely clarifies their previous work and further demonstrates a role for this metabolite as an endogenous activator of type 1 macrophages.

3. How much time do you estimate the authors will need to complete the suggested revisions:

Estimated time to Complete Revisions (Required)

(Decision Recommendation)

Less than 1 month

4. *Review Commons* values the work of reviewers and encourages them to get credit for their work. Select 'Yes' below to register your reviewing activity at Web of Science Reviewer Recognition Service (formerly Publons); note that the content of your review will not be visible on Web of Science.

Web of Science Reviewer Recognition

No

Review #2

1. Evidence, reproducibility and clarity:

Evidence, reproducibility and clarity (Required)

This is a fairly straightforward manuscript that indicated CoA acts as a "weak" TLR4 agonist and primes macrophages for alternative activation. Overall, the experiments are well done and clear enough. There are two major issues that need to be addressed:

1. Previous work has shown the following pathway: LPS>IL10>STAT3>IL4Ra>>>increased responsiveness to IL4/IL13 and increased expression of M2 associated markers (please note, this pathway does not apply to Arg1, often erroneously associated with M2 macrophages - LPS induces Arg1 far more than IL4 and this is independent of the STAT6 pathway - Dichtl et al., Science Advances and El Kasmi et al. Nature Immunology, and others). This pathway was first described in Lang et al. 2002 J. Immunol. Subsequently, other groups showed IL6 (Jens Brüning) and OSM (Carl Richards) do the same thing, which is not surprising given that they are STAT3 activators. Thus, Il4ra is a STAT3 target gene; this also makes sense in the kinetic evolution of macrophages from inflammatory to tissue reparative (if they survive). In my view, the authors have most likely found the same pathway. In Jones, expression of the IL4Ra was not quantified. Thus, the pathway described above needs to be accounted for. It may not apply here but seems the easiest explanation of the data.
2. Can the authors come up with a meaningful in vivo experiment to corroborate their data. Pantothenate-deficient mice have many phenotypes (not fully explored at all - PMID 31918006, for example) and pantothenate metabolism can be manipulated in different ways. Obviously, a complex in vivo experiment is not feasible here. But this should be discussed. What happens in human macrophages, where "polarization" is a completely different beast?

2. Significance:

Significance (Required)

This is a fairly straightforward manuscript that indicated CoA acts as a "weak" TLR4 agonist and primes macrophages for alternative activation. Overall, the experiments are well done and clear enough.

3. How much time do you estimate the authors will need to complete the suggested revisions:

Estimated time to Complete Revisions (Required)

(Decision Recommendation)

Between 1 and 3 months

4. *Review Commons* values the work of reviewers and encourages them to get credit for their work. Select 'Yes' below to register your reviewing activity at [Web of Science Reviewer Recognition Service] (formerly Publons); note that the content of your review will not be visible on Web of Science.

Web of Science Reviewer Recognition

No

Review #3

1. Evidence, reproducibility and clarity:

Evidence, reproducibility and clarity (Required)

Summary:

In this manuscript on enhancement of mIL-4 polarization by exogenous CoA, the authors follow up on their previous studies that had shown a correlation between Etomoxir-driven block in mIL-4 and a reduction of intracellular CoA levels. The results obtained (lack of enhancement of IL-4-induced changes in oxidative phosphorylation and glycolysis; lack of impact of pharmacological decrease/increase of intracellular CoA levels) led them to discard their initial hypothesis. Instead, the presence of a proinflammatory gene signature in macrophages treated with IL-4+CoA triggered experiments testing the involvement of TLR-Myd88 signaling and the identification of CoA as a weak agonist for TLR4 (which is consistent with a preprint manuscript posted in 2022 by others and showing induction of proinflammatory gene express in a TLR2/4-dependent manner).

Significance:

Overall, these results are novel and interesting, although the use of yeast-derived CoA

preparations raises a question about the contribution of contaminants that is only partially controlled by data obtained with a synthetic CoA. Regarding a biological role for CoA in macrophage biology in vivo, the authors propose that CoA may act as a DAMP upon release from dying/dead cells and thereby modify transcriptional polarization of m(IL-4). I have several comments related to specific experimental conditions and interpretation that should be addressed. Most importantly, the key findings of the manuscript should be demonstrated using synthetic CoA as described in comment #5.

Major comments:

1. Increasing/decreasing intracellular CoA levels does not alter IL-4-induced CD206 expression (Fig. 2i/j. However, the impact of CoA addition to mIL-4 is stronger for Ccl8 and Mgl2 mRNA (Fig. 1a) than for the CD206+ cell fraction (Fig. 1d). Therefore, it would be better (higher sensitivity) to include expression of these genes as readout after CPCA/PZ-2891 treatment.
2. The CoA-induced proinflammatory gene expression in Fig. 3c is relatively weak (e.g. compared to LPS). The authors use CoA throughout the manuscript at a concentration of 1 mM, and we do not know how much of it is required to cause an effect at all. Therefore, dose-response curves for the stimulation of macrophages with titrated amounts of CoA should be provided. In addition,
3. Related question: we are informed that the concentration of CoA in the mitochondrial matrix is 5mM, whereas cytosol contains 100µM. For CoA to act as DAMP, I would like to know the concentration of it in supernatants of cell cultures (live vs. dying/dead cells) and from tissues.
4. It is very good that the authors validate the findings obtained using the yeast-derived CoA with the synthetic molecule. It is very conceivable that the 15% contaminating substances in the yeast CoA could be causing the observed changes in m(IL-4). The fact that synthetic CoA has higher activity in proinflammatory gene expression by BMM (Suppl. Fig. S3) is reassuring, however, it raises the question why this is the case. One possibility is that the concentrations of the different CoA preps cannot directly be compared. Therefore, dose response curves should also be provided for synthetic CoA.
5. The difference in activity of yeast and synth. CoA could also be caused by the additional biologically active molecules in the yeast CoA. Therefore, it is important to show that the key findings in the paper (enhancement of m(IL-4) associated gene expression and CD206+ upregulation in vitro and in vivo) are also induced by synth. CoA. This is even more important in the context of the Myd88-independence of CD206+ upregulation in BMM treated with CoA (Suppl. Fig. S4). The experiment should be repeated with synth. CoA. If the enhancement of CD206+ cells induced by CoA is indeed unchanged in Myd88 KO BMM,

then the title of the manuscript "CoA enhances alternative macrophage activation via Myd88" would not be supported by the data and needed to be changed. Activation of the TLR4 reporter cell line should also be tested using the synth. CoA molecule.
6. The results from the tumor model in Fig. 5 are presented to show a stronger tumor-promoting effect of m(IL-4) stimulated with Pam3. However, the variability of the data is high and 2 out of 6 mice in the +Pam3 group appear to actually have a lower tumor weight than the control mice. Therefore, these data are quite superficial and preliminary, and would benefit from a replicate experiment. Furthermore, for the evaluation of CoA as a biologically relevant DAMP, it would be important to know whether CoA-treated m(IL-4) show the same tumor-promoting effect in vivo as Pam3.

Minor comments:

1. Fig. 1b: where the gates for CD206/CD301 set based on isotype control stainings?
2. The formatting not cohesive m(IL-4) vs. M(IL-4)
3. Methods: primer sequences are not shown. They should be provided.
4. Description of flowcytometry (L/D staining after surface? No washing steps after addition of L/D staining)
5. Statistics: the methods section states that variability is indicated by SD, but the Figure legends always mention SEM. Please correct.
6. A multitude of typos and editorial inconsistencies (e.g. spelling of m(IL-4), punctation and capitalization) should be corrected/streamlined.

2. Significance:

Significance (Required)

Strengths: I like that the authors follow up their previous work on Etomoxir and CoA, now finding again an unexpected twist in how the effect on m(IL-4) is brought about. This makes the story more complicated, but is important to get to a more precise and realistic understanding of metabolic and transcriptomic regulation and how they are interconnected (or not). In addition, the use of a relatively broad set of methods including ATACseq and mass spectrometry is a strength.

Weakness: the use of the not very pure yeast derived CoA prep, which is controlled for induction of proinflammatory cytokines by one experiment with synth. CoA. This validation needs to be expanded (see comments above) to substantiate the main message of the manuscript.

The scope of the manuscript is quite focussed on the mechanism of CoA enhanced m(IL-4). The finding that CoA appears not to act by changing intracellular macrophage metabolism but instead after its release by activation TLR4 widens the scope and suggests a new function for CoA as DAMP. This aspect would need to be further substantiated to be convincing.

Audience: scientists working at the intersection between metabolism and innate immunity will be interested in the results.

3. How much time do you estimate the authors will need to complete the suggested revisions:

Estimated time to Complete Revisions (Required)

(Decision Recommendation)

Between 3 and 6 months

4. *Review Commons* values the work of reviewers and encourages them to get credit for their work. Select 'Yes' below to register your reviewing activity at Web of Science Reviewer Recognition Service (formerly Publons); note that the content of your review will not be visible on Web of Science.

Web of Science Reviewer Recognition

Yes

Full Revision

Manuscript number: RC-2025-02881
Corresponding author(s): Ajit Divakaruni

[Please use this template only if the submitted manuscript should be considered by the affiliate journal as a full revision in response to the points raised by the reviewers.

If you wish to submit a preliminary revision with a revision plan, please use our "Revision Plan" template. ***It is important to use the appropriate template to clearly inform the editors of your intentions.****]*

1. General Statements [optional]

Prior to the point-by-point response to the reviewer, we would like to sincerely thank all the peer reviewers for their overwhelmingly positive comments and helpful suggestions. The recommendations have undoubtedly improved our initial submission, and we have done our best to incorporate as many of the suggestions as possible.

2. Point-by-point description of the revisions

This section is mandatory. Please insert a point-by-point reply describing the revisions that were already carried out and included in the transferred manuscript.

Our remarks in **black**; reviewer comments in *red italics*

Reviewer #1 *(Evidence, reproducibility and clarity (Required)):*
Jones et al. have submitted a manuscript detailing the role of Coenzyme A in the regulation of macrophage polarization. Overall, the manuscript is well designed, and the conclusions are well supported by the data. I find no major or minor deficiencies that need to be corrected.

Reviewer #1 (Significance (Required)):
For decades the immunology community has boldly stated that mitochondrial metabolism not only provides the bioenergetics for cell expansion but also dictates cell fate. This has been especially true for fatty acid beta oxidation. Macrophage, T-cell and B-cell polarization have all been shown to require FAO for their polarization, but all based on one inhibitor. NONE of these observations hold up with more rigorous experimentation. The Divakaruni group has previously suggested that intracellular CoA homeostasis was the driver of macrophage differentiation as they could reverse the inhibitory effects by providing heroic levels of CoA extracellularly. Here, they have clarified the role of CoA. Intracellular CoA does not affect macrophage polarization/differentiation. This was done with cleaver manipulation of the CoA pools. Rather, extracellular CoA can act as a weak TLR4 ligand. This work nicely clarifies their previous work and further demonstrates a role for this metabolite as an endogenous activator of type 1 macrophages.

We are thrilled by the positive comments about our work, and we are grateful the reviewer found our submission to be clarifying for the field and significant in the larger context of immunometabolism research.

Reviewer #2 *(Evidence, reproducibility and clarity (Required)):*

This is a fairly straightforward manuscript that indicated CoA acts as a "weak" TLR4 agonist and primes macrophages for alternative activation. Overall, the experiments are well done and clear enough. There are two major issues that need to be addressed:

We thank the reviewer for their positive comments regarding the quality and clarity of our work.

1. Previous work has shown the following pathway: LPS>IL10>STAT3>IL4Ra>>>increased responsiveness to IL4/IL13 and increased expression of M2 associated markers (please note, this pathway does not apply to Arg1, often erroneously associated with M2 macrophages - LPS induces Arg1 far more than IL4 and this is independent of the STAT6 pathway - Dichtl et al., Science Advances and El Kasmi et al. Nature Immunology, and others). This pathway was first described in Lang et al. 2002 J. Immunol. Subsequently, other groups showed IL6 (Jens Brüning) and OSM (Carl Richards) do the same thing, which is not surprising given that they are STAT3 activators. Thus, Il4ra is a STAT3 target gene; this also makes sense in the kinetic evolution of macrophages from inflammatory to tissue reparative (if they survive). In my view, the authors have most likely found the same pathway. In Jones, expression of the IL4Ra was not quantified. Thus, the pathway described above needs to be accounted for. It may not apply here but seems the easiest explanation of the data.

This is an excellent and important experiment suggested by the reviewer, and we address this in our revised **Supplemental Figure 5** and present the data in **Reviewer Figure R1a&b** here. To determine whether the effect of CoA can be explained simply by a STAT3-mediated effect on the IL-4 receptor, we treated cells with the well-characterized STAT3 inhibitor Napabucasin and measured whether CoA could enhance the macrophage IL-4 response. Two results are clear from the data:

(i) Treatment with Napabucasin reduced the expression of IL-4-linked cell surface markers and the IL-4 target gene *Ccl8*. This serves as an important control consistent with the *Il4ra* gene being a STAT3 target that increases IL-4 responsiveness.

(ii) Despite STAT3 inhibition and a reduced IL-4 response, CoA provision still augmented the IL-4-induced expression of *Ccl8* and the percentage of $CD206^{+}/CD301^{+}$ cells, indicating a STAT3-independent mechanism. The result aligns with our ATAC-Seq data in Figure 6 that shows broad changes in chromatin accessibility that cannot be completely explained by expression-level changes in the IL-4 receptor.

2. Can the authors come up with a meaningful in vivo experiment to corroborate their data. Pantothenate-deficient mice have many phenotypes (not fully explored at all - PMID 31918006, for example) and pantothenate metabolism can be manipulated in different ways. Obviously, a complex in vivo experiment is not feasible here. But this should be discussed. What happens in human macrophages, where "polarization" is a completely different beast?

We thank the reviewer for these thoughtful comments, and address the questions regarding *in vivo* proof-of-concept and polarization of human macrophages separately:

(i) Regarding the question of whether CoA can enhance the phenotype of IL-4-activated human macrophages, this is an excellent suggestion and we have added the data as **Figure 1h** and reproduced it below in **Reviewer Figure R1c**. Indeed, Coenzyme A dramatically amplifies expression of the human IL-4 responsive genes *CCL17, TGM2*, and *PDCD1LG2* (similarly to mouse macrophages). The result substantially expands the significance of our work by showing the phenotype is reproducible in both mouse and human macrophages – unlike many immunometabolic phenotypes – and we thank the reviewer again for suggesting this experiment.

(ii) With respect to an *in vivo* experiment to corroborate our data, we entirely agree with the reviewer regarding both the importance, but also the difficulty in interpretation, of an experiment genetically

manipulating CoA synthesis *in vivo*. As they have suggested, we raise these issues in the discussion on **Lines 370-377** of the revised manuscript. Here, we note the following points:

a. Wherever possible/appropriate (e.g. Figures 1g, 3f&g, 5g&h), we have sought to corroborate our *in vitro* findings with *in vivo*/*ex vivo* proofs-of-concept.
b. Studying immune phenotypes in pantothenate-deficient mice would be an exciting experiment in principle, but difficult to interpret if conducted. As noted by the reviewer in the work from Drs. Rock and Jackowski, knockout of one of four isoforms of pantothenate kinase (PANK) shows mild phenotypes consistent with compensation across isoforms for CoA provision. Global double knockout of PANK1 and PANK2, however, is postnatally lethal. Regardless, a tissue-specific double knockout in myeloid cells is unlikely to show a phenotype given our results showing that manipulating intracellular CoA levels in BMDMs does not alter the IL-4 response (Figs. 2h-j).
c. Given the established role of CoA in postnatal development, it would be difficult to attribute any immunologic phenotypes in genetically modified mice to direct effects of CoA as a metabolic DAMP as opposed to indirect effects from a chronically altered immune system.

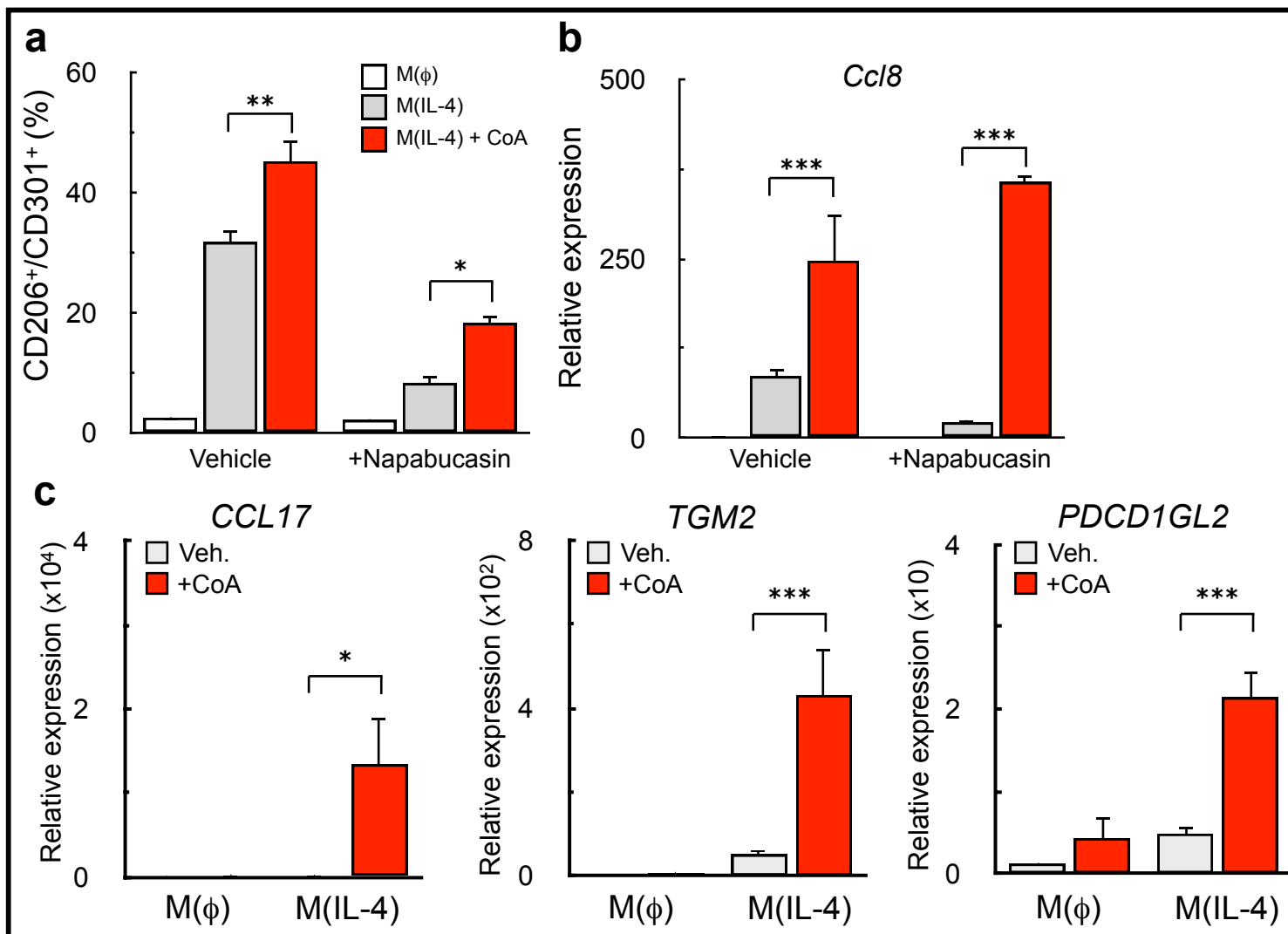

Reviewer Figure 1 – (a&b) Bone marrow-derived macrophages (BMDMs) were treated with combinations of either vehicle controls, IL-4 (20 ng/mL), CoA (1 mM), or the STAT3 inhibitor napabucasin (1 μM) for 48 hr. All compounds were added to the culture medium at the same time (i.e. all agents were given as co-treatments). This data is available in the revised manuscript as **Supplemental Figure 4**. (n=4 biological replicates from individual animals) **(c)** Single donor human peripheral blood mononuclear cells (PBMC) were obtained from the UCLA CFAR Virology Core lab, and monocyte isolation and macrophage differentiation were conducted as described in the manuscript methods. Vehicle controls or CoA (1 mM) were provided for 48 hr. similarly to mouse macrophages, showing a robust enhancement of the IL-4 response in human monocyte-derived macrophages. This data is available in the revised manuscript as **Figure 1h**. (n=3-5 biological replicates from individual human donors). Additional statistical information for all experiments is available in the main manuscript.

Reviewer #2 (Significance (Required)):

This is a fairly straightforward manuscript that indicated CoA acts as a "weak" TLR4 agonist and primes macrophages for alternative activation. Overall, the experiments are well done and clear enough.

We reiterate our gratitude for the comments on the quality and clarity of our work.

Reviewer #3 *(Evidence, reproducibility and clarity (Required)):*
Summary:
In this manuscript on enhancement of mIL-4 polarization by exogenous CoA, the authors follow up on their previous studies that had shown a correlation between Etomoxir-driven block in mIL-4 and a reduction of intracellular CoA levels. The results obtained (lack of enhancement of IL-4-induced changes in oxidative phosphorylation and glycolysis; lack of impact of pharmacological decrease/increase of intracellular CoA levels) led them to discard their initial hypothesis. Instead, the presence of a proinflammatory gene signature in macrophages treated with IL-4+CoA triggered experiments testing the involvement of TLR-Myd88 signaling and the identification of CoA as a weak

agonist for TLR4 (which is consistent with a preprint manuscript posted in 2022 by others and showing induction of proinflammatory gene express in a TLR2/4-dependent manner).

Significance:
Overall, these results are novel and interesting, although the use of yeast-derived CoA preparations raises a question about the contribution of contaminants that is only partially controlled by data obtained with a synthetic CoA. Regarding a biological role for CoA in macrophage biology in vivo, the authors propose that CoA may act as a DAMP upon release from dying/dead cells and thereby modify transcriptional polarization of m(IL-4). I have several comments related to specific experimental conditions and interpretation that should be addressed. Most importantly, the key findings of the manuscript should be demonstrated using synthetic CoA as described in comment #5.

We are heartened that the reviewer found our initial submission to be novel and interesting, and are grateful for their suggestions to reinforce our existing data with more studies comparing yeast-derived and synthetically-derived Coenzyme A. We have done our best to address each of the individual questions below:

Major comments:

1. Increasing/decreasing intracellular CoA levels does not alter IL-4-induced CD206 expression (Fig. 2i/j. However, the impact of CoA addition to mIL-4 is stronger for Ccl8 and Mgl2 mRNA (Fig. 1a) than for the CD206+ cell fraction (Fig. 1d). Therefore, it would be better (higher sensitivity) to include expression of these genes as readout after CPCA/PZ-2891 treatment.

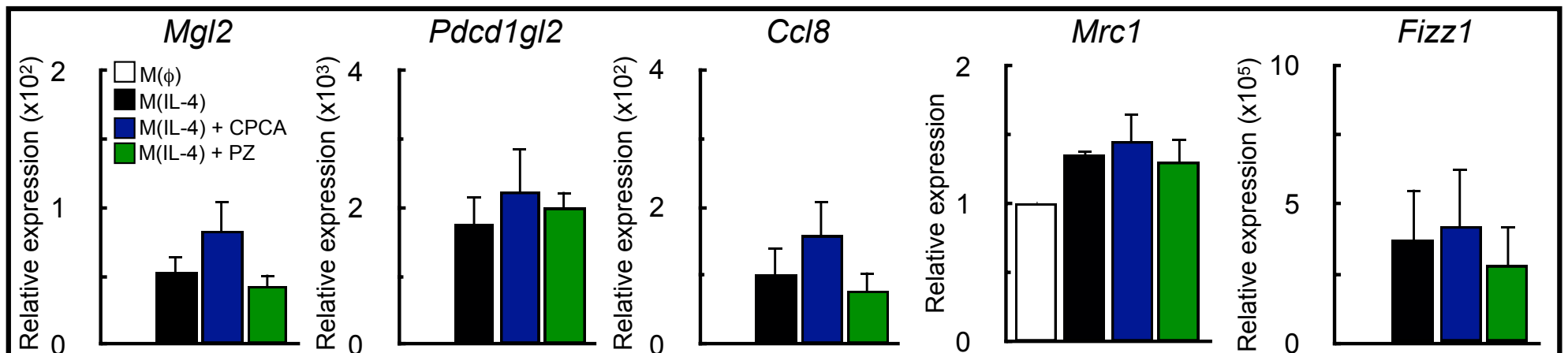

Reviewer Figure R2 – Bone marrow-derived macrophages were treated with combinations of either vehicle controls, IL-4 (20 ng/mL), IL-4 + CPCA (1 mM), or IL-4+PZ-2891 for 48 hr. All compounds were added to the culture medium at the same time (i.e. all agents were given as co-treatments), and methodological details are provided in the main text. This data is available in the revised manuscript as **Supplemental Figure S2f**. (n=4 biological replicates from individual animals)

This is a helpful suggestion, and we have now conducted gene expression studies to complement our flow cytometry and mass spectrometry studies while manipulating the intracellular CoA pool. In line with our previous work, neither CPCA (which decreases intracellular free CoA) or PZ-2891 (which increases intracellular free CoA) meaningfully alter expression of IL-4-linked genes including *Ccl8* or *Mgl2*. In fact, the only (statistically insignificant) trend *refutes* the hypothesis, as gene expression with CPCA leads to marginally increased gene expression. These results are now included in **Supplemental Figure S2f** and reproduced here in **Reviewer Figure R2**. We thank the reviewer for this helpful suggestion, as it has strengthened our conclusion that intracellular CoA levels do not adjust the macrophage IL-4 response.

2. The CoA-induced proinflammatory gene expression in Fig. 3c is relatively weak (e.g. compared to LPS). The authors use CoA throughout the manuscript at a concentration of 1 mM, and we do not know how much of it is required to cause an effect at all. Therefore, dose-response curves for the stimulation of macrophages with titrated amounts of CoA should be provided. In addition,

We thank the reviewer for bringing up this point so we could clarify and add to our existing data. We should note that Supplemental Figures 1b&c of our previous submission (and resubmitted manuscript) detail a concentration-response curve showing that at little as 62.5 μM CoA – the lowest concentration tested – was sufficient to enhance IL-4 cell surface marker expression.

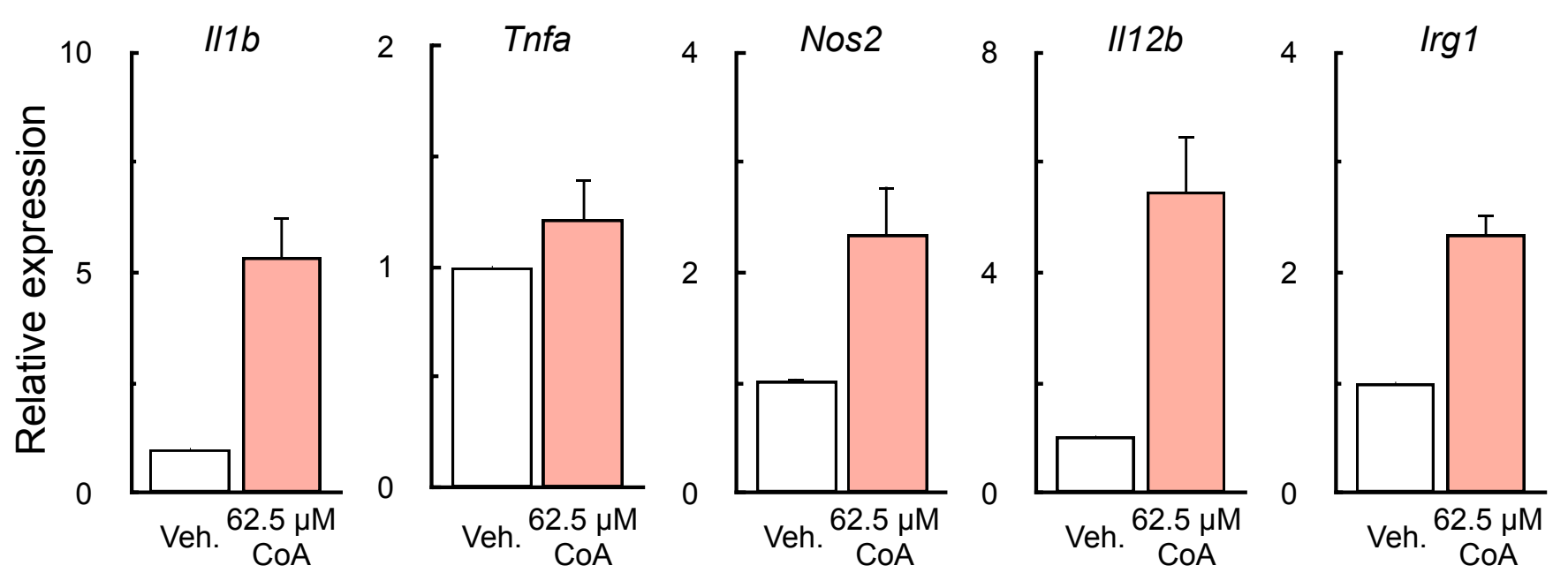

Reviewer Figure R3 – Bone marrow-derived macrophages were treated with either vehicle controls or 62.5 µM CoA for 4 hr. and pro-inflammatory gene expression was measured by qPCR. Methodological details are provided in the main text. This data is available in the revised manuscript as **Supplemental Figure S3.** (n=4 biological replicates from individual animals)

However, it is an excellent suggestion as the reviewer notes, to conduct a similar concentration-response to determine if this lines up with CoA inducing a pro-inflammatory response. The full data set is presented in the answer to reviewer question 4 (comparing CoA purchased from Sigma vs. Avanti Polar Lipids), but here we present **Reviewer Figure R3** showing that 62.5 µM CoA is sufficient to elicit a pro-inflammatory response. Though it is indeed a weak effect as noted by the reviewer, our data suggest that the relatively mild stimulus is crucial for the effect. Given the results with the TLR3 agonist Poly I:C (Figure 5), which engages a Type 1 interferon response, strong TLR4 agonists that engage the TRIF/Type I interferon arm of the TLR4 response are likely to blunt or block the IL-4 response. This data is now available in the revised manuscript as **Supplemental Figure S3**.

3. Related question: we are informed that the concentration of CoA in the mitochondrial matrix is 5mM, whereas cytosol contains 100µM. For CoA to act as DAMP, I would like to know the concentration of it in supernatants of cell cultures (live vs. dying/dead cells) and from tissues.

This is an important point brought up by the reviewer, and we agree that the implicit issue raised (i.e. "do the concentrations of CoA required to see an effect reconcile with a physiological role as a DAMP?") should be more thoroughly addressed in the manuscript. Tissue concentrations of free CoA (in ng/mg tissue) are well established for mice and range from >100 nmol/g tissue (liver, heart, brown adipose tissue) to <40 nmol/g tissue (skeletal muscle, testes, white adipose tissue) (PMID: 25109998). The concentration in cell supernatants from dead/dying cells would be dependent on the cell number plated and the volume in which they are grown, and would not be representative of the cellular density of non-hematopoietic tissues in almost all *in vitro* setups.

Nonetheless, the reviewer's larger point is very well reasoned, and we address it in the following ways in the discussion on **Lines 378-391.**

- In light of the reviewer's comment, we now mention specific instances in the discussion where CoA acting as a DAMP may reasonably play a physiological role (e.g. acetaminophen-induced acute liver injury or other forms of sterile liver injury given that DAMPs are known to be important factors and liver tissue contains relatively high concentrations of CoA).
- Although cytoplasmic concentrations of CoA may only be 50-100 µM, our work establishes a framework for how ubiquitous metabolic co-factors can activate pattern recognition receptors. Put another way, although CoA itself may not be a physiologically relevant DAMP, discovering this pathway could inform how other nucleotide or nucleoside analogs (e.g. adenine- or adenosine-containing molecules present at millimolar concentrations) exert their effects on innate immunity.
- Our newly obtained data with HMDMs (**Figure 1h** and **Reviewer Figure R1c**) shows that the CoA response in human macrophages – boosting IL-4-linked gene expression by 10-100X – may be much stronger than the 1.5-5X effect observed in mouse BMDMs. As such, it is exciting to speculate that CoA may have a more potent effect on the IL-4 response in humans relative to mice. We trust the reviewer understands the limitations of obtaining human macrophages that preclude conducting a thorough concentration-response analysis given the restrictions of a manuscript revision.

4. It is very good that the authors validate the findings obtained using the yeast-derived CoA with the synthetic molecule. It is very conceivable that the 15% contaminating substances in the yeast CoA

could be causing the observed changes in m(IL-4). The fact that synthetic CoA has higher activity in proinflammatory gene expression by BMM (Suppl. Fig. S3) is reassuring, however, it raises the question why this is the case. One possibility is that the concentrations of the different CoA preps cannot directly be compared. Therefore, dose response curves should also be provided for synthetic CoA.

This is an astute observation by the reviewer and we thank them for reading our manuscript with such detailed attention to pick this up. We are reassured that the reviewer shares our interpretation that the effect of CoA is not due to a contaminating TLR4 agonist in the yeast-derived preparation (from Sigma-Aldrich; ~85% pure) given a negative Limulus Test (**Supplemental Figure S4b**). Moreover, the synthetically-derived preparation (from Avanti Polar Lipids; ~99% pure) yields a stronger TLR4 response.

An exploration of the follow-on question regarding why the effect is greater than 15% is presented below. These experiments have been added to **Supplemental Figure S4c&d** and are reproduced below here in **Reviewer Figure R4**. The summary of our data suggests the individual concentrations indeed cannot be compared – matched concentrations of synthetic Avanti CoA have greater than a 15% effect than yeast-derived Sigma CoA. There are likely multiple factors that could explain this, some of which are listed below.

- The physiological effect of a TLR agonist need not be linear with its concentration, as demonstrated by the sigmoidal calibration curves for the TLR-expressing HEK-blue cells (**Figures 4b, S4a**). This likely does not explain the dramatic difference between the two CoA preparations but is worth noting.

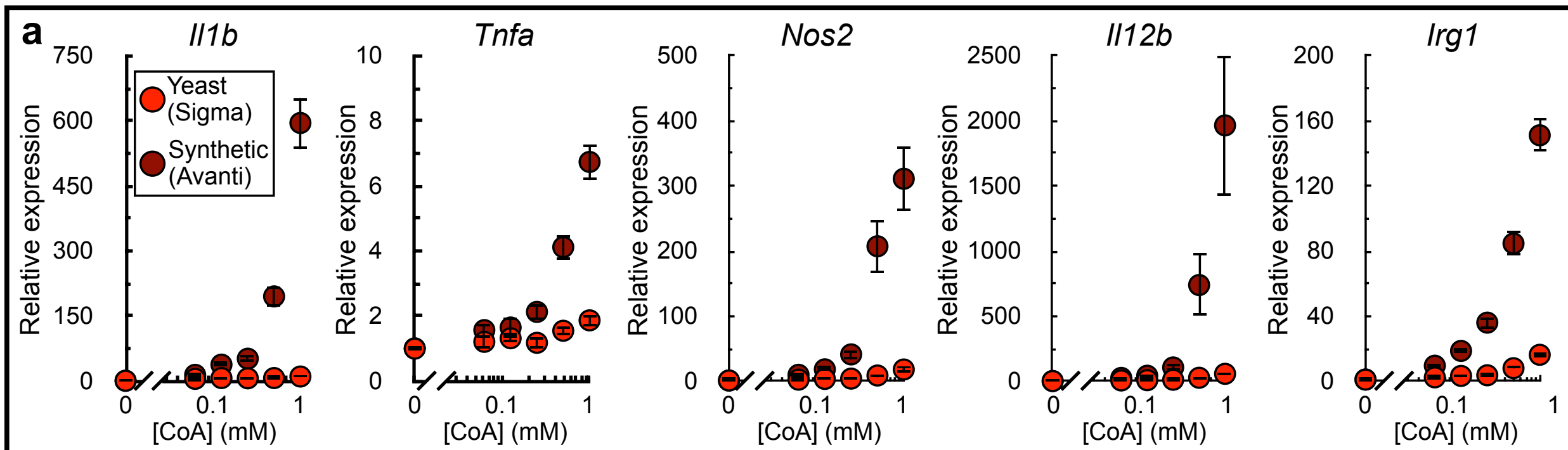

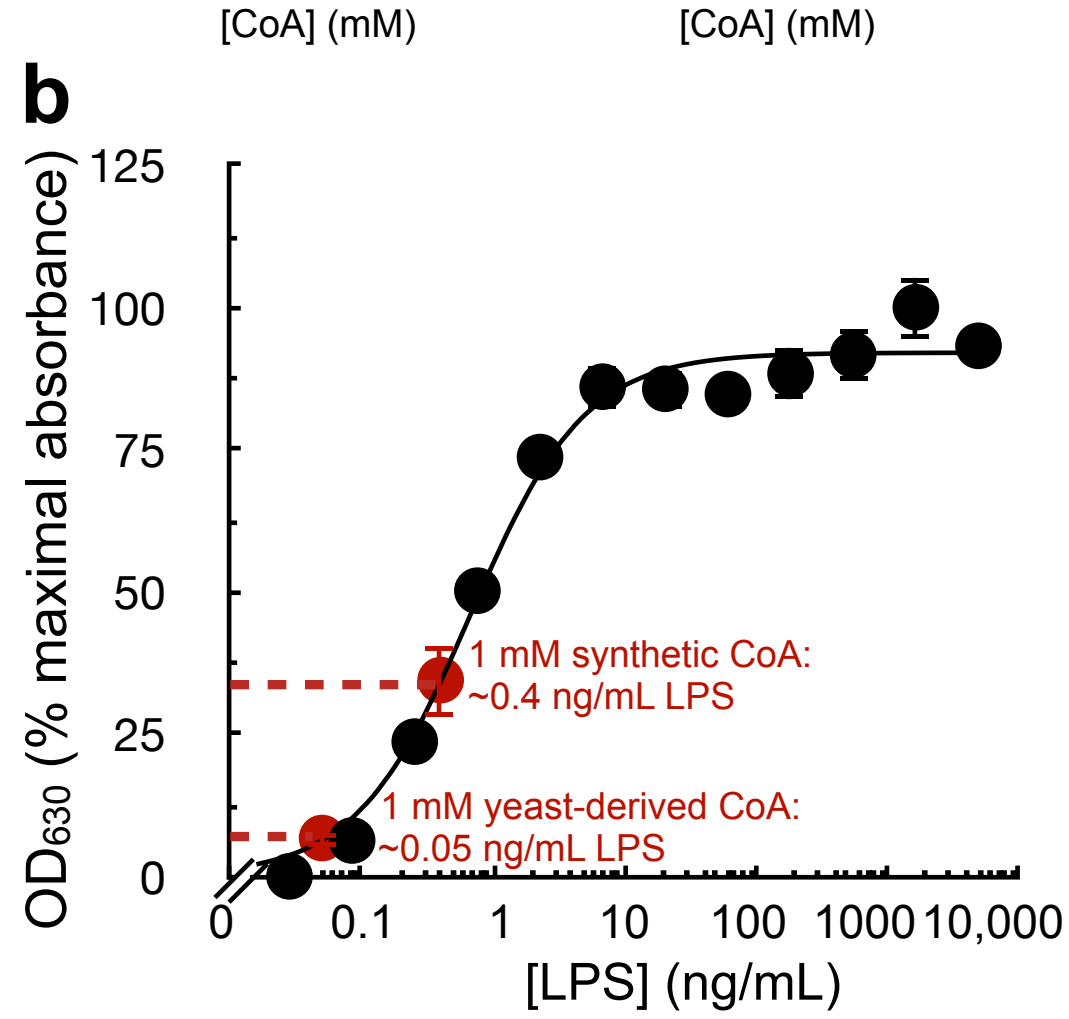

Reviewer Figure R4 – (a) Bone marrow-derived macrophages (BMDMs) were treated with a concentration-response of either yeast-derived CoA from Sigma-Aldrich or synthetically-derived CoA from Avanti Polar lipids. BMDMs were treated for 4 hr. and pro-inflammatory gene expression was measured by qPCR. Methodological details are provided in the main text. (n=4 biological replicates from individual animals) **(b)** The degree of TLR4 agonism from both yeast and synthetic CoA (1 mM) were calibrated against LPS in the HEK-Blue heterologous expression system as described in the main text. (n=3 independent replicates from individual cell passages). These data are available in the revised manuscript as **Supplemental Figures S4c&d.**

- While we have determined that the 15% contaminating substances in the yeast-derived CoA are not causing the observed changes in the IL-4 response (see **Reviewer Figure R5**), it is formally possible that there are contaminating substances blunting the pro-inflammatory response and therefore limiting the effect of CoA purchased from Sigma-Aldrich relative to that from Avanti Polar Lipids.

Importantly, however, our data in response to Reviewer Question #5 show there is no difference in amplifying the IL-4 response between the yeast- and synthetically-derived CoA.

5. The difference in activity of yeast and synth. CoA could also be caused by the additional biologically active molecules in the yeast CoA. Therefore, it is important to show that the key findings in the paper (enhancement of m(IL-4) associated gene expression and CD206+ upregulation in vitro and in vivo) are also induced by synth. CoA. This is even more important in the context of the Myd88-independence of CD206+ upregulation in BMM treated with CoA (Suppl. Fig. S4). The experiment should be repeated with synth. CoA. If the enhancement of CD206+ cells induced by CoA is indeed unchanged in Myd88 KO BMM, then the title of the manuscript "CoA enhances alternative macrophage activation via Myd88" would not be supported by the data and needed to be changed. Activation of the TLR4 reporter cell line should also be tested using the synth. CoA molecule.

We are grateful for this suggestion by the reviewer to further cement the idea that our observation of CoA enhancing the macrophage IL-4 response was not due to a contaminant in the Sigma-Aldrich CoA preparation. The reviewer makes a few points in this question which we address individually here.

- The suggestion to confirm that the CoA-induced enhancement of M(IL-4) is not due to a contaminating substance in the Sigma-Aldrich CoA is excellent and necessary. Here we show that synthetically derived CoA (99% pure, purchased from Avanti Polar Lipids) quantitatively reproduces the effect from yeast-derived CoA from Sigma-Aldrich in **Supplemental Figure S4e** and reproduced here in **Reviewer Figure R5a**. The response is noteworthy because synthetic CoA has profoundly stronger pro-inflammatory response than yeast-derived CoA, yet both have a similar effect on augmenting M(IL-4). This suggests that any appropriate pro-inflammatory response – irrespective of the relative strength or weakness – is sufficient to maximize the effect. This can also be observed with the range of MyD88-linked TLR agonists used in Figures 5 and S6a.
- Similarly, we also conducted experiments to show that the effect of synthetic CoA on M(IL-4) is independent of MyD88 similarly to yeast-derived CoA. These data are present in **Supplemental Figure S6b&c** and reproduced here in **Reviewer Figure R5b&c**. Here again, we should note that the effect of synthetic CoA is quantitatively similar to the effect of yeast CoA and Imiquimod (Supplemental Figure S6a).
- Activation of the TLR4 reporter cell line was presented above in **Reviewer Figure R4** and is available in **Supplemental Figure S4c**.
- Regarding the title of the manuscript, we acknowledge that we struggled a bit with how to frame our findings. Importantly, our findings support a model where (i) CoA provision enhances the IL-4 response not via metabolic changes but rather by acting as a mild pro-inflammatory stimulus, and (ii) MyD88

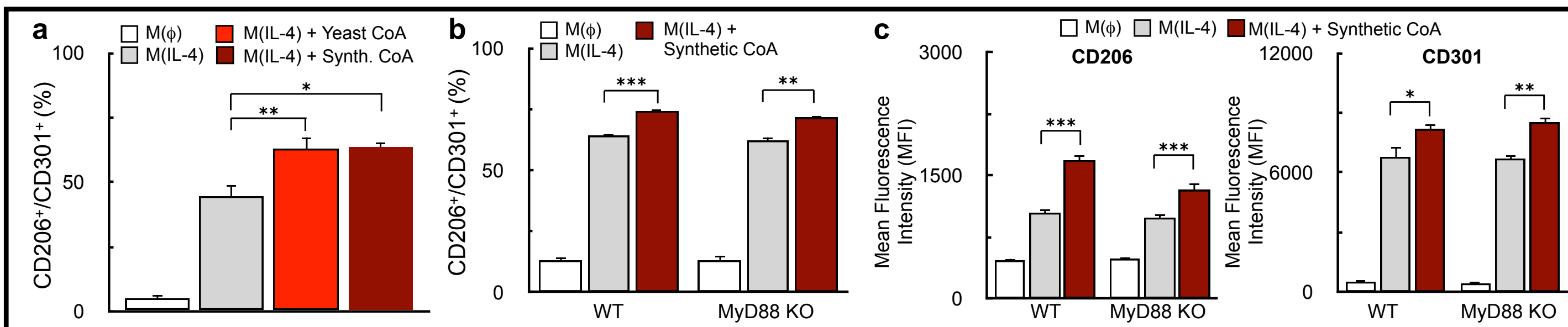

Reviewer Figure R5 – (a) Bone marrow-derived macrophages (BMDMs) were treated with vehicle control, IL-4 (20 ng/mL), IL-4 with yeast-derived CoA from Sigma-Aldrich (1 mM), or synthetically-derived CoA from Avanti Polar lipids (1mM) for 48 hr. Flow cytometry for CD206+/CD301+ cells was conducted as described in the main text. **(b&c)** BMDMs isolated from either wild-type (WT) or MyD88-null (MyD88 KO) mice were treated with vehicle controls, IL-4, or IL-4 with synthetically-derived CoA as in (a). Flow cytometry was conducted as described in the main text. These data are available in **Supplemental Figures S4e** and **S6b&c**. (n=4 biological replicates from individual animals for all experiments)

signaling augments the IL-4 response. We should also note that our findings simply show that CoA does not *exclusively* enhance the IL-4 response via MyD88 signaling, and there may be other redundant pathways (similarly to MyD88 agonist imiquimod but unlike the MyD88 agonists Pam3-CSK4 and low concentrations of LPS). We are open to working journal editors to strike the right balance of scientific accuracy and representation of the work when deciding on a final title.

6. The results from the tumor model in Fig. 5 are presented to show a stronger tumor-promoting effect of m(IL-4) stimulated with Pam3. However, the variability of the data is high and 2 out of 6 mice in the +Pam3 group appear to actually have a lower tumor weight than the control mice. Therefore, these data are quite superficial and preliminary, and would benefit from a replicate experiment. Furthermore, for the evaluation of CoA as a biologically relevant DAMP, it would be important to know whether CoA-treated m(IL-4) show the same tumor-promoting effect in vivo as Pam3.

We thank the reviewer for their comment, and agree that our *in vivo* work is indeed preliminary. Our goal with this report was to focus on the initial discovery of this molecular pathway and its first, broad characterization using a range of techniques (e.g. *in vivo* outcomes, ATAC-Seq, etc.), many of which can spur more detailed follow-up studies for future papers. As detailed in the manuscript discussion (**Lines 415-419**), future work beyond our initial discovery is warranted to thoroughly explore the physiological outcomes of CoA as a metabolic DAMP in relevant model systems such as acute liver injury. As an initial proof-of-concept to show that MyD88 signaling can enhance alternative activation, however, we believe our two discrete experiments (sterile inflammation and tumor formation) are sufficient to indicate the phenotype is likely relevant in animal models. *In vivo* syngeneic tumor models display natural variability in tumor size due to differences in implantation efficiency, host immune responses, and tumor-intrinsic growth kinetics. Nonetheless, our statistical analysis demonstrates that, with high confidence, that the observed differences are reproducible and not attributable to random variation.

Minor comments:

1. Fig. 1b: where the gates for CD206/CD301 set based on isotype control stainings?

We thank the reviewer for pointing out this oversight in our methods. The gates were indeed set on isotype control stains, and this is now mentioned in **Lines 519-521** of the revised manuscript.

2. The formatting not cohesive m(IL-4) vs. M(IL-4)

Again, this is an embarrassing oversight on our part and we have done our very best to copy edit the piece and remove any inconsistencies and errors.

3. Methods: primer sequences are not shown. They should be provided.

We thank the reviewer for pointing this out, and now include all primer sequences used in Supplemental Table 1 of the revised manuscript.

4. Description of flowcytometry (L/D staining after surface? No washing steps after addition of L/D staining)

We thank the reviewer for pointing out another oversight in our methods, and have provided a more detailed description of the flow cytometric analysis in **Lines 509-521** of the revised manuscript.

5. Statistics: the methods section states that variability is indicated by SD, but the Figure legends always mention SEM. Please correct.

We are grateful for the reviewer's helpful attention to detail, and have corrected the methods to line up with the figure legends.

6. A multitude of typos and editorial inconsistencies (e.g. spelling of m(IL-4), punctation and capitalization) should be corrected/streamlined.

We are grateful for the reviewer's helpful attention to detail, and have done our best to copy edit the manuscript prior to resubmission.

Reviewer #3 (Significance (Required)):

strengths: I like that the authors follow up their previous work on Etomoxir and CoA, now finding again an unexpected twist in how the effect on m(IL-4) is brought about. This makes the story more complicated, but is important to get to a more precise and realistic understanding of metabolic and transcriptomic regulation and how they are interconnected (or not). In addition, the use of a relatively broad set of methods including ATACseq and mass spectrometry is a strength.

weakness: the use of the not very pure yeast derived CoA prep, which is controlled for induction of proinflammatory cytokines by one experiment with synth. CoA. This validation needs to be expanded (see comments above) to substantiate the main message of the manuscript.

The scope of the manuscript is quite focussed on the mechanism of CoA enhanced m(IL-4). The finding that CoA appears not to act by changing intracellular macrophage metabolism but instead after its release by activation TLR4 widens the scope and suggests a new function for CoA as DAMP. This aspect would need to be further substantiated to be convincing.

Audience: scientists working at the intersection between metabolism and innate immunity will be interested in the results.

We thank the reviewer for their kind comments regarding the precision, credibility, and breadth of our manuscript. We hope they find our revised manuscript an improvement over our previous submission regarding both the new experiments and modified text. **The comments have undoubtedly improved our manuscript and we are grateful to the reviewer for the considerable effort they put into reading our submission.**

1st Editorial Decision **4th Dec 2025**

Dear Dr. Divakaruni

Thank you for the transfer of your revised manuscript to our journal.

As you will see, the referees acknowledge that the findings are interesting and that the conclusions are overall supported by the data with the main remaining comment relating to the title of the paper.

Given this positive feedback, we would like you to address the final referee comments before we can proceed with the official acceptance of your ms.

When submitting your revised manuscript, we will require:

1) a .docx formatted version of the manuscript text (including legends for main figures, EV figures and tables). Please make sure that the changes are highlighted to be clearly visible.

2) individual production quality figure files as .eps, .tif, .jpg (one file per figure).
Please download our Figure Preparation Guidelines (figure preparation pdf) from our Author Guidelines pages https://www.embopress.org/page/journal/14693178/authorguide for more info on how to prepare your figures.

3) a .docx formatted letter INCLUDING the reviewers' reports and your detailed point-by-point responses to their comments. As part of the EMBO Press transparent editorial process, the point-by-point response is part of the Review Process File (RPF), which will be published alongside your paper.

4) a complete author checklist, which you can download from our author guidelines (). Please insert information in the checklist that is also reflected in the manuscript. The completed author checklist will also be part of the RPF.

5) Please note that all corresponding authors are required to supply an ORCID ID for their name upon submission of a revised manuscript (). Please find instructions on how to link your ORCID ID to your account in our manuscript tracking system in our Author guidelines
()

6) We replaced Supplementary Information with Expanded View (EV) Figures and Tables that are collapsible/expandable online. A maximum of 5 EV Figures can be typeset. EV Figures should be cited as 'Figure EV1, Figure EV2" etc... in the text and their respective legends should be included in the main text after the legends of regular figures.

- For the figures that you do NOT wish to display as Expanded View figures, they should be bundled together with their legends in a single PDF file called *Appendix*, which should start with a short Table of Content. Appendix figures should be referred to in the main text as: "Appendix Figure S1, Appendix Figure S2" etc. See detailed instructions regarding expanded view here:

- Additional Tables/Datasets should be labeled and referred to as Table EV1, Dataset EV1, etc. Legends have to be provided in a separate tab in case of .xls files. Alternatively, the legend can be supplied as a separate text file (README) and zipped together with the Table/Dataset file.

7) Before submitting your revision, primary datasets (and computer code, where appropriate) produced in this study need to be deposited in an appropriate public database (see <
https://www.embopress.org/page/journal/14693178/authorguide#dataavailability>).
Please remember to provide a reviewer password if the datasets are not yet public.

The accession numbers and database should be listed in a formal "Data Availability " section (placed after Materials & Method) that follows the model below (see also < https://www.embopress.org/page/journal/14693178/authorguide#dataavailability>). Please note that the Data Availability Section is restricted to new primary data that are part of this study.

Data availability

The datasets (and computer code) produced in this study are available in the following databases:

- RNA-Seq data: Gene Expression Omnibus GSE46843 (https://www.ncbi.nlm.nih.gov/geo/query/acc.cgi?acc=GSE46843)
- [data type]: [name of the resource] [accession number/identifier/doi] ([URL or identifiers.org/DATABASE:ACCESSION])

*** Note - All links should resolve to a page where the data can be accessed. ***

8) At EMBO Press we ask authors to provide source data for the main manuscript figures. You will receive a separate email with instructions for providing source data with your revised manuscript, including how to upload and organize the files.

Additional information on source data and instruction on how to label the files are available

9) The journal requires a statement specifying whether or not authors have competing interests (defined as all potential or actual interests that could be perceived to influence the presentation or interpretation of an article). In case of competing interests, this must be specified in your disclosure statement. Further information: https://www.embopress.org/competing-interests

10) Figure legends and data quantification:
The following points must be specified in each figure legend:

- the name of the statistical test used to generate error bars and P values,
- the EXACT p-values,
- the number (n) of independent experiments (please specify technical or biological replicates) underlying each data point,
- the nature of the bars and error bars (s.d., s.e.m.)

- If the data are obtained from n {less than or equal to} 5, show the individual data points in addition to the SD or SEM.
- If the data are obtained from n {less than or equal to} 2, use scatter blots showing the individual data points.

Discussion of statistical methodology can be reported in the materials and methods section, but figure legends should contain a basic description of n, P and the test applied.

See also the guidelines for figure legend preparation:
https://www.embopress.org/page/journal/14693178/authorguide#figureformat

- Please also include scale bars in all microscopy images and define their size in the legend, not the image.

11) Our journal encourages inclusion of *data citations in the reference list* to directly cite datasets that were re-used and obtained from public databases. Data citations in the article text are distinct from normal bibliographical citations and should directly link to the database records from which the data can be accessed. In the main text, data citations are formatted as follows: "Data ref: Smith et al, 2001" or "Data ref: NCBI Sequence Read Archive PRJNA342805, 2017". In the Reference list, data citations must be labeled with "[DATASET]". A data reference must provide the database name, accession number/identifiers and a resolvable link to the landing page from which the data can be accessed at the end of the reference. Further instructions are available at .

12) All Materials and Methods need to be described in the main text using our 'Structured Methods' format. According to this format, the Methods section includes a Reagents and Tools Table (listing key reagents, experimental models, software and relevant equipment and including their sources and relevant identifiers) followed by a Methods and Protocols section describing the methods, ideally using a step-by-step protocol format. The aim is to facilitate adoption of the methodologies across labs. Please download and fill our Reagents and Tools Table template (.docx), which you can find in our author guidelines: https://www.embopress.org/page/journal/14693178/authorguide#structuredmethods.
When submitting your revised manuscript, please do not include the Reagents and Tools Table in the Methods section of the manuscript but upload it as a separate file choosing the file type "Reagent Table".
An example of a Method paper with Structured Methods can be found here:
https://www.embopress.org/doi/10.15252/msb.20178071.

13) As part of the EMBO publication's Transparent Editorial Process, EMBO Reports publishes online a Review Process File to accompany accepted manuscripts. This File will be published in conjunction with your paper and will include the referee reports, your point-by-point response and all pertinent correspondence relating to the manuscript.

You are able to opt out of this by letting the editorial office know (emboreports@embo.org). If you do opt out, the Review Process File link will point to the following statement: "No Review Process File is available with this article, as the authors have chosen not to make the review process public in this case."

14) CRediT has replaced the traditional author contributions section because it offers a systematic machine readable author contributions format that allows for more effective research assessment. Please remove the Author Contributions section from the manuscript and use the free text boxes beneath each contributing author's name in our online systems to add specific details on the author's contribution. More information is available in our guide to authors.

15) Please rename the Conflict of Interest section into "Disclosure and Competing Interests Statement", in accordance with our updated Guide to Authors (https://www.embopress.org/competing-interests)

16) Please make sure to all relevant funding information in the manuscript is also entered into our submission system. The funding information in the manuscript needs to be congruent with the info entered into our online system.

17) On the abstract page of the manuscript, please include 4-5 general keyword terms to enhance searchability.

We would also welcome the submission of cover suggestions, or motifs to be used by our Graphics Illustrator in designing a cover.

I look forward to seeing a revised form of your manuscript when it is ready and please let me know if you have questions or comments regarding the revision.

Yours sincerely,

Kurt Weir
Editor
EMBO Reports

Referee #1:

The authors have (very) comprehensively revised their manuscript, which all the reviewers agree is highly significant.

Referee #2:

Compared to the other reviewers, I have been quite critical and somewhat anankastic on this manuscript because the effects of biological reagents like the CoA can be strongly influenced by the exact composition and impurities. The authors have taken great effort to answer seriously and comprehensively all my comments, including to provide additional experimental data to clarify several questions. In most cases, my questions have been convincingly answered. For some comments, I would like to respond:

Comment #3. The data provided show that as little as 62.5 µM can activate a proinflammatory response, which suggests that the release of intracellular CoA during cell death can easily yield sufficient extracellular ligand for TLR4. As the title of the manuscript states that CoA acts through Myd88 and its property as a ligand for TLR4 is claimed as the discovery in the abstract, I think this is an important piece of evidence supporting the potential relevance of the work. The new data on human MDM is indeed very interesting and supportive as it stands.
Comment #4. The authors argute that the effect of CoA is "not due to a contaminating TLR4 agonist in the yeast-derived preparation (from Sigma-Aldrich; ~85% pure) given a negative Limulus Test". The Limulus test measures LPS but not other TLR4 agonists that may be present in the yeast-derived CoA prep. Thus, it is good to perform it, but will not exclude the presence of all TLR4 ligands. I like the direct comparison and TLR4 reporter cell assay in Supplemental Figure S4c/d, it adds data that support the manuscript's main points but also shows the complexity and importance of which exact reagents are used.
Comment #5. As far as I can see, the data show that CoA is a TLR4 agonist and induces proinflammatory gene expression largely in a Myd88-dependent mechanism, but the enhancement of IL-4-induced gene expression by CoA is not significantly reduced in Myd88-deficient BMDM. This is only based on CD206/CD301 flow cytometry data (Supplemental Fig. 6), no data for IL-4-induced gene expression in CoA-treated Myd88-KO BMDM are provided.
Therefore, the title stating that CoA enhances alternative macrophage activation via Myd88-signaling is misleading. If the authors want to keep it, then they should check whether other IL-4 readouts are enhanced by CoA in a Myd88-dependent manner (e.g. Ccl8, Mgl2 or others shown in Fig. 1).
In the absence of such data, the title should be adapted to better fit the results, e.g. stressing that "TLR4 agonistic activity of the metabolic cofactor CoA enhances alternative macrophage activation shared with Myd88-dependent PAMPs".
All other responses are well taken and I have no further comment.

***
Rev_Com_number: N/a
New_manu_number: EMBOR-2025-62896-T
Corr_author: Divakaruni
Title: The metabolic cofactor Coenzyme A enhances alternative macrophage activation via MyD88-linked signaling

RESPONSE TO REVIEWERS

Our remarks in **black**; reviewer comments in *red italics*

Prior to the addressing comments from the re-review, we would again like to sincerely thank all the peer reviewers for the time taken to review and improve our manuscript. We would also like to thank the staff at *Review Commons* and *EMBO Reports* for assistance with our manuscript submission.

Referee #1:
The authors have (very) comprehensively revised their manuscript, which all the reviewers agree is highly significant.

We thank the reviewer for their positive comments and kind words about the effort put into the resubmission.

Referee #2:
Compared to the other reviewers, I have been quite critical and somewhat anankastic on this manuscript because the effects of biological reagents like the CoA can be strongly influenced by the exact composition and impurities. The authors have taken great effort to answer seriously and comprehensively all my comments, including to provide additional experimental data to clarify several questions. In most cases, my questions have been convincingly answered. For some comments, I would like to respond:

We are grateful to the reviewer for their kind comments regarding the seriousness and thoroughness with which we addressed the previous comments. Moreover, we entirely understand the nature of the review and the specificity and attention to specific details of the reviewer's comments. These were important issues to clarify during the review process and the comments have improved our manuscript considerably from its earlier version.

Comment #3. The data provided show that as little as 62.5 µM can activate a proinflammatory response, which suggests that the release of intracellular CoA during cell death can easily yield sufficient extracellular ligand for TLR4. As the title of the manuscript states that CoA acts through Myd88 and its property as a ligand for TLR4 is claimed as the discovery in the abstract, I think this is an important piece of evidence supporting the potential relevance of the work. The new data on human MDM is indeed very interesting and supportive as it stands.

We are glad the reviewer shares our excitement about both the human macrophage response to CoA as well as the concentration-response data in mouse macrophages. We are pleased that these are seen as strong evidence to support the scientific claims made in the manuscript and the implicated relevance to physiology.

Comment #4. The authors argute that the effect of CoA is "not due to a contaminating TLR4 agonist in the yeast-derived preparation (from Sigma-Aldrich; ~85% pure) given a negative Limulus Test". The Limulus test measures LPS but not other TLR4 agonists that may be present in the yeast-derived CoA prep. Thus, it is good to perform it, but will not exclude the presence of all TLR4 ligands. I like the direct comparison and TLR4 reporter cell assay in Supplemental Figure S4c/d, it adds data that support the manuscript's main points but also shows the complexity and importance of which exact reagents are used.

We are glad the reviewer found our multiple, complimentary approaches are sufficient to prove that the effect of CoA is not due to a contaminating TLR4 agonist in the commercially purchased chemical. To further address the specificity of language, we note that our manuscript text states the Limulus assay tests for endotoxin specifically and not all TLR4 agonists (hence conducting the head-to-head comparison of yeast-derived and synthetic CoA during the revision).

Comment #5. As far as I can see, the data show that CoA is a TLR4 agonist and induces proinflammatory gene expression largely in a Myd88-dependent mechanism, but the enhancement of IL-4-induced gene expression by CoA is not significantly reduced in Myd88-deficient BMDM. This is only based on CD206/CD301 flow cytometry data (Supplemental Fig. 6), no data for IL-4-induced gene expression in CoA-treated Myd88-KO BMDM are provided.
Therefore, the title stating that CoA enhances alternative macrophage activation via Myd88-signaling is misleading. If the authors want to keep it, then they should check whether other IL-4 readouts are enhanced by CoA in a Myd88-dependent manner (e.g. Ccl8, Mgl2 or others shown in Fig. 1).

In the absence of such data, the title should be adapted to better fit the results, e.g. stressing that "TLR4 agonistic activity of the metabolic cofactor CoA enhances alternative macrophage activation shared with Myd88-dependent PAMPs".

We thank the reviewer for their comments and suggesting we be more precise with our language given the experiments in the Supplemental Figure 6. As indicated before, we are happy to change the title of the manuscript to better reflect the nuance of the findings, though we again note that the effect of CoA (and imiquimod) persisting in the absence of MyD88 simply shows that CoA does not *specifically* and *uniquely* activate the IL-4 response via MyD88. Nonetheless, we definitely agree with the reviewer that the title of the manuscript can be easily changed. As such, we propose the title "*Coenzyme A activates TLR4 and enhances alternative macrophage activation in line with MyD88 agonism*," a shortened version of the one suggested by the reviewer but capturing precisely the same nuance.

All other responses are well taken and I have no further comment.

We again thank the reviewer for their thorough review and helping improve the final version of our manuscript.

Dear Dr. Divakaruni

Thank you for the submission of your revised manuscript to EMBO reports.

From the editorial side, there are a few things that we need before we can proceed with the official acceptance of your study:

- We received bounced email alerts for contributing author Carolina Chavez - carolinachavez@mednet.ucla.edu. The author should either be removed from the author list in the system and added back using her new email address or you can send us the new email address and we will update the author's account accordingly.

- As a standard procedure, we edit the abstract of manuscripts to make them more accessible to a general readership. Please find the suggested version below my signature, which has been edited to present tense.

- Please rename the Materials and Methods section to Methods.

- Please remove the instructions from the Reagents and Tools table.

- Please expand on the animal housing conditions in the Methods section.

- Please provde the approval number from the authority granting approval to the animal experiments.

- Please adjust the format of the reference list and of the in-text citations according to EMBO Journal format (alphabetical order, up to 10 author names in the reference list before et al). Please refer to our Guide to Authors for additional information on EMBO J reference format.

- Please also adjust the format for citation of preprints as specified in our author guidelines:
The citation in the text should be: "(preprint: NAME1 et al, YEAR)"
The citation in the reference list: "Author NAME1, Author NAME2, ... (YEAR) article title. PLATFORM NAME, doi: XXX [PREPRINT]"
Please, refer to our detailed online guidelines for further information:
https://www.embopress.org/page/journal/14602075/authorguide#referencesformat

- The Methods sections "Isolation of human PBMC-derived macrophages", "Cell counts and normalization", and "ATAC-seq library prep" displayed high similarity to prior publications. Please proved a citation for these sections.

- 4814 is provided in the system for Agilent Technologies, but it is missing in the Acknowledgments. Please correct this discrepancy.

- Supplementary Table S1 is called out but missing. Additionally, this is not a correct callout as "supplementary" should not be used.

- Please update the titles of figures in the system to Figure 1, etc. and Figure EV1, etc from Main Figure 1, etc. and Expanded View Figure EV1, etc.

- Please note that EMBO press papers are accompanied online by:
A) a short (2 sentences) summary of the findings and their significance,
B) 2-5 short bullet points highlighting the key results, and
C) a synopsis image in .jpg or .png format that is exactly 550 pixels wide and 300 pixels high. Please note that the text needs to be legible at the final size. Please upload this information along with your revised manuscript (the text for A and B should be provided in one separate Word file uploaded as Cover Art/Synopsis Image).

- Please note that we require that plots with n<5 display individual data points. Please doublecheck figures EV1-4 for this.

- Our production/data editors have asked you to clarify several points in the figure legends (see below). Please incorporate these changes in the manuscript and return the revised file with tracked changes with your final manuscript submission.
- Please note that the exact p values are not provided in the legends of 1A,C; 2C,D,G,J; 3C; 4A,C,D,E; 5B,F; EV-1A,B,C; EV-2D,E; EV-3A,B,F; EV-4A-E.
- Please indicate the statistical test used for data analysis in the legends of figures EV-1A,B,C; EV-2D,E; EV-3A,B,F; EV-4A-E.
- Please note that information related to n is missing in the legends 3A.

We look forward to seeing a final version of your manuscript as soon as possible.

With kind regards,
Kurt Weir
Editor
EMBO Reports

Metabolites and metabolic co-factors can shape the innate immune response, though the pathways by which these molecules adjust inflammation remain incompletely understood. Here we show that the metabolic cofactor Coenzyme A (CoA) enhances IL-4 driven alternative macrophage activation [M(IL-4)] in vitro and in vivo. Unexpectedly, we find that perturbations in intracellular CoA metabolism do not influence M(IL-4) differentiation. Rather, we discover that exogenous CoA is a weak TLR4 agonist which primes macrophages for increased receptivity to IL-4 signals and resolution of inflammation via MyD88. Mechanistic studies reveal MyD88-linked signals enhance IL-4 responsiveness, in part, by reshaping chromatin accessibility to enhance transcription of IL-4-linked genes. The results identify CoA as a host metabolic co-factor that influences macrophage function through an extrinsic TLR4-dependent mechanism and suggest that damage-associated molecular patterns (DAMPs) can prime macrophages for alternative activation and resolution of inflammation.

***

Rev_Com_number: N/a
New_manu_number: EMBOR-2025-62896V2
Corr_author: Divakaruni
Title: Coenzyme A activates TLR4 and enhances alternative macrophage activation in line with MyD88 agonism

POINT-BY-POINT REVIEWER RESPONSE

Our responses are provided in black to the editorial queries reproduced here in blue italics

From the editorial side, there are a few things that we need before we can proceed with the official acceptance of your study:

- *We received bounced email alerts for contributing author Carolina Chavez - carolinachavez@mednet.ucla.edu. The author should either be removed from the author list in the system and added back using her new email address or you can send us the new email address and we will update the author's account accordingly.*

Thank you for letting us know. We have now listed Ms. Chavez's current email (chcarolina09@gmail.com) in the online submission portal.

- *As a standard procedure, we edit the abstract of manuscripts to make them more accessible to a general readership. Please find the suggested version below my signature, which has been edited to present tense.*

Thank you for the edits and switching from past to present tense. We agree with all changes and have copied the corrected abstract into both the manuscript text as well as the submission portal.

- *Please rename the Materials and Methods section to Methods.*

We have done so in the main copy of the text. We apologize for this oversight.

- *Please remove the instructions from the Reagents and Tools table.*

We have done so in the version that has been reuploaded in the submission portal.

- *Please expand on the animal housing conditions in the Methods section*
- *Please provide the approval number from the authority granting approval to the animal experiments.*

We apologize for this oversight. We have amended the text as shown below:

> *"All animal housing and experimental procedures were approved by the UCLA Animal Research Committee (ARC) under protocol ARC 2020-027 and conducted in accordance with the NIH Guide for the Care and Use of Laboratory Animals. Mice were group housed (four per cage) and maintained under a standard 12-hour light-dark cycle (lights on 7:00-19:00) in a humidity-controlled colony room. Animals were given ad libitum access to water and fed a standard chow diet. Animals were given environmental enrichments and UCLA veterinary staff provided weekly assessments of general animal health."*

- *Please adjust the format of the reference list and of the in-text citations according to EMBO Journal format (alphabetical order, up to 10 author names in the reference list before et al). Please refer to our Guide to Authors for additional information on EMBO J reference format.*

We sincerely apologize for this oversight in our previous submission. We have now changed all references accordingly. This was done using the Word Mendeley plugin along with the most up-to-date EMBO J reference format .csl library. Some references were manually adjusted for alphabetical order and author initials to keep in line with the journal format (using our prior submission to the journal in 2025 as a guide).

- Please also adjust the format for citation of preprints as specified in our author guidelines:
The citation in the text should be: "(preprint: NAME1 et al, YEAR)"
The citation in the reference list: "Author NAME1, Author NAME2, ... (YEAR) article title. PLATFORM NAME, doi: XXX [PREPRINT]"
Please, refer to our detailed online guidelines for further information: https://www.embopress.org/page/journal/14602075/authorguide#referencesformat

Thank you for providing this information. We have now done this appropriately for the two bioRxiv preprints cited in our paper. Please note that GA Timblin *et al*. was published in eLife as a reviewed preprint. It is cited as such in its citation in the current format from the journal's citation format (eLife 14:RP106473).

- *The Methods sections "Isolation of human PBMC-derived macrophages", "Cell counts and normalization", and "ATAC-seq library prep" displayed high similarity to prior publications. Please proved a citation for these sections.*

Thank you for pointing this out, we have now amended these sections as indicated in the highlighted text with the following appropriate references, two of which are our group's prior publications:

Ball AB et al. (2025) EMBO Rep. 2025 Feb;26(4):982-1002
Buenrostro JD et al. (2015) Curr Protoc Mol Biol. 2015 109:21.29.1-21.29.9.
Cheng MI et al. (2023) Nat Immunol. 24(5):780-791.
Divakaruni AS et al. (2018) Cell Metab. 28(3):490-503

- *4814 is provided in the system for Agilent Technologies, but it is missing in the Acknowledgments. Please correct this discrepancy.*

Thank you for catching this oversight. This award identifier has been added to the text.

- *Supplementary Table S1 is called out but missing. Additionally, this is not a correct callout as "supplementary" should not be used.*

Thank you for notifying us of this error. We have removed this callout entirely, as the Table contained primer sequences for qPCR analysis that are available in the Reagents and Tools Table. As such, they need not be listed in an additional table. This was a holdover error from the format-blind initial submission, and we apologize.

- *Please update the titles of figures in the system to Figure 1, etc. and Figure EV1, etc from Main Figure 1, etc. and Expanded View Figure EV1, etc.*

This has been done. Thank you.

- *Please note that we require that plots with n<5 display individual data points. Please doublecheck figures EV1-4 for this.*

Thank you for this note as we were previously unaware this requirement also applied to EV Figures. We have now entirely replaced the Expanded View Figures 1-4 with data showing individual data points for all graphs regardless of how many individual data points are presented. The notable exceptions are Figs. EV3 B,D,& E. Like Figure 4B, these are concentration-response curves showing several points along a logarithmic axis, for which showing the individual data points would be inappropriate and highly distracting. As such, we have now included source data also for all Extended View Figures so, like for Figure 4B, readers of the manuscript have access to the individual data points for our concentration-response curves.

- *Our production/data editors have asked you to clarify several points in the figure legends (see below). Please incorporate these changes in the manuscript and return the revised file with tracked changes with your final manuscript submission:*
    - *Please note that the exact p values are not provided in the legends of 1A,C; 2C,D,G,J; 3C; 4A,C,D,E; 5B,F; EV-1A,B,C; EV-2D,E; EV-3A,B,F; EV-4A-E.*

Thank you for this note, as we have now provided exact p values for all figures where $0.001<p<0.05$. We note that in prior correspondence with Dr. Esther Schnapp (filling in for Dr. Weir while on vacation), we inquired what cutoff was appropriate and how many significant figures were required to comply with journal standards. She clarified that it was appropriate to leave designations of '***, P<0.001', as there was little to be gained from exact values where $p<0.001$. We note that these were also the guidelines in our recent, prior publication with *EMBO Reports* as well.

-
    - *Please indicate the statistical test used for data analysis in the legends of figures EV-1A,B,C; EV-2D,E; EV-3A,B,F; EV-4A-E.*

Thank you for this note, as we have now provided the statistical test used for our EV figures, though we note that there is no statistical significance reported for Fig. EV3B (representative concentration response curve), so no test is given.

- *Please note that information related to n is missing in the legends 3A.*

We apologize for this oversight, and have now included the number of biological replicates for both Fig. 3A (RNA-Seq) and 6A (ATAC-Seq).

- *Please note that EMBO press papers are accompanied online by:*
  A) a short (2 sentences) summary of the findings and their significance,
  B) 2-5 short bullet points highlighting the key results,
  C) and a synopsis image in .jpg or .png format that is exactly 550 pixels wide and 300 pixels high. Please note that the text needs to be legible at the final size. Please upload this information along with your revised manuscript (the text for A and B should be provided in one separate Word file uploaded as Cover Art/Synopsis Image).

Thank you for letting us know. A document with a two-sentence summary/blurb as well as four bullet points has been uploaded to the drive in a single Word document. In addition, we have also generated a synopsis/graphical abstract for the piece and have uploaded this as well as the BioRender publication license.

2nd Revision - Editorial Decision 25th Aug 2026

Ajit Divakaruni
University of California, Los Angeles
Department of Molecular and Medical Pharmacology;
650 Charles E Young Dr. S
Los Angeles, CA 90095
United States

Dear Ajit,

I am very pleased to accept your manuscript for publication in the next available issue of EMBO reports. Thank you for your contribution to our journal.

Your manuscript will be processed for publication by EMBO Press. It will be copy edited and you will receive page proofs prior to publication. Please note that you will be contacted by Springer Nature Author Services to complete licensing and payment information.

You may qualify for financial assistance for your publication charges - either via a Springer Nature fully open access agreement or an EMBO initiative. Check your eligibility: https://link.springer.com/journal/44319/how-to-publish-with-us

Should you be planning a Press Release on your article, please notify the production team via this form in order to coordinate publication and release dates: https://forms.cloud.microsoft/e/eH4A8JVP6g

If you have any questions, please do not hesitate to contact the Editorial Office. Thank you for your contribution to EMBO Reports.

Best regards,
Esther

Esther Schnapp, PhD
Senior Editor
EMBO reports

>>> Please note that it is EMBO Reports policy for the transcript of the editorial process (containing referee reports and your response letter) to be published as an online supplement to each paper. If you do NOT want this, you will need to inform the Editorial Office via email immediately. More information is available here: https://link.springer.com/partners/embo-press/editorial-policies#Peer%20review

EMBO Press Author Checklist

Corresponding Author Name: Ajit S. Divakaruni	**USEFUL LINKS FOR COMPLETING THIS FORM**
Journal Submitted to: EMBO Reports	The EMBO Journal - Author Guidelines
Manuscript Number: EMBOR-2025-62896-T	EMBO Reports - Author Guidelines
	Molecular Systems Biology - Author Guidelines
	EMBO Molecular Medicine - Author Guidelines

Reporting Checklist for Life Science Articles (updated January
This checklist is adapted from Materials Design Analysis Reporting (MDAR) Checklist for Authors. MDAR establishes a minimum set of requirements in transparent reporting in the life sciences (see Statement of Task: 10.31222/osf.io/9sm4x). Please follow the journal's guidelines in preparing your
Please note that a copy of this checklist will be published alongside your article.

Abridged guidelines for figures
1. Data
The data shown in figures should satisfy the following conditions:

- the data were obtained and processed according to the field's best practice and are presented to reflect the results of the experiments in an accurate and unbiased manner.
- ideally, figure panels should include only measurements that are directly comparable to each other and obtained with the same assay.
- plots include clearly labeled error bars for independent experiments and sample sizes. Unless justified, error bars should not be shown for technical
- if n<5, the individual data points from each experiment should be plotted. Any statistical test employed should be justified.
- Source Data should be included to report the data underlying figures according to the guidelines set out in the authorship guidelines on Data

2. Captions
Each figure caption should contain the following information, for each panel where they are relevant:

- a specification of the experimental system investigated (eg cell line, species name).
- the assay(s) and method(s) used to carry out the reported observations and measurements.
- an explicit mention of the biological and chemical entity(ies) that are being measured.
- an explicit mention of the biological and chemical entity(ies) that are altered/varied/perturbed in a controlled manner.
- the exact sample size (n) for each experimental group/condition, given as a number, not a range;
- a description of the sample collection allowing the reader to understand whether the samples represent technical or biological replicates (including how many animals, litters, cultures, etc.).
- a statement of how many times the experiment shown was independently replicated in the laboratory.
- definitions of statistical methods and measures:
  - common tests, such as t-test (please specify whether paired vs. unpaired), simple χ2 tests, Wilcoxon and Mann-Whitney tests, can be unambiguously identified by name only, but more complex techniques should be described in the methods section;
  - are tests one-sided or two-sided?
  - are there adjustments for multiple comparisons?
  - exact statistical test results, e.g., P values = x but not P values < x;
  - definition of 'center values' as median or average;
  - definition of error bars as s.d. or s.e.m.

Please complete ALL of the questions below.
Select "Not Applicable" only when the requested information is not relevant for your study.

Materials

Newly Created Materials	Information included in the manuscript?	In which section is the information available? (Reagents and Tools Table, Materials and Methods, Figures, Data Availability Section)
New materials and reagents need to be available; do any restrictions apply?	Not Applicable	

Antibodies	Information included in the manuscript?	In which section is the information available? (Reagents and Tools Table, Materials and Methods, Figures, Data Availability Section)
For **antibodies** provide the following information:<br>- Commercial antibodies: RRID (if possible) or supplier name, catalogue number and or/clone number<br>- Non-commercial: RRID or citation	Yes	Reagents and Tools Table

DNA and RNA sequences	Information included in the manuscript?	In which section is the information available? (Reagents and Tools Table, Materials and Methods, Figures, Data Availability Section)
**Short novel DNA or RNA including primers, probes**: provide the sequences.	Yes	Reagents and Tools Table

Cell materials	Information included in the manuscript?	In which section is the information available? (Reagents and Tools Table, Materials and Methods, Figures, Data Availability Section)
**Cell lines:** Provide species information, strain. Provide accession number in repository **OR** supplier name, catalog number, clone number, and/**OR** RRID.	Yes	Reagents and Tools Table
**Primary cultures:** Provide species, strain, sex of origin, genetic modification status.	Yes	Reagents and Tools Table
Report if the cell lines were recently **authenticated** (e.g., by STR profiling) and tested for mycoplasma contamination.	Yes	Materials and Methods

Experimental animals	Information included in the manuscript?	In which section is the information available? (Reagents and Tools Table, Materials and Methods, Figures, Data Availability Section)
**Laboratory animals or Model organisms:** Provide species, strain, sex, age, genetic modification status. Provide accession number in repository **OR** supplier name, catalog number, clone number, **OR** RRID.	Yes	Reagents and Tools Table
**Animal observed in or captured from the field:** Provide species, sex, and age where possible.	Not Applicable	
Please detail **housing and husbandry conditions**.	Yes	Materials and Methods

Plants and microbes	Information included in the manuscript?	In which section is the information available? (Reagents and Tools Table, Materials and Methods, Figures, Data Availability Section)
**Plants:** provide species and strain, ecotype and cultivar where relevant, unique accession number if available, and source (including location for collected wild specimens).	Not Applicable	
**Microbes:** provide species and strain, unique accession number if available, and source.	Not Applicable	

Human research participants	Information included in the manuscript?	In which section is the information available? (Reagents and Tools Table, Materials and Methods, Figures, Data Availability Section)
If collected and within the bounds of privacy constraints report on age, sex and gender or ethnicity for all study participants.	Not Applicable	

Core facilities	Information included in the manuscript?	In which section is the information available? (Reagents and Tools Table, Materials and Methods, Figures, Data Availability Section)
If your work benefited from core facilities, was their service mentioned in the acknowledgments section?	Yes	Acknowledgements section

Design

Study protocol	Information included in the manuscript?	In which section is the information available? (Reagents and Tools Table, Materials and Methods, Figures, Data Availability Section)
If study protocol has been **pre-registered, provide DOI in the manuscript**. For clinical trials, provide the trial registration number **OR** cite DOI.	Not Applicable	
Report the **clinical trial registration number** (at ClinicalTrials.gov or equivalent), where applicable.	Not Applicable	

Laboratory protocol	Information included in the manuscript?	In which section is the information available? (Reagents and Tools Table, Materials and Methods, Figures, Data Availability Section)
Provide DOI OR other citation details if **external detailed step-by-step protocols** are available.	Yes	References

Experimental study design and statistics	Information included in the manuscript?	In which section is the information available? (Reagents and Tools Table, Materials and Methods, Figures, Data Availability Section)
Include a statement about **sample size** estimate even if no statistical methods were used.	Yes	
Were any steps taken to minimize the effects of subjective bias when allocating animals/samples to treatment (e.g. **randomization procedure**)? If yes, have they been described?	Yes	
Include a statement about **blinding** even if no blinding was done.	Yes	
Describe **inclusion/exclusion criteria** if samples or animals were excluded from the analysis. Were the criteria pre-established?<br><br>If sample or data points were omitted from analysis, report if this was due to attrition or intentional exclusion and provide justification.	Not Applicable	
For every figure, are **statistical tests** justified as appropriate? Do the data meet the assumptions of the tests (e.g., normal distribution)? Describe any methods used to assess it. Is there an estimate of variation within each group of data? Is the variance similar between the groups that are being statistically compared?	Yes	Figures and Materials and Methods

Sample definition and in-laboratory replication	Information included in the manuscript?	In which section is the information available? (Reagents and Tools Table, Materials and Methods, Figures, Data Availability Section)
In the figure legends: state number of times the experiment was **replicated** in laboratory.	Yes	Figures
In the figure legends: define whether data describe **technical or biological replicates**.	Yes	Figures

Ethics

Ethics	Information included in the manuscript?	In which section is the information available? (Reagents and Tools Table, Materials and Methods, Figures, Data Availability Section)
Studies involving **human participants**: State details of **authority granting ethics approval** (IRB or equivalent committee(s), provide reference number for approval.	Not Applicable	
Studies involving **human participants**: Include a statement confirming that **informed consent** was obtained from all subjects and that the experiments conformed to the principles set out in the WMA Declaration of Helsinki and the Department of Health and Human Services Belmont Report.	Not Applicable	
Studies involving **human participants:** For publication of **patient photos**, include a statement confirming that consent to publish was obtained.	Not Applicable	
Studies involving experimental **animals**: State details of **authority granting ethics approval** (IRB or equivalent committee(s), provide reference number for approval. Include a statement of compliance with ethical regulations.	Yes	Materials and Methods
Studies involving **specimen and field samples:** State if relevant **permits** obtained, provide details of authority approving study; if none were required, explain why.	Not Applicable	

Dual Use Research of Concern (DURC)	Information included in the manuscript?	In which section is the information available? (Reagents and Tools Table, Materials and Methods, Figures, Data Availability Section)
Could your study fall under dual use research restrictions? Please check biosecurity documents and list of **select agents and toxins** (CDC): https://www.selectagents.gov/sat/list.htm	Not Applicable	
If you used a select agent, is the security level of the lab appropriate and reported in the manuscript?	Not Applicable	
If a study is subject to dual use research of concern regulations, is the name of the **authority granting approval and reference number** for the regulatory approval provided in the manuscript?	Not Applicable	

Reporting

The MDAR framework recommends adoption of discipline-specific guidelines, established and endorsed through community initiatives. Journals have their own policy about requiring specific guidelines and recommendations to complement MDAR.

Adherence to community standards	Information included in the manuscript?	In which section is the information available? (Reagents and Tools Table, Materials and Methods, Figures, Data Availability Section)
State if relevant guidelines or checklists (e.g., **ICMJE, MIBBI, ARRIVE, PRISMA**) have been followed or provided.	Not Applicable	
For **tumor marker prognostic studies,** we recommend that you follow the **REMARK** reporting guidelines (see link list at top right). See author guidelines, under 'Reporting Guidelines'. Please confirm you have followed these guidelines.	Not Applicable	
For **phase II and III randomized controlled trials**, please refer to the **CONSORT** flow diagram (see link list at top right) and submit the CONSORT checklist (see link list at top right) with your submission. See author guidelines, under 'Reporting Guidelines'. Please confirm you have submitted this list.	Not Applicable	

Data Availability

Data availability	Information included in the manuscript?	In which section is the information available? (Reagents and Tools Table, Materials and Methods, Figures, Data Availability Section)
Have **primary datasets** been deposited according to the journal's guidelines (see 'Data Deposition' section) and the respective accession numbers provided in the Data Availability Section?	Yes	Data Availability Section
Were **human clinical and genomic datasets** deposited in a public access-controlled repository in accordance to ethical obligations to the patients and to the applicable consent agreement?	Not Applicable	
Are **computational models** that are central and integral to a study available without restrictions in a machine-readable form? Were the relevant accession numbers or links provided?	Not Applicable	
If publicly available data were reused, provide the respective **data citations in the reference list**.	Not Applicable	
