# Supplementary material for: Coenzyme A activates TLR4 and enhances alternative macrophage activation in line with MyD88 agonism
Source: EMBO Rep. 2026 Sep 18;27(20):5927–52. doi: 10.1038/s44319-026-00925-y (PMC13694274; doi:10.1038/s44319-026-00925-y)
Supplement: Supplementary file 4 [file 44319_2026_925_MOESM2_ESM.zip › Figure 1/1E/README.rtf]

This file contains representative images for in vitro phagocytosis assays.
